# The Complete Mitochondrial Genome of the Booklouse, *Liposcelis decolor*: Insights into Gene Arrangement and Genome Organization within the Genus *Liposcelis*

**DOI:** 10.1371/journal.pone.0091902

**Published:** 2014-03-17

**Authors:** Shi-Chun Chen, Dan-Dan Wei, Renfu Shao, Wei Dou, Jin-Jun Wang

**Affiliations:** 1 Key Laboratory of Entomology and Pest Control Engineering, College of Plant Protection, Southwest University, Chongqing, P. R. China; 2 GeneCology Research Centre, Faculty of Science, Education and Engineering, University of the Sunshine Coast, Maroochydore, Queensland, Australia; Tel-Aviv University, Israel

## Abstract

Booklice in the genus *Liposcelis* are pests of stored grain products. They pose a considerable economic threat to global food security and safety. To date, the complete mitochondrial genome has only been determined for a single booklouse species *Liposcelis bostrychophila.* Unlike most bilateral animals, which have their 37 mt genes on one circular chromosome, ≈15 kb in size, the mt genome of *L. bostrychophila* has two circular chromosomes, 8 and 8.5 kb in size. Here, we report the mt genome of another booklouse, *Liposcelis decolor*. The mt genome of *L. decolor* has the typical mt chromosome of bilateral animals, 14,405 bp long with 37 genes (13 PCGs, 22 tRNAs and 2 rRNAs). However, the arrangement of these genes in *L. decolor* differs substantially from that observed in *L. bostrychophila* and other insects. With the exception of *atp8-atp6*, *L. decolor* differs from *L. bostrychophila* in the arrangement of all of the other 35 genes. The variation in the mt genome organization and mt gene arrangement between the two *Liposcelis* species is unprecedented for closely related animals in the same genus. Furthermore, our results indicate that the two-chromosome mt genome organization observed in *L. bostrychophila* likely evolved recently after *L. bostrychophila* and *L. decolor* split from their most recent common ancestor.

## Introduction

Insect mitochondrial (mt) genomes usually consist of a single circular chromosome (13–20 kb) containing 13 protein-coding genes (PCGs), 22 transfer RNA genes (tRNAs) and two ribosomal RNA genes (rRNAs), which is typical of bilateral animals [1]. The mt genome usually contains one large non-coding element called the A+T-rich or control region, which contains the sites for genome replication and the initiation of gene transcription [1]. Due to several unique features, including conserved gene content, maternal inheritance, and rapid nucleotide evolution, mt genome sequences have been used to facilitate the understanding of animal evolution [2]. Currently, more than 300 insect mt genomes have been sequenced [3]. Various types of gene rearrangements have been observed in insect mt genomes. Rearrangements of tRNA genes are the most common whereas rearrangements of protein-coding and rRNA genes are less common [4]–[6]. Studies in recent years also revealed variations of mt genome organization in bilateral animals [1]. For instance, mt genomes that consist of multiple chromosomes have been reported in parasitic lice [7]–[9], booklice [3], rotifera [10] and nematodes [11]–[13]. Variations in mt genome organization may provide a novel perspective for understanding animal evolution [14]–[16], in addition to genome sequences [3], [17], RNA secondary structures [7], [18]–[20], and gene rearrangements [5], [18], [21], [22].

Several types of atypical mt genome organization have been reported in psocodean insects (superorder Psocodea) in recent years. Psocodea contains two orders of insects: Psocoptera (booklice and barklice) and Phthiraptera (chewing and sucking lice). The mt genomes of human lice, *Pediculus humanus*, *P. capitis* and *Pthirus pubis*, consist of 14 to 20 mini-chromosomes, each one is 1.8 to 4 kb in size and contains one to five genes [7], [9]. The chewing louse, *Coloceras* sp., has a typical mt chromosome with 37 genes and a circular mt DNA molecule that is approximately half the size of the typical mt chromosome [8]. The booklouse, *Liposcelis bostrychophila*, has a bipartite mt genome with two chromosomes: one chromosome is ≈8 kb in size and has 16 genes and the other is ≈8.5 kb in size and has 22 genes [3]. Extensive gene rearrangement has been found in the mt genomes of most of the 12 Psocodea species that have been completely or nearly completely sequenced to date, including the booklouse, *L. bostrychophila* [3].

During the last two decades, the booklice of the genus *Liposcelis* have emerged as serious pests of stored commodities worldwide [23], [24]. The genus *Liposcelis* has 126 known species worldwide and includes four groups (A, B, C, and D)[25], [26]. The booklouse. *L. decolor*, investigated in the current study, belongs to group C, while *L. bostrychophila* belongs to group D. Many previous studies have indicated that there is great variation among the four *Liposcelis* groups with respect to morphology, physiology, biochemistry and molecular biology [25], [27], [28]. In particular, analyses of ITS (internal transcribed spacers) sequences indicated that *Liposcelis* species of groups C and D have the highest nucleotide divergence among the four groups [25].

To understand whether the bipartite mt genome organization observed in *L. bostrychophila* occurred in other booklice of the genus *Liposcelis*, we sequenced the mt genome of *L. decolor*. We found that, unlike *L. bostrychophila*, *L. decolor* has the typical mt chromosome of bilateral animals. However, the arrangement of mt genes in *L. decolor* differs substantially from that in *L. bostrychophila* and other known insects. Our results showed, for the first time, a high level of variation in both mt genome organization and mt gene arrangement between closely related animal species in the same genus.

## Materials and Methods

### Ethics statement

No specific permits were required for the insects collected in this study. The sampling locations were not privately owned or protected in any way and the collection did not involve endangered or protected species.

### Sample collection, DNA extraction, PCR and sequencing

*L. decolor* individuals were collected at grain storage facilities in Binzhou, Shandong Province, China in 2010, and identified to species according to their morphological characteristics [28]–[30]. Subsequently, the ITS sequence [25] and sequences of partial *rrnL* and *cox1* genes [31] were used to confirm the species identification. The ITS sequence obtained has been deposited in GenBank under accession number KF874610. An *L. decolor* colony was maintained in the lab on a diet of whole wheat flour, skim milk, and yeast powder (10∶1∶1) in an incubator at 27 ± 0.5°C, 75–80% relative humidity and a scotoperiod of 24 hours. Voucher specimens (#Ps-01-01-03) were deposited at the Insect Collection, Southwest University, Chongqing, China. Total genomic DNA was extracted using a Tissue/Cell gDNA Mini Kit (Watson Biotechnologies, Shanghai, China) and stored at −20°C. Parts of *cox1, cox3, cob, rrnS, rrnL* and *nad5* genes were amplified by PCR with conserved insect primers (Table S1) [32]. Species-specific primers were then designed for long PCR (Table S1).

Each long PCR reaction was performed in a 25 μL volume, containing 1 μL each of forward primer (10 μM) and reverse primer (10 μM), 4 μL of dNTPs (each 2.5 mM), 1 μL of template DNA (∼300 ng/μL), 2.5 μL MgCl_2_ (25 mM), 2.5 μL of 10× LA PCR reaction buffer, 12.75 μL ddH_2_O and 0.25 μL LA Taq DNA polymerase (5 U/μL, Takara). All reactions were carried out using C1000™ thermal cyclers (Bio-RAD, Hercules, CA, USA) with the follow conditions: initial 2 min denaturation at 94°C, 37 cycles of 94°C for 20 sec, 58°C for 50 sec, 68°C for 5–10 min (depending on target size, 1 min/kb), followed by a final extension at 68°C for 15 min. Gel-purified amplification products < 6 kb in size were ligated into pGEM-T Easy vectors (Promega, Madison, WI, USA), and introduced into Escherichia coli (Trans5α, TransGen Biotech, Beijing, China), following by ampicillin selection, then sequenced with M13 primers. Longer PCR products (> 6 kb) were directly sequenced with both forward and reverse PCR primers and internal primers by primer walking. All amplification products were sequenced with an ABI 3730 automated DNA sequencer (Applied Biosystems, Foster city, CA, USA) at the Beijing Genomics Institute (BGI) in Beijing, China.

### Sequence assembly, annotation and analysis

SeqMan (DNAStar) was used to assemble the two overlapping nucleotide sequences, which were further confirmed by manually inspection. The protein-coding and rRNA genes were identified using the program ORF Finder (http://www.ncbi.nlm.nih.gov/gorf/gorf.html) and BLAST searches against the GenBank database, respectively. Subsequently, all of these genes were further confirmed by alignment with homologous genes from those of other louse and booklouse species. The transfer RNA genes were identified by their cloverleaf secondary structure using ARWEN [33] with default parameters and tRNAscan-SE 1.21 [34] with Search Mode  =  “EufindtRNA-Cove”, Genetic Code  =  “Invertebrate Mito” and Cove score cutoff  =  0.1. The stem-loop secondary structure of the putative control regions was folded using the Mfold Server [35] under the RNA folding option with default parameters. The base composition and codon usage were analyzed with BioEdit (http://bioedit.software.informer.com/) and DAMBE 5.3.9 [36]. Sequences of mt genomes of other lice were retrieved from GenBank and MitoZoa (Table S2) [37].

### Phylogenetic analyses

Phylogenetic analyses were conducted with the 11 Psocodea mt genome sequences currently available in GenBank including the new booklouse sequence obtained in this study. The mt genome sequence of the fruit fly, *Drosophila melanogaster*, served as an outgroup. Sequences of *atp8*, *nad4L*, and tRNA genes were too short and too variable to be correctly aligned among the psocodean species; these genes were thus excluded from the phylogenetic analyses. The *nad4* was also excluded as this gene has not been identified in the human pubic louse, *P. pubis* [7]. The amino acid sequences from each protein-coding gene and the nucleotide sequence of each rRNA gene were aligned with MAFFT v7 [38]. The nucleotide sequences of each protein-coding gene were aligned based on the corresponding amino acid alignments using PAL2NAL [39] to ensure the correct reading frame; the poorly aligned sites were removed with GUIDANCE[40] using the default setting. Then, positions with gap in more than half of the species were removed. Substitution saturations of the nucleotide sequences were examined using DAMBE 5.3.9 following Xia et al. [41]. Whole PCG sequences were chosen to enter the next step if *I*ss (index of substitution saturation) is significantly lower than *I*ss.c (critical value for symmetrical tree topology) (*P* < 0.05). All of the protein-coding and rRNA genes, except *nad3* and *nad6*, passed this test. Consequently, the third codon positions of *nad3* and *nad6* were excluded from phylogenetic analyses. The best fit models for the alignment of nucleotide sequence and amino acid sequence were determined using the Akaike Information Criterion in jModelTest 0.1.1 [42] and ProtTest 3 [43], respectively. Specifically, the GTR+I+G model and MtREV+I+G model were chosen for the nucleotide sequence dataset and the amino acid sequence dataset, respectively. Phylogenetic trees were estimated via Bayesian inference (BI) method using MrBayes v3.12 [44]. Four independent Markov chains were simultaneously run for 2,000,000 generations with a heating scheme (temp  =  0.2). Trees were sampled every 100 generations (sample-freq  = 100) and the first 25% of the generations were discarded as burn-in and the remaining samples were used to compute the consensus tree. Stationarity was considered to be reached when the average standard deviation of split frequencies was below 0.01 [45].

## Results and Discussion

### Mitochondrial genome of *Liposcelis decolor*

**Figure 1:**
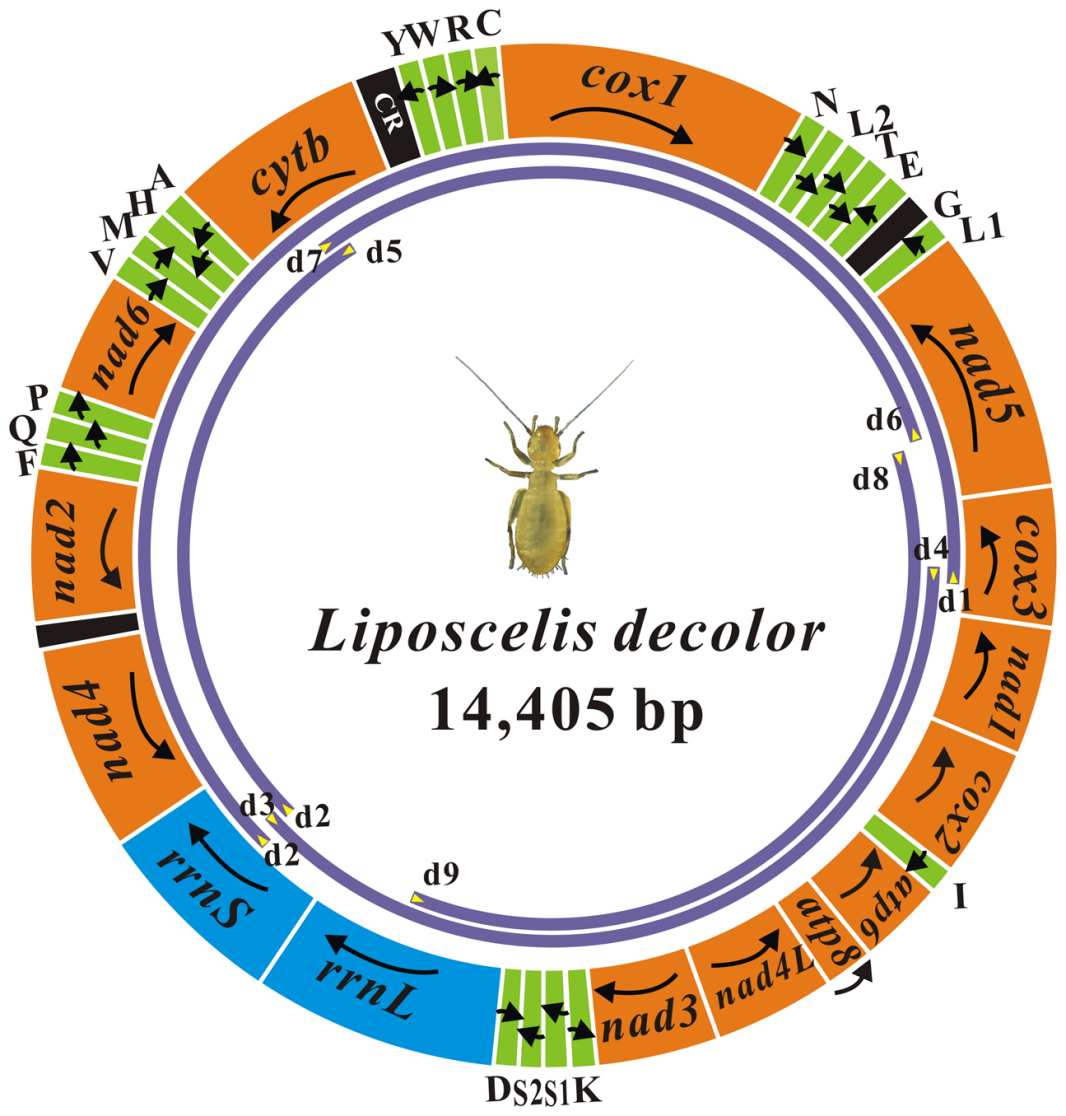
Mitochondrial genome of *Liposcelis decolor*. Transcriptional orientation is indicated with arrows. Protein-coding genes, ribosomal RNA genes and transfer RNA genes are shown in orange, blue and green respectively. tRNA genes for the two serine and two leucine tRNAs: S_1_  =  AGN, S_2_  =  UCN, L_1_  =  CUN, and L_2_  =  UUR. The non-coding regions larger than 60 bp are indicated in black. CR  =  putative control region. Arrows and purple curves indicate primers and PCR fragment, respectively. See Table S1 for sequence of PCR primers.

**Figure 2:**
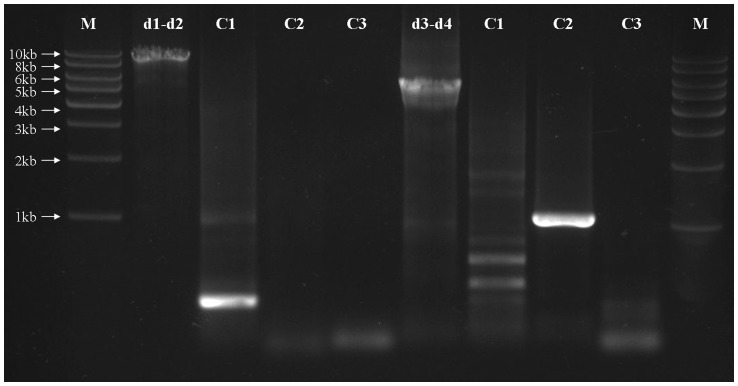
PCR amplification and verification of mitochondrial DNA of *Liposcelis decolor*. Long-PCR amplification of two fragments to verify the single circular mt genome in *L. decolor*. Lane C1, negative control without the forward primer d1 or d3; lane C2, negative control without the reverse primer d2 or d4; lane C3, negative control without the DNA template. Lane M: 1 kb marker (Biomed). “d1-d2”, the product of PCR with primers d1 and d2, etc. Primer details are given in Table S1.

The mt genome of *L. decolor* has one typical circular chromosome, unlike the booklouse, *L. bostrychophila* (Figure 1). The size and the circular organization of the mt chromosome of *L. decolor* was confirmed by two overlapping PCR amplicons, 9.1 kb (d1-d2 from *cox3* to *rrnS*) and 5.5 kb in size (d3-d4 from *rrnS* to *cox3*) respectively (Figure 1, Figure 2 and Table S1). The two amplicons overlapped by 33 bp in *cox3* and 92 bp in *rrnS*.

**Figure 3:**
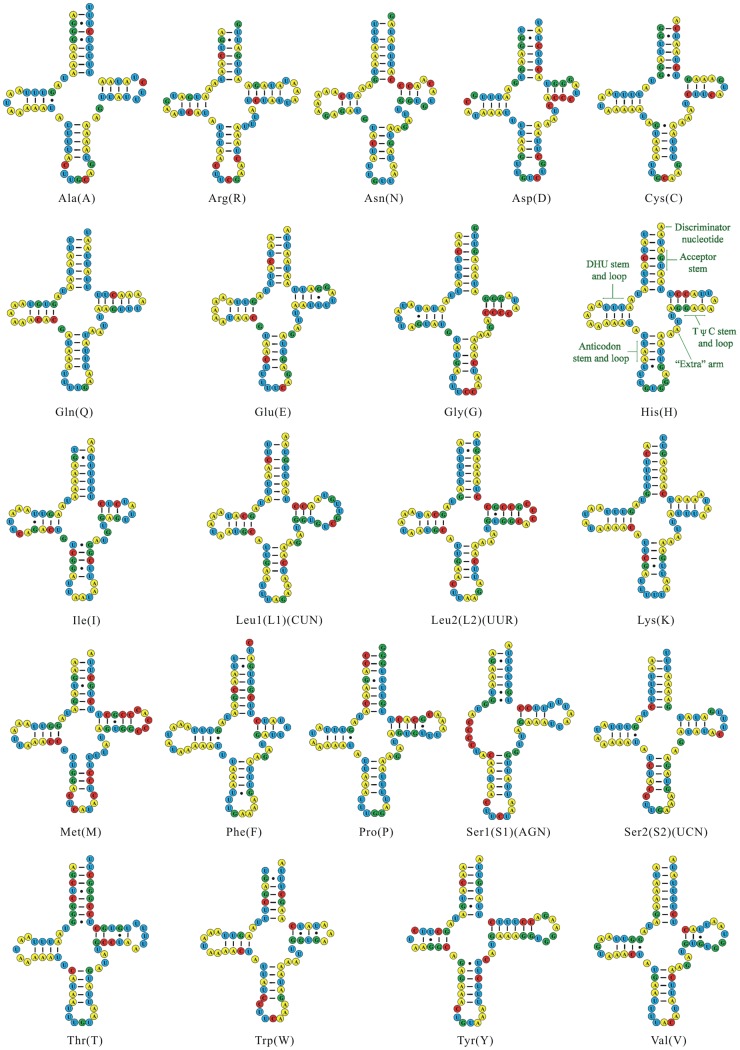
Putative secondary structures of the 22 tRNA genes identified in the mitochondrial genome of *Liposcelis decolor*. Bars indicate Watson-Crick base pairings, and dots between G and U pairs mark canonical base pairings in RNA.

**Figure 4:**
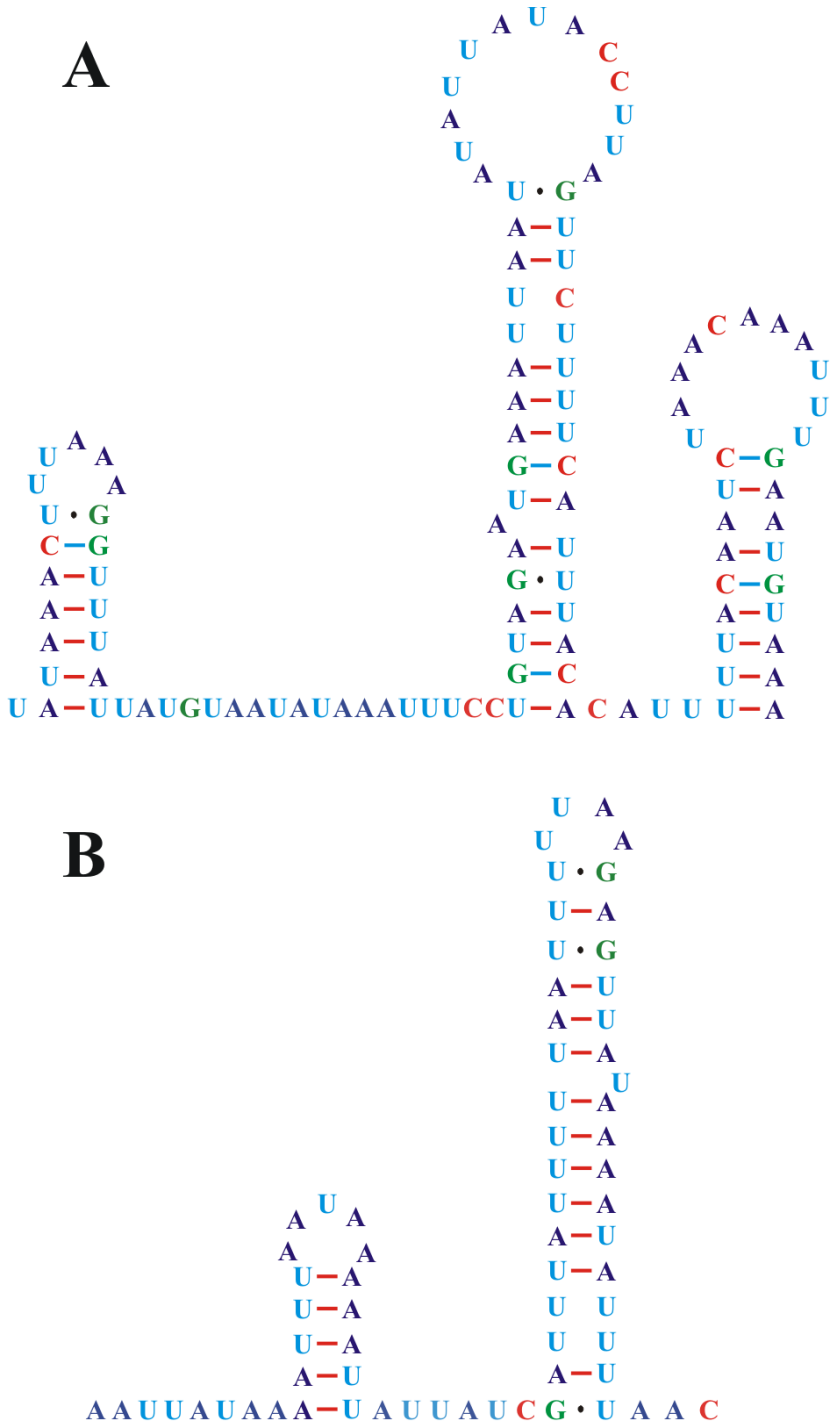
Stem-loop secondary structures of two non-coding regions of *Liposcelis decolor*. A, Stem-loop secondary structure of the 118 bp non-coding sequence; B, Stem-loop secondary structure of the 69 bp non-coding sequence.

Sequencing and assembly of these two PCR amplicons revealed that the mt genome of *L. decolor* is 14,405 bp in length and encodes 37 genes that are typically found in metazoan mt genomes (Figure 1 and Table S3) (GenBank accession number: JX870621). All of the protein-coding genes (PCGs) initiate translation at an ATN codon, except for the TTG codon used in *cox1*. TAA and TAG serve as stop codons for all of the PCGs (Table S4). Eleven of the 13 PCGs had average length for Psocodean species, but *nad4* is shorter and the *nad4L* is longer than in other Psocodean species (Figure S1). All 22 tRNA coding genes usually found in the mt genomes of metazoans are present in *L. decolor* (Figure 3); all have the conventional cloverleaf shaped secondary structure except *trnS_1_*, which lacks the D-arm, as in other insects. There are three non-coding regions longer than 60 bp in the mt genome of *L. decolor*. The longest non-coding region (118 bp) lies between *cob* and *trnY*, has an A+T content of 82.20%; three stem-loop secondary structures can be found in this region (Figure 4A). An 80 bp non-coding region lies between *nad2* and *nad4* with an 88.75% A+T content but has no stem-loop secondary structure. A 69 bp non-coding region is between *trnG* and *trnL_1_* with a 92.75% A+T content and has two stem-loops (Figure 4B). The 118-bp region is considered to be most likely the putative control region (Figure 1) due to it is longer sequence in the mt genome and the presence of typical loop secondary structure.

**Figure 5:**
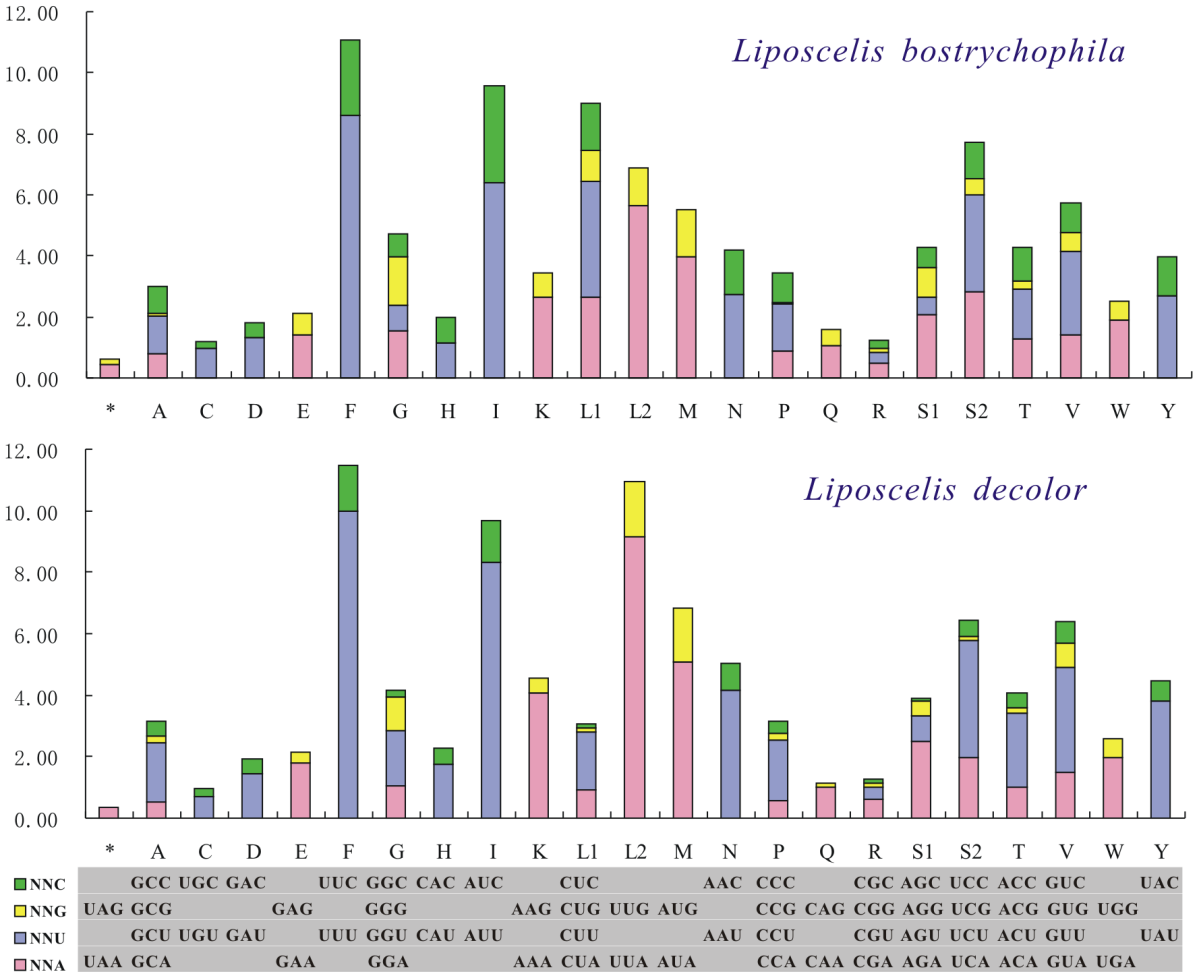
Relative synonymous codon usage (RSCU) for protein coding genes of *Liposcelis decolor* and *L. bostrychophila*. Abbreviations of tRNA genes are according to the single letter according to the IPUC-IUB one-letter amino acid codes.

The A+T content of the *L. decolor* mt genome is 75.23%. This is typical of psocodean insects but is higher than that of *L. bostrychophila*, 68.63% (Figure S2 and Table S5). The higher A+T content of *L. decolor* is present in all regions, both genes and non-coding regions (Table S5). The difference of A+T content between the two booklice is reflected further in the codon usage: the relative synonymous codon usages (RSCU) of the two booklice showed that *L. decolor* used more NNA and NNT codon than *L. bostrychophila* (Figure 5). The nucleotide composition of mt genome is usually conserved within a genus; however, it varies between *L. decolor* and *L. bostrychophila*. This variation may be related to mt genome fragmentation, because all of the psocodean fragmented mt genomes have a lower A+T content (Figure S2).

### Mitochondrial gene rearrangement in *L. decolor*

**Figure 6:**
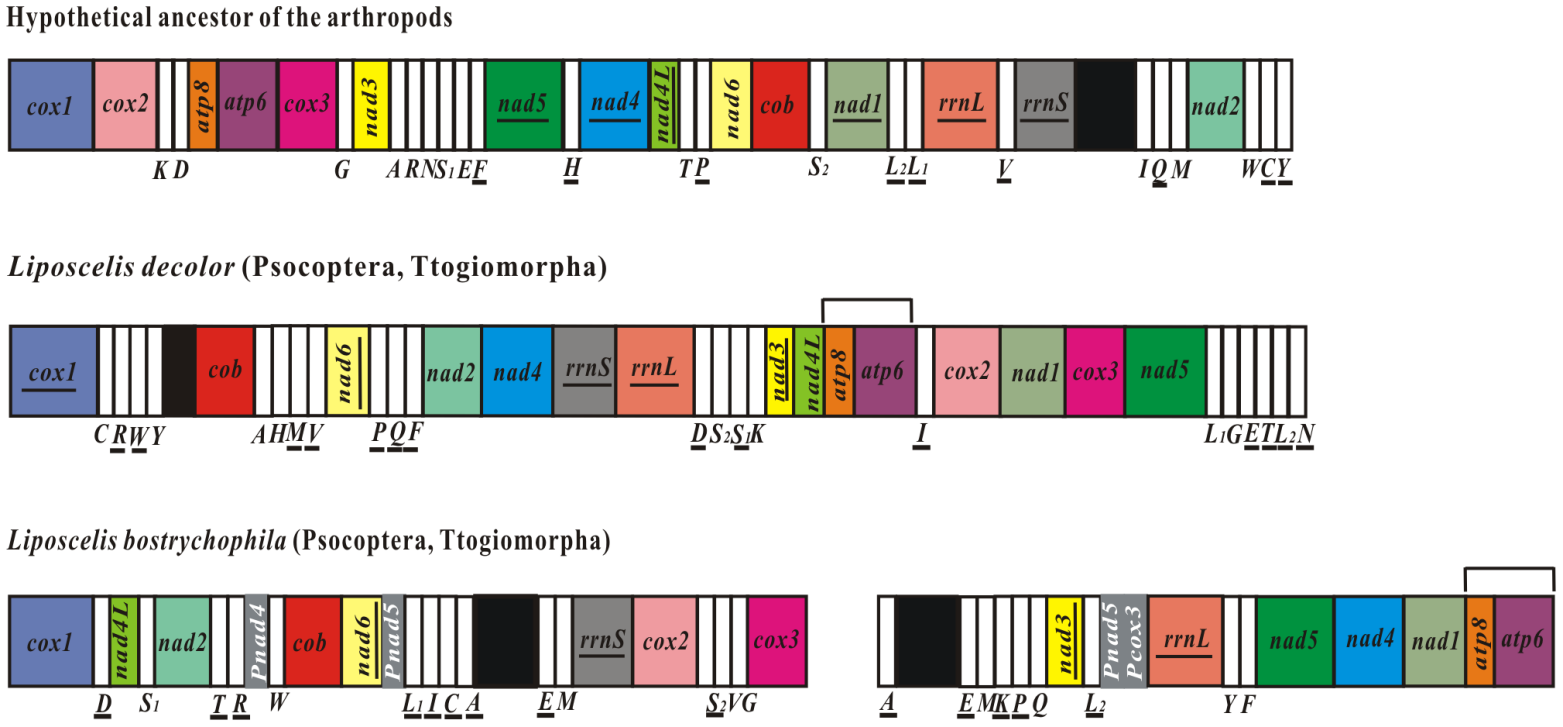
Arrangement of mitochondrial genes in *Liposcelis* and the hypothetical ancestor of the arthropods. Circular genomes have been arbitrarily linearized for ease of comparison. Gene names are the standard abbreviations used in the present study. tRNA genes are designated by the single letter according to the IPUC-IUB one-letter amino acid codes. Genes which are underlined are encoded on the opposite strand to the majority of genes in that particular genome. Black, gray, and white boxes represent putative control regions, pseudogenes, and transfer RNA genes, respectively. The boxes in 15 colors represent 13 protein coding genes and 2 ribosomal RNA genes. Shared gene-boundaries are labeled with square “brackets” above each genome.

The mt gene arrangement in *L. decolor* differs substantially from that of the hypothetical ancestor of insects and from that of the booklouse, *L. bostrychophila* (Figure 6). With the only exception of *atp8-atp6*, there is no gene boundary or gene block shared between *L. decolor* and *L. bostrychophila*, even though these two booklice belong to the same genus. *Atp8*-*atp6*, is a highly conserved ancestral gene boundary for animals and is assumed to be constrained by the function of a bicistronic *atp8*-*atp6* transcript [46]. The mt gene arrangement is usually conserved within the same genus. For example, the two *Pediculus* species of human lice, both have extensively fragmented mt genomes but have the same mt gene arrangement [7]. Furthermore, *Coloceras* sp. and *Campanulotes bidentatus*, also have the same gene arrangement, except for a difference in the location of their *trnQ*. The extent of the variation in the mt gene arrangement between the two species of *Liposcelis* booklice is unprecedented for animals within the same genus, indicating that gene rearrangement occurred frequently after these two booklice diverged from each other. The genus *Liposcelis* is divided into four groups (A, B, C, D) phylogenetically [25], [26]; *L. decolor* and *L. bostrychophila* are in different groups, C and D respectively, although they often co-occur in a wide range of stored products in the same ecosystems. Previous studies have revealed that substantial variation exists among the *Liposcelis* groups at both morphological and molecular levels [25], . Whether or not the multipartite mt genome observed in *L. bostrychophila* occurred only in species of group D remains to be investigated.

### Phylogenetic relationship of *L. decolor* to other species in the Psocodea

**Figure 7:**
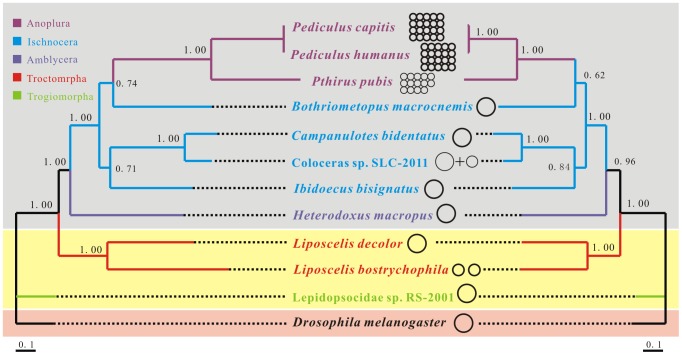
Phylogeny of the Psocodea inferred with mitochondrial genome sequences. Numbers above the left branches show Bayesian posterior probability for the phylogenies from nucleotide sequences, the right from amino acid sequences. Only support above 50% is shown. The insects belong to Phthiraptera, Psocoptera, and Diptera are shown in gray, yellow and pink background respectively.

Bayesian inference was used to determine phylogenetic relationships among 11 species of Psocodea from the orders Psocoptera and Phthiraptera, with nucleotide and deducted amino acid sequences of mt genomes (Figure 7 and Figure S3). Although *L. decolor* and *L. bostrychophila* differ in both mt genome organization and mt gene arrangement, these two booklice are more closely related to each other than to other species in the Psocodea. The two *Liposcelis* species formed a sister clade to the parasitic lice (order Phthiraptera). This was also indicated by previous studies [3], [30], [47]. The close relationships between the two booklice, and between the booklice and the parasitic lice are strongly supported by our analyses. Our results indicate that the bipartite mt genome organization observed in *L. bostrychophila* likely evolved recently *L. decolor* and *L. bostrychophila* split from their most recent common ancestor. Furthermore, from the phylogenies of the Psocodea constructed in the present study and a number of previous studies [7], [9], it can be inferred that the bipartite mt genome organization in *L. bostrychophila* evolved independently from the much more fragmented mt genomes observed in the blood-sucking lice (suborder Anoplura).

## Supporting Information

Figure S1 **Size of mitochondrial protein-coding genes and rRNA genes of the Psocodea.** Lower horizontal bar, non-outlier smallest observation; lower edge of rectangle, 25 percentile; the central horizontal bar, median; upper edge of rectangle, 75 percentile; upper horizontal bar, non-outlier largest observation; small circle, outlier. Species are abbreviated as following: *Ld*, *Liposcelis decolor*; *Lb*, *Liposcelis bostrychophila*; *Ls*, Lepidopsocidae sp. RS-2001; *Bm*, *Bothriometopus macrocnemis*; *Ac*, *Anaticola crassicornis*.
(TIF)

Figure S2 **A+T contents of the mitochondrial genomes of the Psocodea.** The checked bars designate the fragmented genomes. The mt genomes sequenced incompletely are marked by asterisks. Species are abbreviated as following: *Ld*, *Liposcelis decolor*; *Lb*, *Liposcelis bostrychophila*; *Ls*, Lepidopsocidae sp. RS-2001; *Bm*, *Bothriometopus macrocnemis*; *Cb*, *Campanulotes bidentatus*; *Cs*: *Coloceras* sp. SLC-2011; *Hm*, *Heterodoxus macropus*; *Ib*, *Ibidoecus bisignatus*; *Pc*, *Pediculus capitis*; *Ph*, *Pediculus humanus*; *Pp*, *Pthirus pubis*; *Ac*, *Anaticola crassicornis*; *Dm*, *Damalinia meyeri*; *Ps*, *Philopterus* sp. SLC-2011; *Qs*, *Quadraceps* sp. SLC-2011; *Bp*, *Brachionus plicatilis*.
(TIF)

Figure S3 **Alignments of mitochondrial gene sequences used for phylogenetic analyses.** A. Nucleotide sequence alignment (gene order of this alignment: *cox1*-*3*, *atp6*, *cob*, *nad1*-*3*, *nad5*-*6*, *rrnS* and *rrnL*; sequences of *nad3* and *nad6* just with codon positions 1 and 2); B. Amino acid sequence alignment (gene order of this alignment: *cox1*-*3*, *atp6*, *cob*, *nad1*-*3* and *nad5*-*6*). Species are abbreviated as following: Ld, *Liposcelis decolor*; Lb, *Liposcelis bostrychophila*; Ls, Lepidopsocidae sp. RS-2001; Bm, *Bothriometopus macrocnemis*; Cb, *Campanulotes bidentatus*; Cs: *Coloceras* sp. SLC-2011; Hm, *Heterodoxus macropus*; Ib, *Ibidoecus bisignatus*; Pc, *Pediculus capitis*; Ph, *Pediculus humanus*; Pp, *Pthirus pubis*; Dm, *Drosophila melanogaster.*
(DOC)

Table S1 **PCR primers used for the amplification of the mitochondrial genome of** ***Liposcelis decolor*** **.**
(DOC)

Table S2 **GenBank accession numbers of all the insects mentioned in this study.**
(DOC)

Table S3 **Summary of the mitochondrial genome of** ***Liposcelis decolor*** **.** ^a^Genes located in the different strand from that of *cox1* are underlined. ^b^inc  =  intergenic nucleotides, indicating gap nucleotides (positive value) and overlapping nucleotides (negative value) of two adjacent genes. ^c^AT-skew  =  (A−T)/(A+T), GC-skew  =  (G−C)/(G+C). ^d^CR  =  control region (putative).
(DOC)

Table S4 **Start and stop codons of mitochondrial protein-coding genes of the Psocodea.** Species are abbreviated as follows: *Ld*, *Liposcelis decolor*; *Lb*, *Liposcelis bostrychophila*; *Ls*, Lepidopsocidae sp. RS-2001; *Bm*, *Bothriometopus macrocnemis*; *Cb*, *Campanulotes bidentatus*; *Cs*, *Coloceras* sp. SLC-2011; *Hm*, *Heterodoxus macropus*; *Ib*, *Ibidoecus bisignatus*; *Pc*, *Pediculus capitis*; *Ph*, *Pediculus humanus*; *Pp*, *Pthirus pubis*; *Ac*, *Anaticola crassicornis*; *Dm*, *Damalinia meyeri*; *Ps*, *Philopterus* sp. SLC-2011.
(DOC)

Table S5 **Nucleotide compositions of the mitochondrial genomes of** ***Liposcelis decolor*** **and** ***L. bostrychophila*** **.**
(DOC)

## References

[1] Boore JL (1999) Animal mitochondrial genomes. Nucleic Acids Research 27: 1767–1780. 10101183 10.1093/nar/27.8.1767 PMC148383

[2] Sheffield NC, Song H, Cameron L, Whiting MF (2008) A comparative analysis of mitochondrial genomes in Coleoptera (Arthropoda: Insecta) and genome descriptions of six new beetles. Molecular Biology and Evolution 25: 2499–2509. 18779259 10.1093/molbev/msn198 PMC2568038

[3] Wei DD, Shao R, Yuan ML, Dou W, Barker SC, et al (2012) The multipartite mitochondrial genome of *Liposcelis bostrychophila*: insights into the evolution of mitochondrial genomes in bilateral animals. PLoS One 7: e33973. 22479490 10.1371/journal.pone.0033973 PMC3316519

[4] Satoh TP, Sato Y, Masuyama N, Miya M, Nishida M (2010) Transfer RNA gene arrangement and codon usage in vertebrate mitochondrial genomes: a new insight into gene order conservation. BMC Genomics 11: 479. 20723209 10.1186/1471-2164-11-479 PMC2996975

[5] Covacin C, Shao R, Cameron S, Barker SC (2006) Extraordinary number of gene rearrangements in the mitochondrial genomes of lice (Phthiraptera: Insecta). Insect Molecular Biology 15: 63–68. 16469069 10.1111/j.1365-2583.2005.00608.x

[6] Shao R, Campbell NJH, Barker SC (2001) Numerous gene rearrangements in the mitochondrial genome of the wallaby louse, *Heterodoxus macropus* (Phthiraptera). Molecular Biology and Evolution 18: 858–865. 11319269 10.1093/oxfordjournals.molbev.a003867

[7] Shao R, Zhu XQ, Barker SC, Herd K (2012) Evolution of extensively fragmented mitochondrial genomes in the lice of humans. Genome Biology and Evolution 4: 1088–1101. 23042553 10.1093/gbe/evs088 PMC3514963

[8] Cameron SL, Yoshizawa K, Mizukoshi A, Whiting MF, Johnson KP (2011) Mitochondrial genome deletions and minicircles are common in lice (Insecta: Phthiraptera). BMC Genomics 12: 394. 21813020 10.1186/1471-2164-12-394 PMC3199782

[9] Shao RF, Kirkness EF, Barker SC (2009) The single mitochondrial chromosome typical of animals has evolved into 18 minichromosomes in the human body louse, *Pediculus humanus* . Genome Research 19: 904–912. 19336451 10.1101/gr.083188.108 PMC2675979

[10] Suga K, Welch DBM, Tanaka Y, Sakakura Y, Hagiwarak A (2008) Two circular chromosomes of unequal copy number make up the mitochondrial genome of the rotifer *Brachionus plicatilis* . Molecular Biology and Evolution 25: 1129–1137. 18326862 10.1093/molbev/msn058

[11] Gibson T, Blok VC, Phillips MS, Hong G, Kumarasinghe D, et al (2007) The mitochondrial subgenomes of the nematode *Globodera pallida* are mosaics: evidence of recombination in an animal mitochondrial genome. Journal of Molecular Evolution 64: 463–471. 17479345 10.1007/s00239-006-0187-7

[12] Gibson T, Blok VC, Dowton M (2007) Sequence and characterization of six mitochondrial subgenomes from *Globodera rostochiensis*: multipartite structure is conserved among close nematode relatives. Journal of Molecular Evolution 65: 308–315. 17674076 10.1007/s00239-007-9007-y

[13] Watanabe KI, Bessho Y, Kawasaki M, Hori H (1999) Mitochondrial genes are found on minicircle DNA molecules in the mesozoan animal *Dicyema* . Journal of Molecular Biology 286: 645–650. 10024439 10.1006/jmbi.1998.2523

[14] Liu YG, Kurokawa T, Sekino M, Tanabe T, Watanabe K (2013) Complete mitochondrial DNA sequence of the ark shell *Scapharca broughtonii*: an ultra-large metazoan mitochondrial genome. Comparative Biochemistry and Physiology D-Genomics & Proteomics 8: 72–81. 10.1016/j.cbd.2012.12.003 23291309

[15] Liu GH, Wang Y, Song HQ, Li MW, Ai L, et al (2013) Characterization of the complete mitochondrial genome of *Spirocerca lupi*: sequence, gene organization and phylogenetic implications. Parasites & Vectors 6: 45. 23433345 10.1186/1756-3305-6-45 PMC3606334

[16] Kayal E, Bentlage B, Collins AG, Kayal M, Pirro S, et al (2012) Evolution of linear mitochondrial genomes in medusozoan cnidarians. Genome Biology and Evolution 4: 1–12. 22113796 10.1093/gbe/evr123 PMC3267393

[17] Nardi F, Spinsanti G, Boore JL, Carapelli A, Dallai R, et al (2003) Hexapod origins: monophyletic or paraphyletic? Science 299: 1887–1889. 12649480 10.1126/science.1078607

[18] Li H, Liu H, Shi AM, Stys P, Zhou XG, et al (2012) The complete mitochondrial genome and novel gene arrangement of the unique-headed bug *Stenopirates* sp. (Hemiptera: Enicocephalidae). PLoS One 7: e29419. 22235294 10.1371/journal.pone.0029419 PMC3250431

[19] Yuan ML, Wei DD, Wang BJ, Dou W, Wang JJ (2010) The complete mitochondrial genome of the citrus red mite *Panonychus citri* (Acari: Tetranychidae): high genome rearrangement and extremely truncated tRNAs. BMC Genomics 11: 597. 20969792 10.1186/1471-2164-11-597 PMC3091742

[20] Wei SJ, Shi M, Sharkey MJ, van Achterberg C, Chen XX (2010) Comparative mitogenomics of Braconidae (Insecta: Hymenoptera) and the phylogenetic utility of mitochondrial genomes with special reference to Holometabolous insects. BMC Genomics 11: 371. 20537196 10.1186/1471-2164-11-371 PMC2890569

[21] Yan D, Tang Y, Xue X, Wang M, Liu F, et al (2012) The complete mitochondrial genome sequence of the western flower thrips *Frankliniella occidentalis* (Thysanoptera: Thripidae) contains triplicate putative control regions. Gene 506: 117–124. 22750320 10.1016/j.gene.2012.06.022

[22] Boore JL, Brown WM (1998) Big trees from little genomes: mitochondrial gene order as a phylogenetic tool. Current Opinion in Genetics & Development 8: 668–674. 9914213 10.1016/s0959-437x(98)80035-x

[23] Athanassiou CG, Phillips TW, Aikins MJ, Hasan MM, Throne JE (2012) Effectiveness of sulfuryl fluoride for xontrol of sifferent life stages of stored-product psocids (Psocoptera). Journal of Economic Entomology 105: 282–287. 22420281 10.1603/ec11209

[24] Athanassiou CG, Arthur FH, Throne JE (2010) Efficacy of methoprene for control of five species of psocids (Psocoptera) on wheat, rice, and maize. Journal of Food Protection 73: 2244–2249. 21219743 10.4315/0362-028x-73.12.2244

[25] Wei DD, Yuan ML, Wang ZY, Wang D, Wang BJ, et al (2011) Sequence analysis of the ribosomal internal transcribed spacers region in psocids (Psocoptera: Liposcelididae) for phylogenetic inference and species discrimination. Journal of Economic Entomology 104: 1720–1729. 22066203 10.1603/ec11177

[26] Lienhard C (1990) Revision of the western palaearctic species of *Liposcelis* Motschulsky (Psocoptera: Liposcelididae) Zoologische Jahrbücher, Abteilung für Systematik, Ökologie und Geographie der Tiere. 117: 117–174.

[27] Cheng WX, Dou W, Chai YX, Wang JJ (2007) Comparison of biochemical and toxicological characterizations of glutathione S-transferases and superoxide dismutase between *Liposcelis bostrychophila* Badonnel and *L. entomophila* (Enderlein) (Psocoptera: Liposcelididae). Pesticide Biochemistry and Physiology 89: 151–157.

[28] Li FS (2002) Psocoptera Of China. Beijing: Science Press pp: 77–79.

[29] Hu F, Zhang GN, Wang JJ (2009) Antennal sensillae of five stored-product psocids pests (Psocoptera: Liposcelididae). Micron 40: 628–634. 19269834 10.1016/j.micron.2009.02.006

[30] Lyal CHC (1985) Phylogeny and classification of the Psocodea, with particular reference to the lice (Psocodea: Phthiraptera). Systematic Entomology 10: 145–165.

[31] Yang QQ, Zhao S, Kucerova Z, Stejskal V, Opit G, et al (2013) Validation of the 16S rDNA and COI DNA barcoding technique for rapid molecular identification of stored product psocids (Insecta: Psocodea: Liposcelididae). Journal of Economic Entomology 106: 419–425. 23448059 10.1603/ec12163

[32] Simon C, Frati F, Beckenbach A, Crespi B, Liu H, et al (1994) Evolution, weighting, and phylogenetic utility of mitochondrial gene-sequences and a compilation of conserved polymerase chain-reaction primers. Annals of the Entomological Society of America 87: 651–701.

[33] Laslett D, Canback B (2008) ARWEN: a program to detect tRNA genes in metazoan mitochondrial nucleotide sequences. Bioinformatics 24: 172–175. 18033792 10.1093/bioinformatics/btm573

[34] Lowe TM, Eddy SR (1997) tRNAscan-SE: a program for improved detection of transfer RNA genes in genomic sequence. Nucleic Acids Research 25: 955–964. 9023104 10.1093/nar/25.5.955 PMC146525

[35] Zuker M (2003) Mfold web server for nucleic acid folding and hybridization prediction. Nucleic Acids Research 31: 3406–3415. 12824337 10.1093/nar/gkg595 PMC169194

[36] Xia X, Xie Z (2001) DAMBE: software package for data analysis in molecular biology and evolution. Journal of Heredity 92: 371–373. 11535656 10.1093/jhered/92.4.371

[37] D'Onorio de Meo P, D'Antonio M, Griggio F, Lupi R, Borsani M, et al (2012) MitoZoa 2.0: a database resource and search tools for comparative and evolutionary analyses of mitochondrial genomes in Metazoa. Nucleic Acids Research 40: D1168–1172. 22123747 10.1093/nar/gkr1144 PMC3245153

[38] Katoh K, Misawa K, Kuma K, Miyata T (2002) MAFFT: a novel method for rapid multiple sequence alignment based on fast Fourier transform. Nucleic Acids Research 30: 3059–3066. 12136088 10.1093/nar/gkf436 PMC135756

[39] Suyama M, Torrents D, Bork P (2006) PAL2NAL: robust conversion of protein sequence alignments into the corresponding codon alignments. Nucleic Acids Research 34: W609–W612. 16845082 10.1093/nar/gkl315 PMC1538804

[40] Penn O, Privman E, Ashkenazy H, Landan G, Graur D, et al (2010) GUIDANCE: a web server for assessing alignment confidence scores. Nucleic Acids Research 38: W23–W28. 20497997 10.1093/nar/gkq443 PMC2896199

[41] Xia X, Lemey P (2009) Assessing substitution saturation with DAMBE Phylogenetic Handbook: A Practical Approach to Phylogenetic Analysis and Hypothesis Testing, 2nd Edition: 615–630.

[42] Posada D (2008) jModelTest: phylogenetic model averaging. Molecular Biology and Evolution 25: 1253–1256. 18397919 10.1093/molbev/msn083

[43] Darriba D, Taboada GL, Doallo R, Posada D (2011) ProtTest 3: fast selection of best-fit models of protein evolution. Bioinformatics 27: 1164–1165. 21335321 10.1093/bioinformatics/btr088 PMC5215816

[44] Ronquist F, Huelsenbeck JP (2003) MrBayes 3: bayesian phylogenetic inference under mixed models. Bioinformatics 19: 1572–1574. 12912839 10.1093/bioinformatics/btg180

[45] Huelsenbeck JP, Ronquist F, Nielsen R, Bollback JP (2001) Bayesian inference of phylogeny and its impact on evolutionary biology. Science 294: 2310–2314. 11743192 10.1126/science.1065889

[46] Simon M, Faye G (1984) Organization and processing of the mitochondrial oxi3/oli2 multigenic transcript in yeast. Mol Gen Genet 196: 266–274. 6387398 10.1007/BF00328059

[47] Grimaldi D, Engel MS (2006) Fossil Liposcelididae and the lice ages (Insecta: Psocodea). Proc Biol Sci 273: 625–633. 16537135 10.1098/rspb.2005.3337 PMC1560062

