# Supplementary figures and images for: The Complete Mitochondrial Genome of the Booklouse, Liposcelis decolor: Insights into Gene Arrangement and Genome Organization within the Genus Liposcelis
Source: PLoS One. 2014 Mar 17;9(3):e91902. doi: 10.1371/journal.pone.0091902 (PMC3956861; doi:10.1371/journal.pone.0091902)

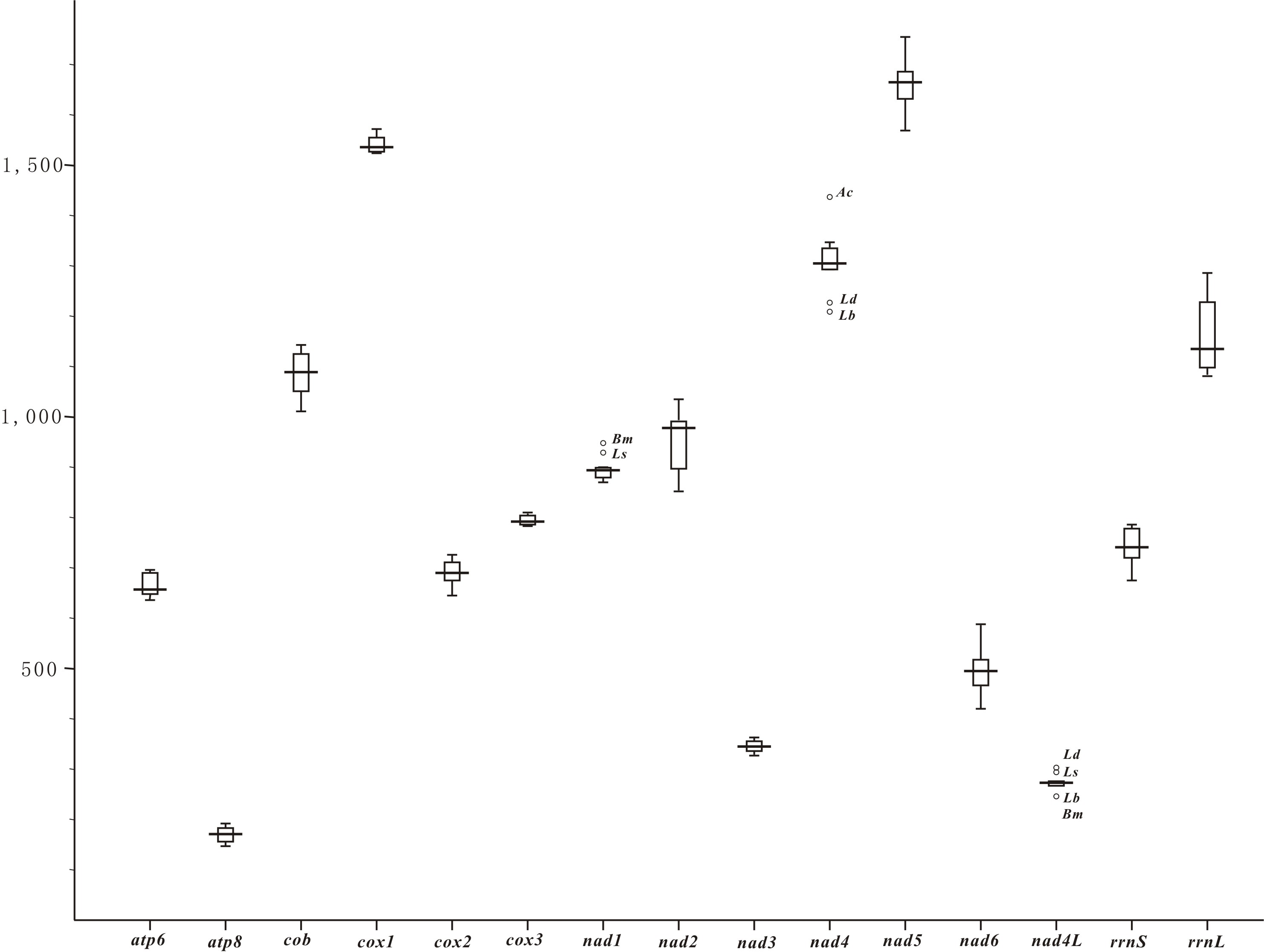

Supplement: Figure S1 — Size of mitochondrial protein-coding genes and rRNA genes of the Psocodea. Lower horizontal bar, non-outlier smallest observation; lower edge of rectangle, 25 percentile; the central horizontal bar, median; upper edge of rectangle, 75 percentile; upper horizontal bar, non-outlier largest observation; small circle, outlier. Species are abbreviated as following: Ld, Liposcelis decolor; Lb, Liposcelis bostrychophila; Ls, Lepidopsocidae sp. RS-2001; Bm, Bothriometopus macrocnemis; Ac, Anaticola crassicornis. (TIF) [file pone.0091902.s001.tif]

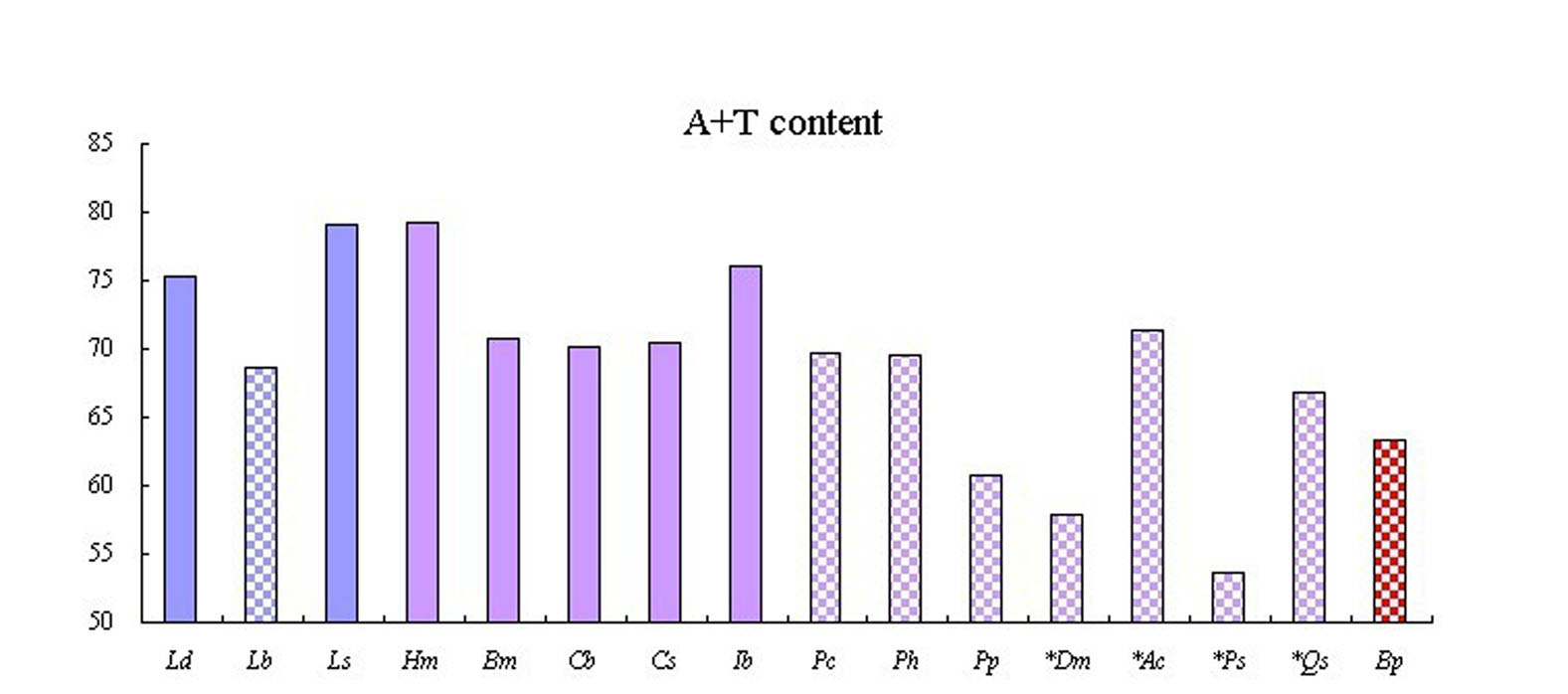

Supplement: Figure S2 — A+T contents of the mitochondrial genomes of the Psocodea. The checked bars designate the fragmented genomes. The mt genomes sequenced incompletely are marked by asterisks. Species are abbreviated as following: Ld, Liposcelis decolor; Lb, Liposcelis bostrychophila; Ls, Lepidopsocidae sp. RS-2001; Bm, Bothriometopus macrocnemis; Cb, Campanulotes bidentatus; Cs: Coloceras sp. SLC-2011; Hm, Heterodoxus macropus; Ib, Ibidoecus bisignatus; Pc, Pediculus capitis; Ph, Pediculus humanus; Pp, Pthirus pubis; Ac, Anaticola crassicornis; Dm, Damalinia meyeri; Ps, Philopterus sp. SLC-2011; Qs, Quadraceps sp. SLC-2011; Bp, Brachionus plicatilis. (TIF) [file pone.0091902.s002.tif]
