# Supplementary material for: The Complete Mitochondrial Genome of the Booklouse, Liposcelis decolor: Insights into Gene Arrangement and Genome Organization within the Genus Liposcelis
Source: PLoS One. 2014 Mar 17;9(3):e91902. doi: 10.1371/journal.pone.0091902 (PMC3956861; doi:10.1371/journal.pone.0091902)
Supplement: Figure S3 — Alignments of mitochondrial gene sequences used for phylogenetic analyses. A. Nucleotide sequence alignment (gene order of this alignment: cox1-3, atp6, cob, nad1-3, nad5-6, rrnS and rrnL; sequences of nad3 and nad6 just with codon positions 1 and 2); B. Amino acid sequence alignment (gene order of this alignment: cox1-3, atp6, cob, nad1-3 and nad5-6). Species are abbreviated as following: Ld, Liposcelis decolor; Lb, Liposcelis bostrychophila; Ls, Lepidopsocidae sp. RS-2001; Bm, Bothriometopus macrocnemis; Cb, Campanulotes bidentatus; Cs: Coloceras sp. SLC-2011; Hm, Heterodoxus macropus; Ib, Ibidoecus bisignatus; Pc, Pediculus capitis; Ph, Pediculus humanus; Pp, Pthirus pubis; Dm, Drosophila melanogaster. (DOC) [file pone.0091902.s003.doc]

A

Ld ATCAGAACCA ACCACAAAGA AATTGGAAAT TTATATTTCC TATTTGGGGT TTGGTCAGGA GTAGTAGGTT TAAGTATATC TATTCTTATG CGTTTTGAAG

Lb TTATCGACAA ATCATAAGGA TATCGGATCG CTATATTTTA TATTTGGGGT TTGATCGGGT CTATTAGGCC TAAGACTTAG GCTTTTGATA CGAGTAGAAA

Ls TTTTCTACAA ATCATAAAGA TATTGGTACT TTATATTTTC TATTAGGAAT TTGAGCAGGA ATAGTTGGAA CTAGTATAAG AATTTTAATT CGATTTGAAG

Bm TTTTCAACTA ATCACAAAGA TATTGGAATT TTATATATAA TTTTTGGGGT GTGATCAGGA TTGATTGGTT TTGGGTTAAG AATAATCATC CGAATTCAAT

Cb TTTTCTTCTA ATCATAAGGA TATTGGTATG ATGTATTTAA TTTTTGGAAT GTGGAGAGGA TTATTAGGTT ATGGGATAAG GGTAGTAATT CGTACGGAGA

Cs TTTTCTTCTA ACCATAAGGA TATTGGGGTA ATATATTTAA TTTTTGGAAT GTGGAGAGGT TTGTTAGGAT ATGGGATAAG AGTAATTATT CGAACTGAAA

Hm TATTCTTCTA ATCACAAAAA TATTGGTATT TTATATATAA TTCTAGGAAG ATGATCTGGA CTATTAGGAT TTAGATTGAG AATAATAATT CGATTAGAAT

Ib TATTCGACTA ATCATAAAGA CATTGGGATA CTTTATTTGA TTTTTGGAAT CTGATCAGGA TTATTAGGTT ATAGTATAAG ACTGATTATT CGAATAGAAA

Pc TTTTCTACAA ATCATAAAGA TATTGGATTT TTATACTTAT GCTCTGGAGT TTGGTTTGGA CTTTTAGGCT TAAGGTTAAG GTTAATAATC CGGTTAGAAT

Ph TTTTCTACAA ATCATAAAGA TATTGGATTT TTATACTTAT GCTCTGGAGT TTGGTTTGGA CTTTTAGGCT TAAGGTTAAG GTTAATAATC CGGTTAGAAT

Pp TTTTCTACTA ATCACAAGGA TATTGGACTT TTGTATCTGC TTTCTGGAAT TTGATTTGGT TTAGTTGGCT TGTCAATGAG TCTAATTGTG CGTGTAGAAG

Dm TTTTCTACAA ATCATAAAGA TATTGGAACT TTATATTTTA TTTTTGGAGC TTGAGCTGGA ATAGTTGGAA CATCTTTAAG AATTTTAATT CGAGCTGAAG

Ld GTTCTTCTAT TTTTAATTGA TTGATTACTT CACATGCTTT TCTTATAATT TTTTTTATAG TCATGCCCAT ATTAATTGGG GGGTTTGCTA ATTGATTGGT

Lb GCTCTTCTGT GTTTAATAGA CTAATCACCT CACATGCTTT TCTAATAATT TTTTTTTTTA TTATACCTAT ATTAATTGGG GGCTTTTCTA ATTGGATAAT

Ls AAGATGATAT TTATAATGTT ATTGTAACAG CTCATGCATT TATTATAATT TTTTTTATAA TTATACCTAT TATAATTGGT GGATTTGGTA ATTGATTAAT

Bm TTGATGGTAT TTTTAATGTT GTTGTTACAA TTCATGCTTT TCTAATAATT TTCTTCATAG TTATACCTAT AATAATTGGT GGATTTGCAA ATTGGATAGT

Cb GAGATTCTAT TTTTAATGTG TTTGTTACGG CTCATGCTTT TTTAATAATT TTTTTTATAG TTATACCGAT TATAATTGGG GGTTTTGCAA ATTGATTAGT

Cs GAGATTCGAT TTTTAATGTT TTTGTAACAG CTCATGCTTT TTTAATAATT TTTTTTATAG TAATACCAAT TATAATTGGA GGATTTGCTA ATTGACTAGT

Hm TTAACCCAAT TTACAATGTT GTTGTTACAT CACATGCATT TTTAATAATT TTTTTCTTTA TTATACCATT TATAATTGGA GGTTTTGCTA ATTGATTAGT

Ib ATGATGGTAT CTACAACGTA ATCGTTACAT CTCATGCTTT TTTAATAATT TTTTTTATAA TTATACCAAT TATAATTGGA GGTTTTGCTA ATTGGTTAGT

Pc CTGATAGACT ATATAACGTA TTTGTTACTT CTCACGCTTT TGTAATGATT TTTTTTATAG TTATGCCTGT AATAATAGGC GGTTTTGCAA ATTGATTAGT

Ph CTGATAGACT ATATAACGTA TTTGTTACTT CTCACGCTTT TGTAATGATT TTTTTTATAG TTATGCCTGT AATAATAGGC GGTTTTGCAA ATTGATTAGT

Pp TTAATAGAAC TTATAACGTG TTTGTTACAT CTCACGCTTT TGTAATGATT TTTTTTATGG TTATGCCTGT TATGATAGGA GGTTTTGCTA ACTGGCTAGT

Dm GAGATGATAT TTATAATGTA ATTGTAACTG CACATGCTTT TATTATAATT TTTTTTATGG TTATACCTAT TATAATTGGT GGATTTGGAA ATTGATTAGT

Ld ACCTTTAATA CTAGGAGCTC CAGATATGGC TTTTCCTCGT TTAAATAATC TAAGATTCTG ACTTTTACCG CCTTCTTTGT TGTTATTATT ATCTTCAACT

Lb CCCACTTTTG ATTAGATCAC CTGATATAGC CTTCCCACGG TTGAATAATT TAAGATTTTG ATTTCTCCCC CCTTCTTTAC TTTTAATTTC ATTCAGAATA

Ls TCCTTTAATA TTAAGAGCAC CTGATATAGC ATTTCCTCGA ATAAATAATA TAAGTTTTTG ATTATTACCC CCTTCATTAA CTTTATTATT AATAAGTAGA

Bm ACCTATTATG TTAGGTGCTC CTGATATGGC TTTTCCTCGA ATAAATAATA TGAGATTTTG ACTTCTTCCT CCTTCATTGA TTTTACTTTT AATGGCTACA

Cb TCCTGTGATA GTAGGGGCTG TTGATATGAT TTTTCCTCGG ATGAATAATA TAAGGTTTTG GCTTCTTCCT CCGTCATTAG TTTTATTATT AATAAGGAGG

Cs TCCGGTAATG ATTGGAGCTG TGGATATAAT TTTTCCTCGA ATGAATAATA TGAGTTTTTG GTTACTTCCT CCTTCTTTAG CTTTATTATT AATTAGGAGT

Hm ACCTATTATA AATGGTAGGC CTGATATATC GTTCCCTCGA ATAAACAATA TAAGGTTTTG ATTATTACCT CCTTCTTTAA TCTTTATATT ATGTAGAATA

Ib TCCTTTAATA ATTGGCTCTC CAGATATAGC ATTTCCTCGA ATAAATAATA TTAGATTTTG ATTACTTATT CCTTCTTTAT TATTTTTATT AATAAGAATT

Pc TCCTTCAATA TTAGGGTCTC CAGATATAGC ATTTCCTCGT ATAAATAATA TGAGTTATTG ACTTCTCACA CCCTCTGGGA TTTTGCTTAT TAGTAGCTCA

Ph TCCTTCAATA TTAGGGTCTC CAGATATAGC ATTTCCTCGT ATAAATAATA TGAGTTATTG ACTTCTCACA CCCTCTGGGA TTTTGCTTAT TAGTAGCTCA

Pp TCCTTTATTT TTAGGTGCTC CTGACATGGC TTTCCCCCGG ATGAACAATA TAAGCTATTG GCTGATTATA CCCTCTGGGG TTCTGTTAAT TGCGAGTTCA

Dm GCCTTTAATA TTAGGTGCTC CTGATATAGC ATTCCCACGA ATAAATAATA TAAGATTTTG ACTACTACCT CCTGCTCTTT CTTTACTATT AGTAAGTAGA

Ld TTTATAGATC GAGGAGTGGG GACCGGGTGG ACTGTCTACC CCCCATTATC ATTCAGATCA GTCGATTTAG CTATTTTTTC TTTACATTTA GCAGGGGCAA

Lb ATCGTTGGAC CAGGCGCAGG AACCGGGTGG ACAGCCTACC CTCCTCTGTC AGCTATCGAA GTAGATCTAG TAATTTTTTC CCTACATTTG GCTGGAATTA

Ls ATAACAAATG TTGGTGCTGG AACCGGATGA ACAGTTTACC CTCCTTTATC AGCAGCAGTC GTTGATTTAG CAATTTTTTC TTTACATTTA GCTGGAATTA

Bm TGCATGGAAT CAGGTGTAGG ATCAGGATGG ACGTTATATC CTCCTCTATC TTCTATCTTA GTGGGATATT TAATTTTCTC CCTTCATTTA GCAGGTGTGA

Cb GTTATTGATA ATGGGGTAGG AACTGGGTGG ACGGTTTATC CTCCACTTTC AAGATTTGTT GTGGATTATG CTATTTTCTC TCTTCATTTA GCTGGGGTTA

Cs GTGATTGATA GAGGGGTGGG GACTGGGTGA ACTGTTTATC CTCCTCTCTC TAGGTTTACT GTAGATTTTG CTATTTTTTC TCTTCATTTA GCTGGAGTTA

Hm ATACTAGATG GAGGTTCTGG GACAGGTTGA ACTGTTTACC CTCCATTGTC TTCTTTAACT GTAGATATAT TAATTTTTTC TCTTCATCTT GCTGGGATTA

Ib TTTATAGGAG AAGGAACTGG AACAGGATGA ACCGTATACC CCCCCTTATC AAGTCAAGCA GTAGACATTT CAATTTTTTC TTTACACTTA GCAGGTTTAA

Pc TTTGTTCAAG GTGGTGTGGG TACTGGCTGG ACTGTTTATC CCCCTCTTAG GTCTCTAGAA GTTGATTTAG CTATTTTAAG TCTTCATTTA GCAGGAGTGA

Ph TTTGTTCAAG GTGGTGTGGG TACTGGCTGG ACTGTTTATC CCCCTCTTAG GTCTCTAGAA GTTGATTTAG CTATTTTAAG TCTTCATTTA GCAGGAGTGA

Pp ATAATCCAAG GTGGAACAGG TACTGGCTGG ACTATTTATC CTCCGCTAAG TCCTTTAGAA GTGGATTTTA CCATCTTTAG CCTCCATTTA GCTGGAGTAA

Dm ATAGTTGAAA ATGGAGCTGG AACAGGATGA ACTGTTTATC CACCTTTATC CGCTGGAATT GTTGATTTAG CTATTTTTTC TCTACATTTA GCAGGGATTT

Ld GTTCTATTTT AGGTGCTATT AATTTTATTA CTACTTTCTT TAATTTAAGT TTATATTCTT GGTCTGTGGC TATTACTGCC ATTTTATTGT TATTATCTTT

Lb GTTCCATCCT GGGAGCCATC AATTTTATCA CTACTTCAAT TAATTTACCT TTATTTAGAT GGTCTGTGTT AATCACAGCA TTTCTTTTGC TTCTTTCTCT

Ls GATCAATTTT GGGAGCAGTA AATTTTATTT CAACAATTAT TAATATACCA CTATTTGTAT GATCCGTTTT CTTAACAGCA ATTTTATTAT TATTATCTTT

Bm GATCAATTAT AGGAGCAATT AATTTTATTT CTACTATCCT TAATATGCCT CTATTCTGTT GGGCTGTGCT TATTACAGCC ATTCTTTTAC TTCTATCTCT

Cb GGTCAATTAT AGGGGCAATT AATTTTATCT GCACTATTTT AAATATGCCG TTGTTTTGTT GATCGGTTTT AATTACTGCA TTTCTTTTAC TTCTTTCTCT

Cs GTTCAATTAT AGGAGCAATT AATTTTATTT GTACAATTAT GAATATGCCA TTATTTTGCT GGTCAGTAAT AATTACTGCA TTTCTCTTAC TTCTTTCTCT

Hm GATCAATTAT AGGGGCTATC AATTTTATTA CTACTATTTT TAACATAAGA TTATTTAATT GATCAGTATT AATTACTGCT TTTTTATTAC TTTTATCTTT

Ib GTTCAATTTT GGGAGCTATT AATTTTATTT GTACTATTAT AAATATACCC TTATTTTGTT GATCAATTTT AATTACTGCT TTTTTATTAT TACTTTCCTT

Pc GTTCGATTTT AGGATCAGTA AATTTTATTA GAACTATTTT TAACATACCT TTATTTTGCT GGAGAGTGTT GGTAACAGCC TTTTTATTAT TACTGTCACT

Ph GTTCGATTTT AGGATCAGTA AATTTTATTA GAACTATTTT TAACATACCT TTATTTTGCT GGAGAGTGTT GGTAACAGCC TTTTTATTAT TACTGTCACT

Pp GTTCTATTTT AGGCTCAGTA AACTTTATTA GGACAATTTT AAATATACCT TTGTTCTGCT GGTCTGTCTT AATCACGGCC TTTTTGCTTC TACTCTCTTT

Dm CTTCAATTTT AGGAGCTGTA AATTTTATTA CAACTGTAAT TAATATACCT TTATTTGTTT GATCAGTAGT TATTACTGCT TTATTATTAT TATTATCACT

Ld ACCTGTCCTT GCGGGTGCTA TCACTATGTT ATTATTCGAT CGCAATTTAA ACACTTCTTT TTTTGAACCT TCAGGGGGCG GAGACCCTAT TCTTTATCAA

Lb TCCCGTTTTG GCTGGAGCAA TTACTATACT TTTATTTGAT CGTAATTTAA GAACTTCATT CTTTGATCCA TCTGGAGGGG GGGACCCAAT TCTCTTTCAA

Ls ACCTGTATTA GCAGGGGCAA TTACTATATT ATTAACAGAT CGAAATTTAA ATACCTCATT TTTTGATCCT GCAGGTGGAG GAGATCCTAT TCTTTATCAA

Bm TCCAGTTTTA GCTGGAGCTA TTACTATGCT TCTGTTAGAT CGAAGCTTAA ATACTTCCTT TTTTTCACCA GAATTAGGAG GTGATCCAAT TTTATATCAA

Cb TCCAGTATTG GCAGGAGCAA TTACTATGCT TTTGTTTGAT CGGAATATTA ATACTTCATT TTTTGATCCG TCGGGAGGAG GGGATCCTGT CTTGTATCAA

Cs TCCTGTATTA GCAGGAGCAA TTACAATACT TTTGTTTGAT CGGAATATTA ATACATCGTT TTTTGATCCT TCGGGAGGGG GGGATCCAGT ATTATATCAA

Hm ACCAGTATTA GCAGGTGCTA TTACAATATT ATTATTTGAT CGAAATTTTA ATTCAAGATT TTTTGATCCT ATTGGAGGAG GAGACCCTAT TTTATATCAA

Ib ACCGGTTCTT GCAGGAGCAA TTACTATGTT ACTTCTAGAT CGGAATATTA ATTGTTCTTT CTTTGATCCT ATGGGAGGAG GAGATCCAAT TTTATACCAA

Pc TCCAGTTTTA GCTGGAGCTA TTACAATGCT CTTAATAGAC CGTAATTTCA ATTGCTCATT TTTTGATCCT TTAGGGGGTG GTGATCCTGT TTTATACCAA

Ph TCCAGTTTTA GCTGGAGCTA TTACAATGCT CTTAATAGAC CGTAATTTCA ATTGCTCATT TTTTGATCCT TTAGGGGGTG GTGATCCTGT TTTATACCAA

Pp ACCTGTACTT GCGGGTGCTA TCACTATGCT ACTATTAGAT CGGAATTTTA ATTGTTCCTT TTTTGACCCT CTAGGTGGAG GTGATCCTGT TCTTTACCAG

Dm TCCAGTACTA GCAGGAGCTA TTACTATATT ATTAACAGAT CGAAATTTAA ATACATCATT TTTTGACCCA GCGGGAGGAG GAGATCCTAT TTTATATCAA

Ld CATTTATTTT GATTTTTTGG TCACCCAGAA GTTTATATTC TTATTCTTCC GGCTTTTGGT ATTATTTCAC ATATTGTCTC TAGAGAAAGA TATAAAGATG

Lb CATTTATTTT GATTTTTTGG ACACCCTGAA GTCTATATTT TAATTTTACC AGGATTTGGG CTAATCTCTC ACATCATCTC TCAAGAGAGA ATAAAAGATG

Ls CATTTATTTT GATTTTTTGG TCATCCAGAA GTTTACATTT TAATTTTACC AGGATTTGGT ATTATTTCTC ACGTTATTAG ACAAGAAAGA AAAAAAGAAA

Bm CATTTGTTTT GGTTTTTTGG ACACCCTGAA GTATATATTT TAATTATTCC CGGATTTGGT TTAATATCCC ACATTATCAA TGAGTGTAGG AAACCATCTG

Cb CATTTGTTTT GATTTTTTGG TCATCCGGAG GTATATATTT TAATTCTTCC TGGATTTGGT TTAATTTCTC ATATGTTAAG GGATAATAGT AAAATAGAGG

Cs CATTTGTTTT GGTTTTTTGG TCATCCGGAG GTATATATTT TAATTCTTCC CGGATTTGGA TTAATTTCTC ATATTCTAAG TGATAATAGA AAGATGGAAG

Hm CACTTATTTT GATTTTTTGG ACACCCTGAA GTTTATATTT TAATTTTACC AGGATTCGGT TTAATTTCTC ATATTATTGT TCAAGAAAGA AAATGTGAAA

Ib CATTTATTTT GATTTTTTGG ACATCCTGAA GTATATATTT TAATTCTTCC TGGTTTCGGT CTAATTTCTC ATATTATTTG CGAAGAAAGA AAAAAAGAGG

Pc CATTTATTTT GATTTTTTGG ACATCCTGAA GTTTATATTC TTATTCTTCC TGGATTTGGT CTTATCTCTC ATATGGTGGT AGATTGTTGT AAGAAAGAAG

Ph CATTTATTTT GATTTTTTGG ACATCCTGAA GTTTATATTC TTATTCTTCC TGGATTTGGT CTTATCTCTC ATATGGTGGT AGATTGTTGT AAGAAAGAAG

Pp CATCTTTTTT GGTTTTTTGG TCATCCTGAG GTTTATATCT TGATCTTACC TGGGTTTGGA TTGATCTCTC ATATAGTTGT TGACTTGAGA AAGAAAGAAG

Dm CATTTATTTT GATTTTTTGG TCACCCTGAA GTTTATATTT TAATTTTACC TGGATTTGGA ATAATTTCTC ATATTATTAG ACAAGAATCA AAAAAGGAAA

Ld TTTTTGGGGT AATGGGGATA ATTTATGCTA TGAGTGCTAT TGGGGTTTTA GGGTTTGTAG TTTGGGCCCA TCATATATTT ACAGTAGGGT TAGATGTAGA

Lb TATTTGGCAG ATTAGGCATG ATCTATGCCA TGCTCTCAAT CGGAGCTCTA GGTTTCATCG TATGAGCCCA CCACATATTC ACTGTGGGCA TGGATGTGGA

Ls CATTTGGAGT TTTAGGTATA ATTTATGCTA TAATAGCAAT TGGATTATTA GGTTTTGTAA TATGAGCACA TCACATATTT ACAGTAGGTA TAGATGTAGA

Bm CTTTTGGGAG ATTAGGAATA ATTTACGCTA TACTTACAAT CGGATTATTA GGATTTTTAG TTTGAGCTCA TCATATATTT ACAGTAGGTA TGGATATTGA

Cb TTTTTGGATC ATTAGGAATG ATTTATGCAA TAGTAGCAAT TGGAGTGTTA GGATTTATTG TTTGGGCTCA TCATATGTTT ACTGTTGGTT TGGATGTAGA

Cs TTTTTGGGTC TTTAGGAATG ATTTATGCAA TATTGGCTAT TGGGGTATTG GGTTTTATTG TTTGGGCTCA TCATATATTT ACTGTTGGAT TGGATGTGGA

Hm CTTTCGGAGT TTTAGGAATA ATTTATGCTA TATTATCTAT TGGAATTCTT GGATTTATTG TATGAGCTCA TCATATATTT ACTATTGGTA TGGATGTTGA

Ib TGTTTGGTTC TTTAGGAATA ATTTATGCCA TGTTATCTAT TGGTATTTTA GGATTTGTTG TATGGGCACA TCATATGTTT ACTGTAGGTA TAGATGTGGA

Pc TTTTTGGGTC ATTAGGAATG ATTTACGCAA TATCCGCTAT TGGGGCTTTA GGTTTTGTAG TTTGAGCACA TCACATGTTT ACAGTTGGAT TAGATGTGGA

Ph TTTTTGGGTC ATTAGGAATG ATTTACGCAA TATCCGCTAT TGGGGCTTTA GGTTTTGTAG TTTGAGCACA TCACATGTTT ACAGTTGGAT TAGATGTGGA

Pp TCTTTGGTTC ATTAGGAATA ATCTACGCTA TAGTATCGAT TGGTGTTCTA GGGTTTGTTG TTTGAGCCCA CCACATGTTC ACTGTTGGTC TAGATGTAGA

Dm CTTTTGGTTC TCTAGGAATA ATTTATGCTA TATTAGCTAT TGGATTATTA GGATTTATTG TATGAGCTCA TCATATATTT ACCGTTGGAA TAGATGTAGA

Ld TACTCGAGCT TATTTTACTT CTGCGACTAT AATTATTGCT GTTCCTACAG GAATCAAGGT TTTTAGATGA CTAGCTACGT TGTATGGATC ATACATCAAA

Lb TAGACGAGCA TATTTTACCT CTGCAACAAT AATTATCGCG ATCCCTACCG GAGTTAAAGT TTTCTCTTGG TTAACTACTG TTTATGGGAG AACAGTTACT

Ls TACTCGAGCT TATTTTACAT CTGCAACAAT AATTATTGCT ATTCCAACAG GTATTAAAAT TTTTAGTTGA TTAGCAACTT TACATGGGTC AAAAATATTT

Bm TAGACGAGCA TATTTTACAA GGGTAACTAT AGTGATTGCT GTTCCAACAG GAATTAAGGT TTTTAGATGG TTGGGAACAA TTTTTGGGAG AAAAATTAAT

Cb TAGGCGGGCA TACTTTACTT CGGCTACTAT AGTAATTGCT GTTCCTACTG GAGTAAAAGT GTTTAGATGA ATGGCTACTT TATTTGGAAG ACGAGTAAAA

Cs TAGTCGAGCA TATTTTACCT CAGCTACTAT AGTTATTGCT GTTCCTACAG GGGTAAAGGT TTTTAGATGA ATGGCTACAT TGTTTAGAAG GCGAGTAATG

Hm TACTCGGGCA TATTTTACTT CAGCGACTAT AATTATTGCA ATTCCTACTG GAATTAAAAT TTTCAGATGG TTATCTACTT TTTTTGGTAG AAAAATAAAA

Ib CAGACGAGCT TACTTCACCG GAGCAACTAT AATTATTGCA GTTCCTACTG GAATTAAAGT ATTTAGATGA ATATCCACCT TATTTGCAAG GAATATTAAT

Pc TAGACGGGCT TATTTTACTA GCGCTACTAT AACAATTGCA ATTCCAACGG GAGTGAAAGT CTTTAGGTGA TTAGGCACTT TGTTTGGCCC AAAATTAAAA

Ph TAGACGGGCT TATTTTACTA GCGCTACTAT AACAATTGCA ATTCCAACGG GAGTGAAAGT CTTTAGGTGA TTAGGCACTT TGTTTGGCCC AAAATTAAAA

Pp TAGACGTGCT TACTTCACTA GAGCTACTAT GACCATTGCT ATTCCAACGG GAGTAAAAGT CTTTAGCTGA TTAGGTACTC TATTTGGCCC TAAACTTCAC

Dm TACTCGAGCT TATTTTACCT CAGCTACTAT AATTATTGCA GTTCCTACTG GAATTAAAAT TTTTAGTTGA TTAGCTACTT TACATGGAAC TCAACTTTCT

Ld TTTTCTCCTT CTATAATTTG GGCTTGAGGT TTTATTTACT TATTTACTAT AGGAGGTTTA ACAGGGATTA TGTTATCAAA TTCTTGTATC GATATTGCTT

Lb CCTTCTTCTT CGACTTTATG AAGTCTAGGA TTTATTTACT TATTCACCAT CGGGGGTTTA ACTGGGATTA TCCTATCGAA TTCAAGAATT GATGTTATTC

Ls TTTTCTCCAT CATCTTTATG ATCTTTAGGA TTCGTATTTT TATTTACCAT TGGTGGTTTA ACAGGTGTTA TTTTAGCTAA TTCTTCTATC GATATTGCTC

Bm TGATCTCTCT CTTCTTTATG AAGAGTAGGG TTCATTTTTT TATTCACCTT GGGTGGACTC ACAGGTGTAG TATTAGCTAA TTCTTCAATT GATACTTTCA

Cb TGAAGTCCTT CGGAATTATG AGGAATTGGA TTTATTTTCT TGTTCACTGT TGGGGGTTTA ACTGGAGTAG TTCTTGCTAA TTCTTCTTTA GATATTATTC

Cs TGAAGTCCTT CAGAGTTGTG AAGGGTTGGA TTTATTTTTC TTTTTACTGT TGGGGGATTG ACTGGTGTTG TTTTAGCTAA TTCATCCTTA GACATCGTTC

Hm TTTAATTCTT CAGAATTATG AAGAATGGGT TTCGTTTTCT TATTTACTGT AGGTGGTTTA ACAGGGGTAG TTTTAGCAAA CTCTTCTATT GACATTGTTC

Ib TGGTCAGTAT CTTCATTATG AAGGTTAGGA TTTGTCTTTC TATTTACAAT CGGAGGTCTT ACGGGGGTAA TACTAGCTAA CTCATCAATT GATATTGCTC

Pc AGGAGAATTA GCTTGTTGTG ATCTTTAGGA TTTATTTTCC TTTTTACAAT TGGAGGTTTA ACAGGCATTG TTCTTTCTAA CTCATCCGTA GATGTTTCAC

Ph AGGAGAATTA GCTTGTTATG ATCTTTAGGA TTTATTTTCC TTTTTACAAT TGGAGGTTTA ACAGGCATTG TTCTTTCTAA CTCATCCGTA GATGTTTCAC

Pp TTAAGAGTGA GGTTGCAGTG GTCTCTGGGA TTCATCTTTT TATTTACTGT CGGGGGCCTG ACAGGGATTA TTCTATCTAA CTCGTCAGTT GACGTTCTTC

Dm TATTCTCCAG CTATTTTATG AGCTTTAGGA TTTGTTTTTT TATTTACAGT AGGAGGATTA ACAGGAGTTG TTTTAGCTAA TTCATCAGTA GATATTATTT

Ld TACATGATAC GTATTATGTT GTTGCCCATT TTCATTATGT TCTTTCTATA GGTGCTGTAT TCGCTATTTT TGGTGGATTA ATTTTCTGAT TTCCTTTATT

Lb TACATGATAG ATACTATGTT GTTGCCCACT TCCACTATGT TCTTTCCATG GGGGCTGTAT TTTCAATCTT TAGAGGATTG AATTTCTGAT TGCCTCTTTT

Ls TACACGATAC ATATTATGTA GTAGCACATT CCCATTATGT ACTATCTATA GGAGCTGTAT TTGCTATTAT AGCTGGGTTT ATTCAATGAT TTCCTCTTCT

Bm TACATGACAC ATACTATGTA GTTGCTCACT TCCGCTACGT CTTGTCTATA GGGGCAGTAT TTGCTATGTT TGCTAGATTA TTTCATTGAT TTCCTTTATT

Cb TTCATGATGC TTATTATGTG GTTGCTCATT TTCATTATGT TCTTTCTATA GGAGCTGTAT TTGCTGTATT TGGAGGATTT ATTCATTGGT TTCCAGTAAT

Cs TTCATGATAC ATATTATGTT GTTGCTCATT TTCACTATGT CCTTTCGATA GGAGCTGTAT TTGCTGTATT TGGAGGATTA GTTCACTGAT TTCCGGTTAT

Hm TTCATGACAC CTATTATGTT GTTGCCCATT TTCACTATGT GTTATCAATA GGAGCAGTAT TTGCGGTATT TTCAGCTTTT ACACATTGAT TTCCATTATT

Ib TTCACGATAC TTACTATGTT GTAGCTCATT TTCATTATGT TTTATCCATG GGTGCTATAG TAGCATTTAT GGCTAGGTTA TTCCATTGGT TCCCATTAAT

Pc TACATGACAC TTATTATGTA GTTGCTCACT TTCATTACGT TTTATCTATG GGTGCTGTAT TTGCTATTTT TGGCGCTTGA AACCACTGAT TCTCACTAGG

Ph TACATGACAC TTATTATGTA GTTGCTCACT TTCATTACGT TTTATCTATG GGTGCTGTAT TTGCTATTTT TGGCGCTTGA AACCACTGAT TCTCACTAGG

Pp TTCACGATAC CTACTATGTT GTAGCTCATT TCCACTATGT ATTGTCTATG GGTGCAGTGT TTGCTATCTT TGGTGCGTGG AACCATTGGT TTTCTTCTTT

Dm TACATGATAC TTATTATGTA GTAGCTCATT TTCATTATGT TTTATCTATA GGAGCTGTAT TTGCTATTAT AGCAGGTTTT ATTCACTGAT ACCCCTTATT

Ld TACAGGTTTA TTAATTAATC CTTTAAAATT AAAAGTTCAT TTTTTTCTTA CTTTTATTGG GGTGAATTTA ACTTTTTTTC CCCAACATTT TCTAGGGATA

Lb TCTGGGGGGG TCAGTTAATG AATTAAAAAA CAAAGTTCAC TTCTTCTTAA CTTTTATCGG AGTTAATCTC ACATTTTTCC CCCAACACTT TCTAGGTCTA

Ls TACTGGATTA ACTTTAAATA ATAATTGATT AAAAATTCAA TTTATAATTA TATTTATTGG AGTAAATATA ACTTTTTTTC CTCAACATTT TTTAGGTCTT

Bm TACAGGTTTA ACTCTTAATC AAAAGCTTAT AAAAATTCAC TTCTTTGTAA CTTTTATTGG AGTAAATTTA ACTTTCTTTC CTCAACACTT CTTAGGACTG

Cb TTTTGGGGTA AAAATAGAGT CTGTGTATTT AAAAGTTCAG TTTTTTTGTA CGTTTGTAGG GGTAAACCTA ACTTTCTTCC CTCAACATTT TCTTGGGTTA

Cs TTTTGGGGTA AAAATAAGAT CATCTTTTTT AAAGGTTCAA TTTTTCTCTG TGTTTGTGGG AGTAAATTTT ACTTTCTTTC CTCAACATTT TCTAGGATTG

Hm TTTTGGTGTA AAAATAAGAA ATGCTTTAAT AATTCTTCAT TTTTGAATTA CTTTTTTAGG AGTTAATTTA ACTTTTTTCC CTCAACATTT TTTAGGACTT

Ib TTTTGGAGTT TATCTTAATT CAAAATTTTT AAAAATTCAT TTTTTTGTAA CTTTTATTAG GGTAAACATA ATTTTTTTTC CTCAACATTT CTTAGGATTA

Pc GACTGGACTT AAACTTCGTA AGTCTTTTAT AAATGTTCAC TTTTGGTTAA GATTTGTGGG AGTGAATTTG ACTTTCTTTC CTCAGCACTT TCTCGGGTTA

Ph GACTGGACTT AAACTTCGTA AGTCTTTTAT AAATGTTCAC TTTTGGTTAA GATTTGTGGG AGTGAATTTG ACTTTCTTTC CTCAGCACTT TCTCGGGTTA

Pp AAGAGGCTGC TACCTTAACC CTAAAGTTAT ATCCACTCAT TTTTGAGTAA GGTTTATCGG GGTAAATTTA ACCTTTTTCC CTCAGCATTT CCTCGGTCTC

Dm TACTGGATTA ACGTTAAATA ATAAATGATT AAAAAGTCAT TTCATTATTA TATTTATTGG AGTTAATTTA ACATTTTTTC CTCAACATTT TTTAGGATTG

Ld ATATCTATAC CTCGTCGCTA TTCAGATTAC CCTGATTTTT TTTTATTTTT AAATTTGCTT TCTTCTTTAG GTTCTTGAAT TAGGTTAACT GCAGTTATTT

Lb TCTGGTCTCC CACGACGATA CTCGGACTAC CCAGACCACT ACACATATCT AAATCTGATT TCTTCGATTG GTTCATGAAT CAGAATAATT AGCATTATCT

Ls ATAGGAATAC CACGACGATA TAGAGATTAT CCAGATATTT ATACTTCATG AAATATAATT TCATCTTTGG GATCTACAAT TTCCTTAATT GGTATTATAT

Bm ATGGGTATAC CTCGACGTTA CGCGGACTAC CCAGATTTAT ATACTCCATG AAATTCAATT TCTTCAATTG GTAGATGTAT TTCTGTGGTC GGATTATCAA

Cb ATGGGAATAC CTCGGCGTTA TTCTGATTAT CCAGATATGT TTTATTCATG GAATTTTATT TCTTCAATAG GATCGCAAAT TACTTTGGTT GGAGTTTCAT

Cs ATGGGAATAC CCCGTCGGTA TTCTGATTAT CCCGATATAT TTTATTCTTG AAATGTAATT TCTTCTTTTG GTTCTCAAAT TTCAATGATT GGAGTTTCAT

Hm AGAGGTATGC CTCGACGTTA TATTTGTTAT CCCGATTTTT ATTATTCTTG AAATTTTTAT TCAAGAATTG GATCAATAAT TACTTCGGTA AGATTATTAA

Ib GCAGGAATAC CTCGTCGGTA TATAGATTAC CCAGATATAT TTAGTTCATG AAATGTAATT TCTTCTTTAG GTTCTACTTT ATCTATTATC AGTTTATTTA

Pc GCTGGGATGC CTCGACGTTA CTCAGACTAT CCTGACGTTT ACCTGAGGTG AAACAAAATT TCTTCAATAG GAAGGCTAAT TACTACTTTG GGTGTTGTAA

Ph GCTGGGATGC CTCGACGTTA CTCAGACTAT CCTGACGTTT ACCTGAGGTG AAACAAAATT TCTTCAATAG GAAGGCTAAT TACTACTTTG GGTGTTGTAA

Pp AGGGGGATAC CTCGACGTTA CTCAGATTAC CCGGATGCTT TCTACGGGTG GAATAAAATC TCCTCACTAG GAAGTATACT AACTTTTGTA GGAGTGCTAC

Dm GCTGGAATAC CTCGACGTTA TTCAGATTAC CCAGATGCTT ACACAACATG AAATATTGTA TCAACTATTG GATCAACTAT TTCATTATTA GGAATCTTAT

Ld TTTTTATTTT TATTATTCAT GAAAGATTTG TGAAATTGAA TATGGTCACC GAATGATTAG AAGGACAACC TATTAAATTT CAAGATTCAT GATCACCGTA

Lb GGTTAATTAC TCTTATTTTT GATGGTGTAA TAAAAAAAAA TTCTGTTATT GAATGAATCG AGGGGCAGCC ATTTAATTTA TTAGAAAGGA GGGGGCCTGT

Ls TTTTCATTTT TATTATATGA GAAAGTTTTA TTTCTAATCG AAAGCCTATT GAATGATTAC AAAAATATCC TTATAATTTA CAAGAAAGTG CTTCACCATT

Bm TATTGATTTA TGCTATTTAT GAAAGATTAA TTTCTCAGCG AAAAGCTATT GAGTGATTAT GAGGGTGTCC TTCTAAGTTT ATAGATCCAA TTTCTTTTTC

Cb TGTTTTTCTT TTGTTTAATT GAGGGGTTTT TTAGAAAACG AAGAGTGTTA GAGTGAATGA TTGGGTATCC TGTAGAATTG CAAGATAGGT GAGGTCCTTT

Cs TGTTTGTATT TTGTTTGATC GAAAGATTTT TGGCAAAACG TAAAGTGTTG GAGTGAATAA TTGGATTTCC TGTAAGTCTT CAGGATAGAT TTAGTCCTTT

Hm TATTTGTTTT CATAATTTTT TATAGATTTT TTGAAAATAA AAAATTATTA GAGTGAATGC TAGGAACTCC AATTGAACTT TCTGATGGAT GTTCATTAAT

Ib TAATGATGTT CTTAATTTTT GAAAGGCTAA TTTCAAAACG ACTGGTTGTG GAATGAGTAA ATGGATTTCC TACTACTTTT CAAGATAGAA ATTCTCCTTT

Pc TCTTTCTTTT AGCTCTTATA GAAAGGTTTT CTAATCCTCA AAAAATTGTA CCGCGTCTAA TGGGTATACC AAGAGTATTC CAAGATAGAA ATTCTCCTTT

Ph TCTTTCTTTT AGCTCTTATA GAAAGGTTTT CTAATCCTCA AAAAATTGTA CCGCGTCTAA TGGGTATACC AAGAGTATTC CAAGATAGAA ATTCTCCTTT

Pp TATTTCTCTA TGCTTCGTTT GACAGAGTTG CTAGAGCAAA TAAAGTACTG GTGGGTCTTG TTGGTAAGCC GTCAGATTTT CAGGATGGTA GATCTCCAGT

Dm TCTTTTTTTT TATTATTTGA GAAAGTTTAG TATCACAACG ACAAGTAATT GAATGATACC AAAATACTCC GACAAATTTA CAAGATAGAG CTTCTCCTTT

Ld TATGAGACAA TTAGCCAATT TTCATGAATA CGCGATAACT TATTTAATTT TTATTTTTTC TTTTATTACT GTTATTATAT ATATATCTTC AAGTATTAAT

Lb AATAGAACAA ATGAGGGAGT TTCATGATCA TGCTATAATG ATTCTTTTCC TCATCGTTTC TTTTTTAACT ATTGTTTTTT TAACTAATAA AAACCTTAAC

Ls AATAGAACAA CTAATTTTCT TTCATGATCA TTCATTATTA ATTATTACTA TAATTACTGT AATAGTCTCC TATATTATAT TTTTTAATTC AAATCGATTT

Bm TGGGGAAATT GTTCAATCAG TTCATGACCA TGTAATGATT ATTATTACTC TAATTGTTAT ATCAATTAGA TATGTATTTT TTTGTCGTTC GGAACGATTA

Cb AATAAGTCAT ATTTCTGGTT TTCATGATCA TGTAATAGTT GTCGTTTTAA TAATTTTAAC TGTGGTTGTA TATATTAATT TTTTTTTTCC TAGTCGGTTT

Cs AATAAATCAT ATTTCGATTT TTCATGATCA TGTAATAGTT GTAGTATTAA TAATTTTAAC TGTTGTAATT TATGTTAATT TTTTTTTTCC TGTTCGGTTT

Hm TATAGAAAAT ATAGTTGCAT TTCATGATTT CACTTTAATA ATTCTTTTAT TTATTACAAC AGTAGTACTA ATAATATTAA TAATTACTAA TAATCGATTT

Ib AATAATACAT ATTAACCATC TTCATGACCA TATTATGGTT GTTATTATTA TGATTATTTC TATTGTTATA TATGTATTAG TTATAAACCC TAATCGATTC

Pc AATAGTTTTT GTGTGTGATA CTTATGACCT TGTGTCTATT GTTTGTGTGG GGGTGATCTC CTTAGTAATG TACGTGGCTT TTTTTATAAA AAATTACTAT

Ph AATAGTTTTT GTGTGTGATA CTTATGACCT TGTGTCTATT GTTTGTGTGG GGGTGATCTC CTTAGTAATG TACGTGGCTT TTTTTATAAA AAATTACTAT

Pp TATAGCGTTT GTTTCTAATA CTTACGACCT TGTTTGTGTT GTTTGTGTCG GTGTTATCGC TTTAGTGGCT TATGTCTCTC TTTTGAACAA GAATTATTAC

Dm AATAGAACAA TTAATTTTTT TTCATGATCA TGCATTATTA ATTTTAGTAA TAATTACAGT ATTGGTGGGA TATTTAATAT TTTTTAATAA TAATCGATTT

Ld ACTGTAGACA ATGAAAAATT AGAATTATTG TGAACAATCA CCATTTTAAT TCTTTTAGCC GTCCCTTCTC TTAATGTTTT ATATTTCTTA GAAGAATCAT

Lb ATTTTGACAA GAGAAGTTCT TGAGATATTT TGGTCATCTC TGATCTTATT AATTCTTGCT ATTCCCTCTA TTCAAGTTCT GTTTATAATA GAAGAAGTTA

Ls TTATTAGAAA ATCAAACAAT TGAAATAATT TGAACTATTA TTGTATTAAT TTTCATTGCT TTACCTTCTT TACGACTTCT TTATTTATTA GACGAAACTA

Bm TTTCAATCCA GAGAAATTTT GGAAACAGTT TGAACTGTTT TTATTTTAGT TTTTGCAGCT ATTCCCTCTC TACATAGTTT ATATATTTTA GAAGAAGAAA

Cb ATAAAAAGAA GGGAGGGTTT AGAAACTTTG TGAACTATTC TTGTATTAGC TTCTTTAGCT GCTCCTTCTT TGATAACTTT GTATTTGTCT GATGAGTTAA

Cs TTTAAGAGGA GAGAGAGTTT GGAAACTATA TGAACTATTC TTGTTTTGGC TTCTTTAGCT GCTCCTTCAT TAATAACATT GTATTTATCT GATGAATTAA

Hm TTAATTTATA ATGAAGTATT AGAATTTATT TGAACAGTGA TTATTTTATT GATTATTGCA TTACCTTCTT TAAAAATTCT TTATTTAGTA GATGAATTAC

Ib TTTTTCGGTA GAGAAGTGTT GGAATTAATT TGAACTTTGG CTGTATTAGC AATTTTAGCT ATTCCATCAC TTCATATTTT ATACTTAATA GATGAATTAA

Pc TTTATAGGTC TTGAAAGATT GGAGATTGTT TGAGTTATCT TATCTTTAGC AGGGTTAATT TTACCGTCAC TTCATTGTTT ATACTTAATA GACGAGGTTC

Ph TTTATAGGTC TTGAAAGATT GGAGATTGTT TGAGTTATCT TATCTTTAGC AGGGTTAATT TTACCGTCAC TTCATTGTTT ATACTTAATA GACGAGGTTC

Pp TTTGTAGGCC TTGAGAGGTT AGAAACTATC TGGGTAATTT TAGCGCTAGC CTCTTTGGTC TTACCTTCTT TGCACTGTTT ATATTTGATG GATGAAATCT

Dm CTTTTACATG GACAACTTAT TGAAATAATT TGAACTATTT TAATTTTACT ATTTATTGCT CTTCCTTCTT TACGTTTACT TTATTTATTA GATGAAATTA

Ld TTCCTCAAGT TTCTTTAAAA ATTATTGGTC ATCAATGATT CTGGTCTTAT GAGTTTTCTG ATAATAAAAA TTTTGATTCT TACATATATC GATTATTAGA

Lb TCCCCCTGAT AACAATTAAA ATCATAGGCA ATCAATGATT TTGAACGTAC GAATATAGTG ACAATGTAAA ATTTGATTCG GTAATTTTCC GTCTTCTTGA

Ls AACCATCTAT TACTTTAAAA ACAATTGGTC ATCAATGATA CTGAAGATAT GAATATTCAG ATAATATTGA ATTTGATTCA TTTATATTTC GACTTTTAGA

Bm AACCAATTAT CTCTGTGAAG ATTTTAGGAA ATCAGTGATA TTGAACTTAT GAGTTTAATA CTCATATTAA TTATAATTCT TACATACTAC GGAATTTAGA

Cb GTCCTGTTGT GACTTTGAAG GTTATTGGAC ATCAATGGTA CTGGTCTTAT GAATATGAGG ATTCTTCTTC TTTTGATTCG TATATATTTC GTCTTCTTGA

Cs GTCCAATTAT CACTCTTAAG GTTATTGGTC ATCAGTGATA TTGATCATAT GAGTATGAGG ATAATTATTT ATTTGATTCA TATATATTTC GGCTTTTAGA

Hm TTCCTGAAGT TACAGTCAAA GTCATTGGAA ATCAATGATA TTGATCATAT CAATATTCAG ATAATATTGA ATTTGATTCT TACATATTTA AATATTTGGA

Ib AACCTATGAT TAGTATTAAG TCTATTGGTC ACCAGTGGTA TTGGTCATAT GAGTATGGAG ATAGAATTGA GTTTGACTCA TATATGATGC GATTGTTAGA

Pc TTCCCGCTAT GAGATTAAAA GTGGTCGGAC ATCAGTGATT TTGGTCTTAT GAGTACGGGG ATAATATTGA ATTTGATTCA TATATGTTTC GACTTTTGGA

Ph TTCCCGCTAT GAGATTAAAA GTGGTCGGAC ATCAGTGATT TTGGTCTTAT GAGTACGGGG ATAATATTGA ATTTGATTCA TATATGTTTC GACTTTTGGA

Pp ACCCTTCAGT TACCCTTAAG GTTATCGGCC ACCAGTGGTA TTGGTCCTAT GAGTACGGAG ACAGAATTGA GTTTAATTCT TACATGTTTC GTCTTTTAGA

Dm ATCCATCTGT AACTTTAAAA AGAATCGGCC ATCAATGATA TTGAAGTTAC GAATATTCAG ATAATATTGA ATTTGATTCA TATATATTTC GATTATTAGA

Ld AGTGGATAAA TGTGTTTTTC TTCCTAAGCT AGTTCAAATT CGAGGATTAG TTACTTCAGA AGATGTTCTT CATTCATGGG CAGTTCCGAG ACTTGGTTTA

Lb TGTTAATAAA GCTCTAATTC TACCAATTAC CACCCATGTC CGTCTTCTCT TATCGTCAAA TGACGTGATC CACTCTTGAA CTCTGCCCTC ATACGGGTTA

Ls AGTAAATAAT CGAACAATTT TACCATTCAA AACACAAATT CGTATTTTAG TTACAGCAGC TGATGTTTTA CATTCATGAG CTATACCATC TTTAGGTGTA

Bm AGTCGATAAT AACTTGGTTC TACCAGTTGG GGTAGAAACT CGAGCAATTA TCACATCGAG AGATGTGATT CACTCATGAG CAATCCCACC ATTGGGTGTG

Cb AGTAGATAAG AGGGTAAAAG TTCCTTTGAA TAGGGAGAGT CGGGTTTTTG TGACATCTTC TGATGTGATT CATTCTTGGA CTGTTCCGTG TTTAGGGGTT

Cs GGTAGATAAG AGTGTAAAAG TTCCGGTAGG AGGAGAGAGT CGGGTTTTTG TTACTTCTTC AGATGTAATT CATTCTTGGA CTGTTCCTTG CATAGGTGTG

Hm TGTAGATAAT CGAACTGTAC TTCCTGTAGA CACTAATATT CGAATAATTA TTACATCTTC TGATGTAATT CACTCATGAA CAATCCCTAG ATTAGGGGTT

Ib AGTAGATAAT CGGACAGTAA TTCCTGTAGG AATAGAAATT CGGATACTAA TTACATCTAC TGATGTAATT CACTCATGAA CTATTCCTAC ACTGGGGGTA

Pc GGCTGATTTA AGCGTGTTTA TCCCTTATTT GACTGAAGTG CGTGCTATTG TAACATCTGC CGATGTTATT CATTCTTGAG CAATTCCTAT AATAGGAGTA

Ph GGCTGATTTA AGCGTGTTTA TCCCTTATTT GACTGAAGTG CGTGCTATTG TAACATCTGC CGATGTTATT CATTCTTGAG CAATTCCTAT AATAGGAGTA

Pp GTCTGATTGC AGGGTGTATA TTCCTTCTAG AACAGAAATT CGTGCTATCA TTACTTCTTC GGATGTCATT CATTCTTGGG CTATTCCTAG ACTAAGGGTT

Dm TGTTGATAAC CGAGTAGTTT TACCTATAAA CTCACAAATT CGAATTTTAG TAACAGCTGC TGATGTTATT CATTCTTGAA CAGTACCTGC TTTAGGAGTA

Ld AAAATAGATG CTTGCCCAGG TCGTATTAAT TTTAGTCTTC GTTCTGGGAA AGTTTATGGA CAATGCTCAG AAATTTGCGG TGTCAATCAT TCTTTTATAC

Lb AAAATTGACG CCAATCCAGG CCGCCTAAAT TACAGCTATC GTTCGGGCTA CTTTTATGGT CAGTGTTCAG AAATCTGTGG GGTTGACCAC TCATTTATAC

Ls AAAATTGATG CTAATCCTGG TCGATTAAAT AATATTAATC GTCCGGGATT ATTTTATGGG CAATGTTCTG AAATTTGTGG AGCTGTTCAC TCATTTATAC

Bm AAAATAGATG CAGTACCTGG CCGAATTAAT TCAATTTCAA TATCTGGTCT ATTTTATGGG CAATGTTCTG AAATTTGTGG ATCTCTTCAT TCATTCATGC

Cb AAGGTTGATG CTATTCCGGG ACGGTTGAAT TATCCTTCTC GAGTGGGTTT AGCTTATGGT CAATGTTCGG AAATTTGTGG TTCAATGCAT TCCTTTATAC

Cs AAGGTTGATG CGATTCCTGG TCGGTTAAAT TATCCTTCCC GGGTTGGGTT AGTATACGGG CAATGCTCAG AAATTTGTGG ATCAATACAT TCTTTTATAC

Hm AAATTAGATG CCAATCCTGG ACGATTAAAT TTAGGTAATC GATTAGGATT ATTTTTTGGT CAATGTTCAG AAATTTGTGG AATTTTACAT TCATTTATAC

Ib AAAATAGATG GAGTTCCTGG TCGTTTGAAC TCAAGTAATA TTTGTGGTTT AATGTATGGT CAGTGTTCAG AAATTTGTGG AAGTTTTCAT TCATTTATAC

Pc AAAGTAGACG CTATTCCTGG GCGTTTAAAC TACTCATTTA AAATTGGCAC ATCTTATGGT CAGTGTTCTG AGATTTGTGG TGCTTATCAC AGGTTTATGC

Ph AAAGTAGACG CTATTCCTGG GCGTTTAAAC TACTCATTTA AAATTGGCAC ATCTTATGGT CAGTGTTCTG AGATTTGTGG TGCTTATCAC AGGTTTATGC

Pp AAGATAGATG CTGTCCCTGG ACGGCTAAAC TACTCTCATA AACTTGGGAG TCTGTATGGT CAATGCTCTG AGATGTGTGG GGCTTACCAC AGGTTCATAC

Dm AAAGTTGACG GTACACCTGG ACGATTAAAT TTTATTAATC GACCGGGTTT ATTTTATGGT CAATGTTCAG AAATCTGTGG GGCTAATCAT AGATTTATAC

Ld CTGTAGTTGT TAATTTTGTT GAATTAAATA AATTTCATCT TTGACATGAT TGCCATATAG TCACACAAAG TCCTTGACCT TTTTTTTTAA GTGTAGCCAT

Lb CTATTAAAGT TGGTTTCACT TCTCTGGACT GGTTTAAGAA TTTTAGAGAC TTTCATCTAG TAGATATCAG GCCGTGGCCT TTAATAATAA GCTTGACTAC

Ls CTATTGTTAT TGAAAGAGTT CATAAAAATA GATTTATTAA TTGACACCCT TATCATTTAG TAGATGTAAG TCCATGACCA TTAACAGGTG CAATTGGAAC

Bm CAATTTGCGT AGAAGCTATT CCTCTAAAAA ATTGGTTAAA ATGACACCCA TTTCATATAG TCCAATTGAG ACCTTGACCA TTGATTTGTT CTTTTAGACT

Cb CGATTTGTTT AGAGGTGGTT CCTCAAGAGG AGTTTTTTCG ATGACATCCG TTTCATGTGG TAGATTTAAG GCCCTGACCA TTGGTAATGT CATTGTCTGT

Cs CGATTTGTTT GGAGGTTATT TCTCAAAGTG ATTTTTTTAG ATTTCATCCA TTTCATATGG TAGATTTAAG ACCTTGACCT TTAATGAAGT CGTTGTCTGT

Hm CAATTTGTGT AGAAATAGTA AAACCAGAGT GATTCTTAAA ATGATTTTTA TTTCATATTG TAGATGAGAG GCCTTGACCT TTATTTCTTT CATTTAGAGT

Ib CTATTTGTTT AGAAGTTTTA TCTGAGTCTC GATTTATATC TTGGCATCCT TTTCATATTG TTTCTATTAG TCCATGACCT ATTTTATGTT CTTTTTCTAT

Pc CGATTAAAGT CACTACTCTT CCAAAAGAAG ACTTTATAAA ATGACACCCA TTTCATCTTG TTGATGTAAG ACCTTGACCT ATTTTTTTAA GATTTTCTCT

Ph CGATTAAAGT CACTACTCTT CCAAAAGAAG ACTTTATAAA ATGACACCCA TTTCATCTTG TTGATGTAAG ACCTTGACCT ATTTTTTTAA GATTTTCTCT

Pp CAATTTCTGT CAAGACTGTT CCTAAGGCGG TATTTATCGC ATGACACCCA TTTCACCTGG TTGATTTTAG CCCTTGGCCT TTATATTTGA GTCTATCCTC

Dm CGATTGTAAT TGAAAGTGTT CCTGTAAATT ACTTTATTAA ATGACACCCT TTCCATTTAG TGGATTATAG TCCATGACCA TTAACAGGAG CTATCGGAGC

Ld TACAAACTTG ATTTTAAACT TATTTATAAG AATATTTATT ATTTTTTTAT GATCACGAGA TATCGTCCGA GAAAGAACAG GGCAAGGTTG CCACACTCTA

Lb AGCTAACACC ATTCTGTCTC TATACATCAA AATGTTTGTA TTTTTCCTAT GATCACGAGA TATCATACGA GAGAGAACCT TTCAGGGAAT ACACCCTTTA

Ls TATAATTTTA ACATCAGGTG TAGTTAAATG ATTCTTAACT ATATTTCAAT GATGACGAGA TGTAGTACGA GAAAGTACCT TTCAAGGTAA GCATTCAATT

Bm ATTTAGAATA ATCATTTTAA TATATGATTT TTTTTTAGTT TTGTTTGAAT GGTGACGAGA TGTGTGTCGA GAAAGAACTT TCCAAGGTTG ACACTCTTCC

Cb TTTCTCGTTA GAATTAAATT TGTATCATTT TTTGTTAGTA AGAGCTTTGT GGTGACGGGA CGTAATTCGA GAAAGAACTT TTCAAGGACA TCACTCTGAG

Cs ATTTTCTTTA GTAGTTAATT TGTATCATTA TATAGTTATC AGTGTATTAT GGTGACGAGA TGTAATTCGT GAAGGTACCT TTCAAGGTTG TCATACTAAG

Hm TTTTTTAAAT ATATTAAGAG CTTTAGTTTA TTTATTAATT TTTTACATGT GAATACGAGA TATAATTTCT GAAAGAACTA TGCAAGGAAT ACATACTTTA

Ib TATATCTTTT GTGATTAATT CTTTGTATTA TATATTAGTT ATTTTTTGTT GATGGCGTGA TGTCATTCGA GAAAGAACGT TTCAGGGTTT TCACATGAAA

Pc TTTATTTTCA GCGTCCATAA CATTGTGTTG AATTTTAATT GTTTCTTTTT GGTGACGAGA CGTTACTCGA GAGGCTACTT TTCAGGGTAA ACATACAATA

Ph TTTATTTTCA GCGTCCATAA CATTGTGTTG AATTTTAATT GTTTCTTTTT GGTGACGAGA CGTTACTCGA GAGGCTACTT TTCAGGGTAA ACATACAATA

Pp TTTATGTTTT GCAGTGGCTG TTCTGTGTAT TTTACTTGTG CTTTCTTTGT GGTGACGTGA TGTAATTCGT GAATCAACTT TTCAAGGTAA ACATACTATC

Dm TATAACAACT GTATCAGGTA TAGTAAAATG ATTTTTAACT GTATATCAAT GATGACGAGA TGTATCACGA GAAGGAACAT ACCAAGGATT ACATACTTAT

Ld AAGTTAATTA AATTTGGTAT AGCTTTATTC ATTTTATCAG AAGTAATATT TTTTTTTTCT TTTTTTTGAG CATTTTTTGA TTATGCATTA AATCCTTCTT

Lb AAATCACTTA AATACGGAAT AATCCTGTTC ATTACTTCTG AAGTTATATT TTTCTTGTCC TTTTTCTGAA CATTTTTACA TTCGGCCCTT AGCCCCACAA

Ls TCAGGAATAC GATGAGGAAT AATTTTATTT ATTACATCCG AAGTATTTTT TTTTATCTCA TTTTTTTGAG CATTTTTTCA TAGAAGATTA TCACCATCTA

Bm AATGGATTAA AGATTGGATT TATTATATTT ATTTGTTCAG AAGTCTTATT TTTTTTTTCT TTCTTTTTTG GATATTTCTT TCTTTCTCTT AATCCGGATG

Cb GAAGGATTGG TTCTTGGAGT GCTTTTATTT ATCTGTTCAG AAGTAATGTT TTTTTTTTCT TTTTTTTTTG GGTTTCTTTT TTCTGCTTTA TGTCCAGATA

Cs GAGGGTTTGG TGAGTGGGGT TTTGTTATTT ATTTGTTCGG AGATTATGTT TTTTTTTTCT TTTTTTTTTG GGTTTTTGTT TTCATCTTTA TGTCCAGATG

Hm AAAGGAATTA AAATAGGAAT AGTATTATTT ATTACATCTG AGGTTATATT TTTTTTTTCT TTCTTCTGAA GATTGGGATA TTATATAGTA AGTCATGAAT

Ib AAAGGTTTAT ATATAGGAGT TTCAATATTT ATTATTTCAG AAGTAATATT TTTCTTTTCT TTTTTTTTTG GTTATTTTTT TTCTAGTTTA GTCCCAGACG

Pc GAAGGATTGC GTTTAGGAAT GCTTATGTTT ATTGCTTCAG AGGTAATGTT TTTCTTTTCG TTTTTTTATG CTCTGTTTTT TCTTTCTTTA AGGCCTGACG

Ph GAAGGATTGC GTTTAGGAAT GCTTATGTTT ATTGCTTCAG AGGTAATGTT TTTCTTTTCG TTTTTTTATG CTCTGTTTTT TCTTTCTTTA AGGCCTGACG

Pp GAGGGTATCC GTTTAGGAAT AATTTTGTTT ATTATGTCTG AGGTAATGTT CTTCTTCTCG TTTTTCTATG GTCTTTTCTT TCTAGCTCTT AACCCAGATG

Dm GCAGGTTTAC GATGAGGAAT AATTTTATTT ATTTTATCAG AAGTTTTATT TTTTGTGAGA TTTTTTTGAG CTTTTTTTCA CAGAAGTTTA TCACCCGCTA

Ld TAGAAATTTG GCCGCCCAAA GGTGTGGTCC CTCTTAATCC TATGCACGTT CCTTTATTAA ATTCTTTAAT TTTGGTTTCT TCAGGATTGA CAATTACTAG

Lb ATGAAATTTG ACCAAGATGG GGTGTTGAGC CTATTAATCC CTTTGGAATC CCTTTGCTGA ACACTCTAGT CTTAGTTTCA TCAGGGGTTT CTATTACATA

Ls TCGAAATTTG ACCTCCTAAG GGAATCCAAC CTTTTAATCC ATTTCAAATT CCTTTTCTTA ATACTGTAAT TTTAATTTCT TCAGGAATTA CCATTACATG

Bm TGGTCTTTTG ACCCCCAAAG GGCTTAATAG TAGTTGATTT TTTATCCGCT CCCACTCTTA ACTCAATTCT TCTTCTATCT AGGGGAGTAA GAATTACTTG

Cb TTGAGATTTG ACCTCCTTTG GGGATTGAAC CACTAAATTT TATGATGGTT CCATTAATGA ACACTTTAAT TCTTTTATCT AGGGGAGTGT CAATTACTTG

Cs TTGAAATTTG GCCTCCTGTG GGGAATGAAC CATTAAGATT TATAATGGTT CCTTTTTTAA ATACACTTAT TCTTTTATCT AGAGGAGTTT CAATTACATG

Hm ATATTCTTTG ACCTCTTTTA GGTATTATAA GATTAAATCC ATCTACAGTG CCATTATTGG GTACAATAAT TTTATTAAGA TCTGGAGTAT CTGTAACATG

Ib TAGAAATTTG ACCACCAGTG GGAGTTCAAT CTTTAAGGTT CATAGATGTT CCATTATTAA ATACAATAAT TCTTCTATCT AGAGGAATTT CTATTACTTG

Pc TGTCATTGTA CCCTCCTGTG GGTGTTAGCC CTGTAGGCGT TTTAGGAGTT CCTCTTTTAA ATTCTATTTT ATTACTCTCT AGCGGTGTGT CTATTACTTG

Ph TGTCATTGTA CCCTCCTGTG GGTGTTAGCC CTGTAGGCGT TTTAGGAGTT CCTCTTTTAA ATTCTATTTT ATTACTCTCT AGCGGTGTGT CTATTACTTG

Pp TAACTTTATT TCCTCCGGTG GGTCTAGCGT CAATAGGGCT ACTTGGTGTG CCTCTGTTAA ACTCGTTTTT ACTCTTATCA AGTGGGGTCA GGGTAACTTG

Dm TTGAATTATG ACCTCCTATA GGAATTATCT CATTTAATCC ATTTCAAATT CCTTTATTAA ATACAGCTAT TTTATTAGCT TCAGGAGTTA CTGTAACTTG

Ld AGCGCACATA TTTTTAAATA AGAGGAAAGC TATGATATGG ACCTCTCTAA CTGTTATTTT AGGAATTTAT TTTACTATCT TACAAATTTT AGAATACTTT

Lb TTCTCACCAT AGAATAAATT TTAATTTTAC CATTCTCTGA GTAGTAATTA CAGTTCTTTT GGGAGGGTAT TTTACGATTC TACAGCTCAT GGAATATTTT

Ls AGCTCACCAC TCACTAAATA ATAGTCAGAC CATTCAAAGA TTAGTAATTA CCATTATTTT AGGATTATAT TTTACTATTC TTCAAGGAAT TGAATATTTT

Bm AGCACATCAT AGAATTAATT TATCTGAGGC AAAAATGGGA TTAGTTTACA CAGTTTTTTT GGGAATTATA TTCTCTATAA TTCAATTAAT TGAATATTTC

Cb ATCCCATCAT TCTATTGATT GAAAGAATTC TCTTTTTGGG ATGGTTATTA CAGTATTTCT TGGATTTGTG TTTTCTTTTC TTCAATATGA AGAATATTTT

Cs GTCTCATCAT GCCTTAAATT TATTTAGATC ATTATATGGA ATAATGGTTA CAGTGTTCTT TTGATTGGTC TTTTCTTTTT TTCAATTTGA TGAGTATTTT

Hm ATGCCATAAT GAGCTTAATC TCAGAAGAAT AAAAAATTCA TTATTAATTA CAGTAATTCT TGGAATAGTA TTTGCAGCTC TTCAAATATG AGAATATTTT

Ib GTCCCACCAT TCTTTAAATT TTACTAACTG CTTATTAGGG ATAATTTTCA CTGTTATCTT AGGTTTGATT TTTACTTTTT TTCAATTTAT AGAGTATTTT

Pc AGCTCATTAT GAGCTTAATA TTTCTTCTAG GCTTATCGGC TTATTAATCA CTTTAATTTT AGGTCTAGTG TTTCTAATAT TTCAGGCTGT TGAATATTTT

Ph AGCTCATTAT GAGCTTAATA TTTCTTCTAG GCTTATCGGC TTATTAATCA CTTTAATTTT AGGTCTAGTG TTTCTAACAT TTCAGGCTGT TGAATATTTT

Pp ATCTCACTAC GAGATTGATG TACCAAAGTC TTTACTGGCG TTGGCTATGA CTCTAGCTCT AGGTACTGTG TTTTTAGCCT TTCAGTATCT GGAGTACTTT

Dm AGCCCACCAT AGACTTAATC ATTCACAAAC TACTCAAGGA TTATTTTTTA CAGTTTTACT AGGAATCTAT TTTACAATTC TTCAAGCTTA TGAATATTTT

Ld TCATTTACTG ACTCAGCTTA TGGTTCTATT TTTTTTTTAA CTACGGGTTT TCACGGTTTT CACGTTATTA TTGGCACTAT CCTTATTTTA GTTAGTCTTG

Lb TCTATCATGG ACTCAGTTTA TGGCTCAATT TTTTTTATCT CTACAGGCTT TCATGGCATC CATGTTCTAG TTGGCACTCT GATGATTCTA TATTCCCTCA

Ls TCAATTGCTG ATGCTATCTA TGGATCTTCA TTCTTTATAG CAACAGGTTT TCATGGAATT CATGTAATAA TTGGAACTAC ATTTATTTTA ATAATACTTA

Bm ACTATTGCTG ATAGACCTTT TGGGTCTATG TTTTTCTTAG CAACTGGATT TCATGGTATC CATGTTTTAG TAGGAACAAT TTTTATTATT ATTTCTTTTG

Cb ACGATAGCAG ATAGAGTATA TGGATCTTTT TTTTTTCTAA TAACGGGATT TCATGGGATT CATGTGATTG TAGGAGTTTT GTTTATTATA GTAAGGTTAT

Cs ACTATAGCTG ATAGAATTTA TGGATCTTTT TTTTTTTTGA TGACAGGGTT TCATGGAATT CATGTAATGG TTGGAGTGTT GTTTATTTCT GTTAGATTGT

Hm ACTATAAGAG ATGGTGTATA TGGGTCGTTG TTCTATATAA TAACTGGATT TCATGGATTT CATGTTATTG TTGGAACAAT TTTTTTATTT ATTATTTTTT

Ib TCTATAGCTG ATAGGGTTTA TGGGTCACTT TTTTATATTT CTACAGGCTT TCATGGAATT CATGTAATTG TAGGCACATT ATTTATTATT GTTTCTTTTA

Pc ACTATGGCTG ATAGAAGTTT TGGCTCAGTG TTTTTTCTAA TAACCGGCTT CCACGGAGCA CATGTTTGTG TGGGAGTTGT GTTTATTACA ATTAGAACCA

Ph ACTATGGCTG ATAGAAGTTT TGGCTCAGTG TTTTTTCTAA TAACCGGCTT CCACGGAGCA CATGTTTGTG TGGGAGTTGT GTTTATTACA ATTAGAACCA

Pp AGGATCTCTG ATAGTGCTTA TGGGTCTATC TTTTACCTTA TAACTGGCTT TCATGGGTTT CACGTTATGG TGGGAGTGGT ATTCATTTTA GTAATAACAG

Dm ACTATTGCAG ACTCAATTTA TGGATCAACA TTTTTTATAG CAACAGGATT CCACGGAATT CATGTATTAA TCGGAACAAC TTTTTTATTA GTATGTTTAT

Ld TTCGCATGTA CATGAACCAT TTTTCTAGAA AACGGCATTT AAATTTCGAA ATGGCTTGTT GATATTGACA TTTTGTTGAC TTTGTTTGAT TATTTCTTTA

Lb TTCGTCTATT CAGCTTTCAA TTCAGCTCTG CTCATCATTT AATGTTTGAA TTCTCTTGTT GATACTGACA CTTTGTTGAT TTAATCTGAC TTTTTCTTTT

Ls TTCGACAAAT AAATAATCAT TTTTCTAATT ATCATCATTT TGGATTTGAA GCAGCAGCAT GATATTGACA TTTTGTTGAT ATTGTATGAT TATTTCTTTA

Bm TTCGCCTTTT AAATAATCAA TTTTCAAAGA ACCATCACGT CGGCTTTGAA ATGAGTTGTT GATATTGACA TTTTGTAGAC GTAGTATGAT TGTTTTTATT

Cb TTCGAACTTT AGTTGGGCAT TTTTCAAAAA GTCATCACTT TGGATTTGAA GCTGCTGCTT GGTATTGGCA TTTTGTTGAT GTAGTTTGGT TGTTCTTGTT

Cs TTCGACTAAT AATAGGTCAT TATTCAAGTT TTCATCACTT TGGTTTTGAG GCTTCTGCTT GATATTGACA TTTTGTGGAT GTAGTATGGT TGTTTTTGTA

Hm TACGATTAAA AAATTATCAT TTTTCTAGAC ACCATCATTT AGGATTTCAA GCAGCAGCTT GATATTGACA TTTTGTTGAT GTTGTTTGAA TTTTTTTATA

Ib TTCGAATAAT GAAATATCAT TTTTCCATTC ACCATCATTT GGGGTTTGAA TTTTCTATTT GATATTGACA TTTTGTAGAC GTAGTGTGAT TGTTCTTATT

Pc TTCGGCTTTA CTTAAATCAC TATAATAATA ATCATCACTT AGGGCTTGAG CTAGCTGCAT GATACTGACA CTTTGTCGAC GTAGTGTGGT TATTTTTATA

Ph TTCGGCTTTA CTTAAATCAC TATAATAATA ATCATCACTT AGGGCTTGAG CTAGCTGCAT GATACTGACA CTTTGTCGAC GTAGTGTGGT TATTTTTATA

Pp TTCGTTTAGC TAAGGGTCAT TTGAACTCAA GACATCATAC AGGGTTTGAG CTATCAGCCT GATACTGACA TTTCGTGGAT GTAGTATGGT TACTCTTATT

Dm TACGACATTT AAATAATCAC TTCTCAAAAA ATCATCATTT TGGTTTTGAA GCAGCTGCAT GATATTGACA TTTTGTCGAT GTAGTTTGAT TATTTTTATA

Ld TATTTCTATT TACTGATTTT CTATTTTTGA CTTATTATTT ATTATTAACT CAGAAGCTTT TATATTTATT TTTTTTTTGA ATCTTTATGG TCTTCTTTCT

Lb TCTATCAATT TATTGATTTT CAATTTTTGA CCTAATCTTA TTAATTATTA AAGAAATGTT TATTTTGATT TTGGCTTTCA ACATCTCTGG GATCTTTCCT

Ls TATTTCAATT TACTGATTTT CATCTTTTGA TTTATTATTT ATTATACATA ATGAATTTTT TAGATTTGTT TTAATTAATA ATTTTTTAGG CTTATTTCCA

Bm TGTTCGAGTA TATTGACTTT CAGTGTTTGA TTCTTTAATT GGGTTTTCTA TAGGATGATT TATCACAATT TTTTGTATTA ATGAAATGGG AATAGTTCCT

Cb TGTAACTGTA TATTGATCCT TCTTATATAG ATGTGCTGGT TTTTTTTTTG ATCTTTTAGT TTTTTTTTTT TTCTTTTCTA ATTTGCTAGG ATTAGTTCCT

Cs TGTGATGGTT TATTGGTCCT TCTTATATAA ATTTTTTTTT TTTTTTTTTT GGGKKGGRGT TTTTTTTTTT TTTTTTTCTA ATCTTCTAGG GTTAATTCCT

Hm TATTATGTTA TATTGACTAT CAATTTTTGA TATAAGATTA TGTTTAATGA AATCTATATT TTTTTTAATT TTAGTGTTTA ATATTTTAGG AATTTTTTGT

Ib TTTAAGAGTA TATTACTTTT CCATTTTTGA TTCATTAAGT TGAATAATTT CATCATCATT TACAATAATT TTAATATGTA ATCAGTTAAG TATAGTCCCA

Pc CCTAACTCTT TACTGAATAT CTTCTTTTGA CATTCTTTTT CCTTTCTTAT TAATAAAATT TATAATAATT TTAACCCTTA ATACAATTTC ACTTATGCCA

Ph CCTAACTCTT TACTGAATAT CTTCTTTTGA CATTCTTTTT CCTTTCTTAT TAATAAAATT TATAATAATT TTAACCCTTA ATACAATTTC ACTTATGCCA

Pp TGTGTCACTT TACTGAATGT CTATATTTGA TGCAACCTTA GTCTGTCTTC ATGAATTGTT TTATTGTGTA TTGAGCATAA ATGTTATCTC GTTAATTCCT

Dm TATCACAATT TACTGATTTT CTGTATTCGA CTTTTTAGGA CTTTTACATA AAGAATTTTT TTCATTAATT TTATTTAATA ATTTCATAGG ATTATTTCCA

Ld TATGTTATAG CAGTTTTTAG TCACATATCA ATTTCTTTTT CTTTGAGAAT ATTATTTTGA ACAAGGATTA TGTTGATAAG GCACTTAGTA CCTTTAGGGT

Lb TTTACATTCA CCCTAACTAG GCATATAAGA ATAACTTTTT CTTTAGCCTT TCCTATATGG TTAAGACTGA TAATTTTCGC CCACCTTGTC CCTTTAGGGT

Ls TATATTTTTA CTAGAACAAG TCAATTAGTT TTAACTTTAA CTCTTTCACT ACCTTTGTGA TTAAGATTTA TAATTTTTGC TCATTTAGTT CCAAATGGAA

Bm TATTTATTCA CTCCGACAAG ACATTTTAGT TTAAATCTTT GTCTTGCCCT TCCTTTATGA CTAACTGGAA TTATTTTTTC TCATTTGGTT CCTGAAGGGA

Cb TATGTATTTA CTCTTTCTTC CCATTTGTGT GTAAATTTGA GTGTTTCTTT AGTTTTGTGG CTAGGAGGGG TGGTTCTTTC TCATATAGTT CCTTTAGGTT

Cs TATATGTTTA CTTTGTCTTC TCATTTGTGT GTAAATATGA GGGTTTCTTT GGTGATGTGA ATGGGAGGAG TAATTCTTTC TCATATAGTT CCTTTAGGAT

Hm TTTACATTTT CTGTAACTAG ACATTTAGTT ATTAATTTAT CTTTAGGATT TTCTATTTGA GTAGGAACTT TATTATTAGC CCATTTGACA CCAATAGGGT

Ib TTTGTTTTTG GTCCTACTAG ACATTTATCA TTTAATTCGG CTGTTGCTTT ATCAAGATGG TTAGCAGGGA TTATTGTTTC ACATTTTGTT CCTTTAGGAA

Pc TTGGTGTTGC CTTGCACTTC GCATTTAAGA GTTAATTTAG GACTTTGTTT ACCTTTATGG ATAAGAGGGG TTGTTCTAGC TCATCTTCTT CCTTACGGGA

Ph TTGGTGTTGC CTTGCACTTC GCATTTAAGA GTTAATTTAG GACTTTGTTT ACCTTTATGG ATAAGAGGGG TTGTTCTAGC TCATCTTCTT CCTTACGGGA

Pp TTTACTTTAC CTTTTACCTC TCATATTTCT GTTAATTTGG GGATATGTTT GACTCTGTGG TTAAGTGGCT TACTTTTGGC TCATTATTTA CCTTTGGGGA

Dm TATATTTTTA CAAGAACAAG ACATTTAACT TTAACTTTAT CTTTAGCTTT ACCTTTATGA TTATGTTTTA TATTATTTGC TCATTTAGTT CCTCAAGGAA

Ld GTCCTAATGG TTTAATATTT TTTATGGTTA TTATTAGAAT TTTAATTCGC CCAATCACTT TAGGAGTACG TTTATCTGCT AATATTATAG CTGGACATGT

Lb GTCCCACAGT TTTAATACCG TTTATGGTAT TAATCAGTCT AATTATTCGT CCGCTAACTT TAGCTGTTCG TCTCGCAGCC AATATAATTG CTGGGCATAT

Ls CACCAGGTAT TCTTATACCA TTTATAGTAT GCATTAGAAA TATTATTCGT CCGGGAACAT TAGCAGTACG ATTGACAGCA AACATAATTG CTGGACATTT

Bm GACCAATAGG GTTGGCTCCA CTATTAGTGA TTATCAGTTT ATTAATTCGT CCTATTTCCT TGAGAATTCG GTTAATATCA AATATTATGG CAGGACATAT

Cb CTCCTGTATT TCTTCTTCCT TTATTGGTTT TAATTAGGAC ATTGATTCGT CCGTTAACTT TGGCTATTCG GTTGATGGCT AATGTGATAG CGGGCCATTT

Cs CACCAGTGTT TCTTCTTCCT TTGTTGGTAG TAATTAGTAT AGTGATTCGC CCTCTTACTC TTTCTATTCG GTTAATGGCT AATGTAATGG CTGGACATTT

Hm GTCCTATGGT TTTAGTTCCT TTTATAGTAG TAATTAGAAT AATAATTCGA CCTATTACTT TATCTTTACG ACTAATAGCT AATATATTAG CTGGACATAT

Ib GACCAATATT TTTAACTCCT TTTTTATTTA TTGTAAGTTG TTTAATTCGA CCTGTAGCGT TAAGAGTTCG GTTAATATCA AATATAATGG CTGGACATAT

Pc GTCCAATTAT ACTAAGTCCG TTCTTAGTGG TAATCAGAGT CTCGATTCGT CCTGTATCTC TAAGAGTTCG ACTTCTAGCG AATATCACAG GAGGACATTT

Ph GTCCAACCAT ACTAAGTCCG TTCTTAGTGG TAATCAGAGT CTCGATTCGT CCTGTATCTC TAAGAGTTCG ACTTCTAGCG AATATTACAG GAGGACATTT

Pp GTCCAATGGT TCTGGGTCCG TTCCTAGTTC TAATTAGGGT TCTTATTCGG CCAATTAGGC TAAGAGTGCG GCTGATGGCT AACATTTTAG GTGGCCATAT

Dm CACCCGCTAT TCTTATACCT TTTATAGTAT GTATTAGAAA TATTATTCGA CCTGGAACAT TAGCTGTTCG ATTAACTGCT AATATAATTG CTGGACATTT

Ld AATTTTAGGA TTAACTGTAC AGTTTTTAAT CTTAGAGTTT GCTGTAGCTT TAGTTCAACC TTATGTTTTT TTTACTTTAT TATGTTTATA TATAAAATGA

Lb GATTTTATCG TTATTAGCTG AGTCTTTGCT TTTAGAATTG GCAGTCGCAA TAATTCAGCC CTATGTCTTC TTCATCCTAT TAACCCTTTA TAGCCAGTGA

Ls AATCTTAACT TTAATTATTC AAATTTTAAT ATTAGAAATA GCTGTAGCAT TTATTCAATC TTATGTAATT GCAATTTTAA TTACTCTTTA TTCGAGATGA

Bm GATTTTAAGA TTAATGGTGG TATTTATAAG TTTTGAACTA TGTGTCGGAA TTGTTCAAGC TTACATTTTT TCTTGCTTAT TAGTAATGTA TTGATCATGG

Cb AATTATAAGG TTGTTTATTG AGTTGTTGTT TTTTGAAATG TGTGTGGCTG TTGTTCAGGC TTATGTTTTT AGCAGGTTAA TAGTGATATA TTTAGCTTGG

Cs AATTATTAGG TTGTTTTTTG AGGTGTTGTT TTTTGAAATA TGTGTTTCTA TTGTCCAGGC TTATGTATTT AGAAGTTTAA TAGTAATATA TTTGGCTTGA

Hm AATTTTATCA TTATTGTTGT TACTATATTT ATTTGAAATT GGAGTAGCAA TTATTCAAGC TTACGTTTTT TCAATTCTTC TTTCTTTATA TTGAGAATGA

Ib TATTATTGTA TTAATTGAAT CATTCTTTTT ATTTGAGTTA TGTATTTCTA TTGTTCAAGC TTATGTTTTT TCAAGTCTCT TAGCTTTATA CTATAAATGA

Pc AATTATAAAT CTTGCAGCGT ATGTTTTAGC TGCTGAGCTA TTTGTCTCAT TTATTCAGTC TTACGTTTTA AGTAAACTGG TTTCAATTTA CTGAGAATTG

Ph AATTATAAAT CTTGCAGCGT ATGTTTTAGC TGCTGAGCTA TTTGTCTCAT TTATTCAGTC TTACGTTTTA AGTAAACTGG TTTCAATTTA CTGAGAATTG

Pp TATTATGAGT CTACTAACAT ATTCTGTTTG TGTAGAGATG TTTGTAGCTA CTGTACAGGC TTATGTACTC AGAAAGCTCT TAAGAATCTA CTGAGAGTGA

Dm ATTATTAACT CTTATAGCTC AAATTTTAGT ATTAGAATCA GCTGTAGCTA TAATTCAATC TTATGTGTTT GCTGTATTAA GAACTTTATA TTCTAGATGA

Ld AATTTTGGTT CTCTTTTGGG AATTAGGTTA ACAGTTCAAA TTTTCACTGG TTTTTTTCTT TCTTTTCATT ATTCAGCTGA ATTTGACAAA ATTCTTAACA

Lb AACTTGGGGT CGCTTCTCGG GCTATGTCTT TCTATTCAAA TCTTAACAGG AGTGTTTTTA ACAATATTCT TTAAAGCAGA TTTTACCAGA GTAGTAAGAA

Ls AATTTTGGAT CTTTATTAGG ATTATGTTTG ATTATTCAAA TTTCTTCAGG TCTATTTTTA GCAATACATT ATTCAGCCCA TTTTTCAAGA ATAATTCACA

Bm AATTTTGGAT CTCTTTTAGG AATTAATCTT CTTCTTCAAA TTGTGAGAGG GATCTTTTTA GCAATACATT ATGAAGATAC TTTTGAAAGA GTGGTTAGAA

Cb AATTTTGGAT CTCTTCTTGG GGTTTGTTTA ATAATCCAAT TAGTTTCTGG GATTTTTCTT TCTTTTCATT ATTCTCCTAC TTTTTCTAGA GTAGTGATGA

Cs AATTTTGGAT CTTTATTGGG ATTGTGCTTA TTTATTCAAC TTGTGTCGGG GATTTTTCTT TCATTTCATT ATTCCCCTTC CTTTTTGAGA GTTGTTATAA

Hm AATTTTGGAA GTTTATTAGG GCTATGTCTC TTTATTCAAA TTGGATCAGG TTTATTTTTA TCACTTCATT ATAATTCAAA TTTTAGAAGT GTTATTTATA

Ib AATTTTGGTT CATTATTGGG GATATGTTTG ATAGTTCAAA TTTTTTCTGG TTTGTTTCTT TCTATACACT ATAATACTTC TTTTAATAGA GTTTTATCTA

Pc AAATTTTGGG TCTTATTAGG CTTGTTTCTT TCAATTCAGA TTTTAAGAGG TCTTTTTCTG GCTTCTCATT ATGAGGCTTC TTTTTGAAGT GTTATTTTAA

Ph AAATTTTGGG TCTTATTAGG CTTGTTTCTT TCAATTCAGA TTTTAAGAGG TCTTTTTCTG GCTTCTCATT ATGAGGCTTC TTTTTGAAGT GTTATTTTAA

Pp AATTTTGGTT CTCTTTTGGG TTGTTTTTTA GTTGTTCAAG TGTTTACTGG ATTTTTGTTG TCTACTCATT ATGAGGCTTC TTTTGAAAGG GTACTCTTGA

Dm AATTTTGGAT CATTACTTGG ATTATGTTTA ATTATTCAAA TTTTAACCGG ATTATTTTTA GCTATACATT ACACAGCTGA TTTCTATAGT GTTAATCATA

Ld TATGCAACGA AATCAGGTAT GGGTGAATGT TACGTTTTTT ACATTCTACC GGGGCGTCAA TGTTTTTTAT TTTATGTTAT TTACATATCG GGAAAGCTTT

Lb TTATAAATAA TATTAATAAC GGGTGAATCA TCCGGTTTAT TCATTCCACT GGGGCATCCA TATTTTTTAT TATCTGCTAC GCCCATGTCG GAAAAGCACT

Ls TTTGCCGGGA TGTAAATAAT GGTTGAATTT TACGAACAAT TCATGCAAAC GGAGCTTCTT TTTTTTTCAT TTGCTTATAT TTACATGTAG GACGAGGAAT

Bm TAATAAATGA TATAAATAGA GGTTGATTAA TTCGCTTTAT TCATGCGAAT GGGGCTTCTT TATTTTTTGT TCTTCTGTAC TTTCACATCG GTCGAGGGTT

Cb TTGTTGATGA TGTTCCGTTT GGATGAATAT TTCGAAGAAT TCATGCTAAT GGGGCTTCGT TTTTTTTTTT TTGGGTTTAT TTACACATTG GTCGGGGTTT

Cs TTGAAGATGA TGTTCCATTT GGATGGATAT TTCGTAGAGT TCATGCTAAT GGAGCTTCAT TTTTCTTTTT TTGTATTTAC CTTCATATTG GGCGGGGTTT

Hm TAATAAATGA TGTTAATCAT GGATGAATTT TACGTGTAAT TCATGCAAAT GGGGTAACAA TAATATTTAT TTTTATGTAT ATTCATATTG CTCGTGGACT

Ib CATGTAATGA TGTTAACTTA GGGTGATTGA TTCGTTATAT TCATGCTAAT GGAGCCTCAA TATTTTTTAT ACTTGTATAC TGTCATATTG GTCGAGGTTT

Pc TTGATTTTGA TGTAAATAGA GGGTGGTTGA TTCGTAGTTT TCATGCTAAC GGCGCTTCTT TTTTCTTCAT TCTTGTCTAC GTTCATATTT GGCGTGGTTT

Ph TTGATTTTGA TGTAAATAGA GGGTGGTTGA TTCGTAGTTT TCATGCTAAC GGCGCTTCTT TTTTCTTCAT TCTTGTCTAC GTTCATATTT GGCGTGGTTT

Pp TCGAGTTTGA TGCTTTAGAA GGGTGATTAG TTCGTAGCTT ACATGCTAAC GGAGCTTCTT GATTTTTTAT TCTGGCTTAT TTCCATATTT GACGCAGTCT

Dm TTTGTCGAGA CGTTAATTAT GGTTGATTAT TACGAACTTT ACATGCTAAC GGTGCATCAT TTTTTTTTAT TTGTATTTAC TTACATGTAG GACGAGGAAT

Ld AGTTTATAAG TCTTATTGAG CATCAGGTTT AATAATTTTA TTATTAATTA TAGGTACAGC TTTCCTTGGT TATGTTCTGC CATGAGGTCA AATATCATTG

Lb ATTTTTCTCT TCATTTTGAG TTTCTGGACT AGTGTTAATT CTTTTACTCA TAATAGAGGC TTTTCTAGGC TATGTCTTAC CATGAGGACA GATATCATTT

Ls TTATTATAGT TCTTATTGAT TTATTGGAAT TTTAATTTTA TTATTAACTA TAGCAACTGC CTTTGTAGGA TATGTTTTAC CTTGAGGACA AATATCTTTT

Bm GTACTACGGG AGATATTGGA TTGTGGGAGT GATAATTATA TTCATTCTCA TAGGAACAGC ATTTGTAGGG TATGTCCTCC CATGAGGACA AATGTCATTT

Cb ATATTTAAGG AAATATTGAA TGAAAGGGGT TTTAATCTTT TTTTTTTTAA TAGGGACGGC TTTTATGGGA TATGTTCCCC CATGAGGTCA AATTTTTTTG

Cs GTATTTTGGA AGATATTGAA TTTCTGGCGT TTTAATTTTG TTTTTATTAA TAGGAACAGC ATTTATTGGA TATGTTCTTC CGTGAGGTCA GATATCATTG

Hm TTACTATAAA TCTTATTGAT TAGTTGGGAT CTTAATCCTA CTATTAACAA TAGGAACTGC ATTTTTAGGA TATGTTCTTC CTTGGGGGCA AATATCATTT

Ib ATATTTTGGG AGTTTCTGAT TTTCAGGAGT GATTATTCTT TTATTATTAA TGGGTACCTC ATTTTTAGGT TACGTTTTAC CTTGGGGACA GATATCTTTT

Pc ATGATTTGGT TGTTTTTGAT TTTCAGGAAT TTCTATTCTT CTTCTTATAA TAGCAGCAGC TTTTATGGGG TATGTTCTTC CTTGAGGTCA AATATCTTTT

Ph ATGATTTGGT TGTTTTTGAT TTTCAGGAAT TTCTATTCTT CTTCTTATAA TAGCAGCAGC TTTTATGGGG TATGTTCTTC CTTGAGGTCA AATATCTTTT

Pp CTGGTTTGGT TGTTTCTGAG TGTCAGGTAT CCTAATTTTA TTGCTTATAA TGGCTATCTC TTTTTTGGGT TATGTCCTTC CTTGGGGGCA GATGTCGTTT

Dm TTATTACGGT TCATATTGAT TAATTGGAGT AATTATTTTA TTTTTAGTAA TAGGAACAGC TTTTATAGGA TACGTATTAC CTTGAGGACA AATATCATTT

Ld TGGGGGGCTA CTGTTATTAC TAATTTAATT TCAGTTATTC CTTATTTTGG TAAAGATATA GTGGAATGAT TATGGGGTGG TTTTAATGTG GGGACATTTA

Lb TGAGGAGCTA CAGTAATCAC AAATTTAATT TCTGTTATTC CTTACTTTGG CCCCCTTGCT GTTCAGTGAC TGTGGGGGGG CTTTAATGTT GGTGATCCCA

Ls TGAGGAGCTA CGGTTATTAC TAACTTATTA TCCGCCATTC CGTACTTAGG ACAAATATTA GTTCAATGAA TTTGAGGTGG ATTTGCAGTT GATAATGCCA

Bm TGAGGGGCCA CAGTTATTAC TAACCTTGTT TCGGCTGTCC CATTTATTGG GACAGATATG GTTATTTGAT TGTGAGGTGG ATTTTCCGTT GACAATCCCA

Cb GGGGGGGCAA CAGTTATTAC TAGTCTTTTA TCTGCTATTC CTTATATGGG GGGGTTTTTA GTTAAATGGG TTTGGGGGGG ATTTTTTGTT AAAGGACCGA

Cs TGAGGGGCAA CGGTAATTAC TAGTTTACTT TCTGCCATTC CATACTTAGG TAGGTTTTTA GTAGAATGGG TTTGGGGTGG ATTTTCAGTG AGAGGACCAA

Hm TGAGGTGCTA TAGTAATTAC TAATTTAATT AGAACTATTC CTTATTTAGG AGTAACATTA GTTGAATGGG TGTGGGGAGG ATTCTCTGTT AGAGAGCCAA

Ib TGAGGAGCAA CTGTTATTAC TAACTTAGTG AGAACTATTC CTTATGTAGG AGATCAGTTA GTTTATTGGT TATGAGGAGG GTTTTCTGTT AGTGAACCTA

Pc TGAGGAGCGA CTGTAATTAC TAATCTTTTA AGTGCTATTC CTATTGTTGG AAGAGATTTG GTTATTTGAG TGTGAGGAGG GTTTTCAGTT AGACATCCTA

Ph TGAGGAGCGA CTGTAATTAC TAATCTTTTA AGTGCTATTC CTATTGTTGG AAGAGATTTG GTTATTTGAG TGTGAGGAGG GTTTTCAGTT AGACATCCTA

Pp TGAGGGGCTA CTGTTATCAC AAATCTTTTC AGGGCTCTAC CTTTTGTAGG CTCAGAGTTG GTTACCTGGA TTTGAGGTGG ATTCTCAGTG GGGTCGCCCA

Dm TGAGTAGCTA CTGTTATTAC TAATTTATTA TACGCTATCC CTTACTTAGG TATAGATTTA GTTCAATGAT TATGAGGTGG ATTTGCTGTT GATAATGCCA

Ld CTTTAAATCG ATTTTATTCT CTTCATTTTA TTCTCCCTTT TTTGGTAGCT TTGATAGTTG TTATTCATAT TGTTTTTTTA CATTCTTCCG GTTCATCAAA

Lb CACTAACACG ATTCCTATCA TTTCATTTTA TCATTCCATT TATCATGATT GCTATAAGAG GAGTACATTT AATTCTTCTT CATGAGACTG GGTCATCAAA

Ls CATTAATTCG ATTTTTTACA TTCCATTTTA TTTTACCATT CATTATTTTA GCAATATCAA TTATTCACTT ATTATTTTTA CATCAAACAG GTTCTAATAA

Bm CCCTAGTTCG TTTCTTTTCA ATCCACTTTG TCTTACCGTT TGTTATTTTG GCTATAGTTA TTCTTCATCT TTTATTTTTA CATTCAACTG GAAGATCCAA

Cb CTCTTCATCG TTTTTTTTCT CTTCATTATC TTCTTCCTTT GATTTTATCA GTCTTTGTTT TTTTTCAAGT TTTTTTTTTT CAAAGGAAAA GGGGGGTTAA

Cs CCCTTCATCG GTTTTTTTCT CTCCACTATC TTCTTCCAAT TGTTTTGTTT TTTTTTGCAA TAGTTCATAT TTTTTTCCTT CATGAGAAAG GAAGGTCTAA

Hm CTTTAACTCG ATTTTTTTCA TTTCATTTTA TTTTACCTTT TGTAATTCTA GGGGCATCTG CTTTACATAT TATTTTTTTA CATAAGTATT TAAGATCGAA

Ib CACTAAATCG ATTTTTTTCT ATTCATTTTA TTTTGCCGTT TGTTTTAATG ATAGTGGTCT TAGTTCATAT TTTCTCCCTT CATAAAAGAG GAAGAAGGAA

Pc CTTTAGAGCG GCTGTTTACT CTTCACTTTC TTTTACCGTT TGTCTTATTG GGGTTTGTTA TAGCTCACAT TATTCTCCTC CACCAACACG GTTCTAGAAA

Ph CTTTAGAGCG GCTGTTTACT CTTCACTTTC TTTTACCGTT TGTCTTATTG GGGTTTGTTA TAGCTCACAT TATTCTCCTC CACCAACACG GTTCTAGAAA

Pp CGCTAGAGCG ATTTTTTAGT TCCCATTTTA TGCTAAGAAT GGTTTTGCTG TGCTTTGTTA TTTTCCACAT TACTTTTCTT CATGAGAATG GCTCTAGAAA

Dm CTTTAACTCG ATTTTTTACA TTCCATTTTA TTTTACCTTT TATTGTTCTT GCTATAACTA TAATTCATTT ATTATTCCTT CATCAAACAG GATCTAATAA

Ld TCCAATAGGT ATTGATAAAA TTTCTTTTTT TAATTATTTT GTGATTAAAG ACATAGTTAC TGTAGTCATC ATAATAGCAG TATTGTTTTT TTTTAGATTA

Lb CCCTCTAGGC ATAGATAAAG TTAGATTCAG AAAATTTTTT ATTATTAAAG ACCTAGTTAC CCTAGCTCTG GTTTTGTTAG GGCTAATTTT ACTTAGAACA

Ls TCCATTAGGA ATTGATAAAA TTCCATTTCA TCCTTTTTTT TCTATTAAAG ATTTATTTGG ATATATAATT ATACTTCTAA TTTTAATTTC ATTAAATTTT

Bm CCCACTAGGA AGGGATAAAG TTTACTTTCA TCCCTTATTT TCAATTAAAG ATATCCTCGG ATTAATTATT GTAACATTTT TCTTTCTTTC CACGGTCTTT

Cb TCCCATGGGG TCAAAAAAGG GATTTTTTGT TCCTTATTTT TTTTTTGTTG ATTTAGGAGG AATTTTTTTT TTTTTTTTTT TTTTTTTTAT GTTTGTTTTT

Cs CCCAATAGGA TCTGACAAGG TTTACTTTGT TCCTTATTTT CTTTCTGTAG ATTTGGTAGG AGTTTTTTTT TTTTTTTTTT TTTTTTTTTT ATTTATTTTT

Hm CCCCCTTGGT ACTGATATAA TTTCATTTCA CCCATTTTTT ACTGTTAAAG ATATCTTGGG TGTAGTATTA TTTTTATTTA GTTTATTATT TTTATCTTTA

Ib TCCTTTGGGA TGTTTAAAAA TTTCTTTTCA TCCTTATTTT TGAAATAAAG ACGTTTTGGG ATTTGTTGTT GTATTAATTA TTTTTACCGT TACATTAATT

Pc TCCTTTAGGA AGTGATAAAG TTTATTTTTA TCCTTACTTT TATCTAAAAG ATATTTTAGG AGGTTTTGTG TGTTTATTTT TATTTGTTTT GATTTGCATT

Ph TCCTTTAGGA AGTGATAAAG TTTATTTTTA TCCTTACTTT TATCTAAAAG ATATTTTAGG AGGTTTTGTG TGTTTATTTT TATTTGTTTT GATTTGCATT

Pp CCCTTTAGGG TCAGACAAGG TGTATTTTTA TCCTTACTTT ATGCTTAAGG ATTTGCTTGG AGGCTTAGTT GCGATGACTA TCTACTTCTC TCTAGGGCTT

Dm TCCTATCGGA ATTGATAAAA TTCCTTTTCA TCCTTATTTT ACATTTAAAG ATATTGTAGG ATTTATTGTA ATAATTTTTA TTTTAATTTC ATTAGTATTA

Ld TATAAGCCTT ATTTATTAAT AGATCATGAA AATTTTATTA CAGCTAACCC TATATTGACC CCTCCTCATA TCCAACCTGA ATGATATTTT TTATTTGCTT

Lb ATATCTCCTT TTATATTTAT AGACCCTGAA AATTTTCTTA AAGCTAACCC AATGGTGACA CCCATCCATA TCCAGCCTGA GTGATACTTT CTGTTCGCCT

Ls AGTATACCTT ACATTTTAGG AGACCCAGAT AACTTTACTC CAGCAAATCC ACTATCCACC CCTGTTCACA TTCAACCAGA ATGATATTTT TTATTTGCTT

Bm TTAAAACCAG AAAGATTGAT GGATCCAGAC AATTTTACTC CTGCCAACCC TATATCTACT CCCCAACACA TTCAACCTGA ATGATATTTC CTATTCGCTT

Cb GTTTTTCATG ATGTATTAAT AGATCCTGAT AATTTTATTC CTGCAAATCC AATATCTACT CCTCCTCATA TTCAACCAGA ATGATATTTT TTATTTGCCT

Cs AAATTTCATG ATTTATTGAT AGATCCGGAC AATTTTGTTC CTGCTAATCC TATGTCCACT CCTCCTCATA TTCAGCCGGA GTGATATTTT TTATTTGCTT

Hm ACAGAGCCTT ATAAGTTTAT AGACCCAGAT AATTTCATTT TAGCAAACTC TATAGTTACT CCAGTTCACA TTCAACCAGA ATGATACTTT TTATTTGCTT

Ib TTTCTCCCTG ATGTATTCAT AGATCCTGAT AATTTTTCTG TAGCAAATCC TATATCAACT CCTGCCCATA TTCAACCGGA GTGATATTTT TTATTTGCTT

Pc TATTCGCCGG ACTTCTTCAT AGACCCGGAT AATTTTGTTG AATCAAACCC GATAATTACA CCTCCACATA TTCAACCAGA GTGGTACTTT CTATTTGCAT

Ph TATTCGCCGG ACTTCTTCAT GGACCCGGAT AATTTTGTTG AATCAAACCC GATAATTACA CCTCCACACA TCCAGCCAGA GTGGTACTTT CTATTTGCAT

Pp TATTCGCCTG ACCTATTTAT GGATCCTGAT AATTTTATAG AGGCTAACCC TTTAGTGACT CCTCCGCATA TTCAGCCAGA GTGATATTTC CTCTTTGCTT

Dm ATTAGACCAA ATTTATTGGG AGACCCTGAT AACTTTATTC CAGCAACTCC TTTAGTAACA CCTGCCCATA TTCAACCAGA ATGATATTTT TTATTTGCTT

Ld ATGCTATTTT ACGTTCTATT CCCAATAAAA TGGGAGGGGT GGTTATATTG TTATTATCAT TATTTATTAT TTTATTGATC ATCATTTTTT GATTTCATGT

Lb ATGCAATCTT ACGATCTGTT CCTAATAAAC TTGGGGGGGT GTTAATGTTA GCTTTATCCA TCATTATTAT TTTAATTAAA TGACTTCTTT ACTTCCACTT

Ls ATGCTATTTT ACGATCAATC CCCAATAAAT TAGGAGGTGT TTTAGCTTTA TTATTTTCAA TTTTAATTTT ATATATTAAA ATTTTATTCT GATCATTCAC

Bm ATACCATTCT TCGATCGATT TCATCAAAGT TTGGGGGAGT AATAGCTTTG GTGTTTTCAA TTCTAATTTT AATATTCAAA TTTTTTTTTT GAATTCAAGT

Cb ATACTATTCT TCGCTCTGTT CCTTCTAAGT TAGGAGGGGT AGTGGCTTTG GTTTTTTCTA TTTTATTTCT GGTTTTTAAA TTTTTGGTGG TTGTTTTGTT

Cs ATTCGATTCT TCGTTCTATT CCTTCTAAGT TTGGAGGGGT GATGGCTTTA GTGTTTTCTA TTTTGTTTTT AGTTTTTAAG TTTTTTGTCG TAGTATTATT

Hm ATTCTATTTT ACGGGCTGTT CCTAATAAAT TAGGAGGGGT TATTGGTTTA TTAATGTCTA TTCTAGTATT AGCATTAAAA TCATTCTGTT GAGTTCAATT

Ib ACGCAATTCT TCGGTCTATT CCTACTAAAT TAGGAGGGGT AGTAGCATTA GTATTTTCTA TTGTAATTTT ATTTATTAAA ATAATTGTTT TAATACAAGT

Pc ATGCAATTTT ACGGAGTGTA CCTAACAAGT TAGGAGGAGT TGTAGCTTTG CTTCTAAGAA TTTTATCTCT GTCTTTAATG ATTTTAACTT ATTCTTTTAC

Ph ATGCAATTTT ACGGAGTGTA CCTAACAAGT TAGGAGGAGT TGTAGCTTTG CTTCTAAGAA TTTTATCTCT GTCTTTTATG ATTTTAACTT ATTCTTTTAC

Pp ATGCCATTTT ACGGGCTGTG CCTAGAAAGC TTGGCGGGGT AGTTGCTTTA GTAATGAGCA TTGTATCTTT GGTAGTTAAA GTACTAGTTT ACTCTCTGGT

Dm ATGCTATTTT ACGATCTATT CCAAATAAAT TAGGAGGAGT TATTGCATTA GTTTTATCAA TTGCAATTTT AATAATCCAA GTAATATTCT GATCTATATT

Ld GGGCACATTT ATCATTTTAA CTTGATTAGG TAGAATGCCT GTAGATTCCC CTTTTAGAAA ATTTTACACT GTGATTTATT TTTGTTTTTA TTTACAAGTT

Lb TGGATCATTC TCTATTTTAA CATGACTGGG CATGCAACCT GTTGAAGATC CCTTCGGTAA AGTTTACTCA GTTCTTTATT TCATTTTTTA CTTCAATTTA

Ls AACAATTTTT ATTTTATTAA CGTGGGCAGG AGCTAAACCA GTCGAAGACC CATTTAGTCA AATTTTGACA GTATTATACT TTTCATTTTT TATTAATTTT

Bm TTCAAACTTT ATTCTTTTAA CCTGATTAGG TTCCATACCT GTAGAACAAC CATATGGACA AATAGTTTCA ATTAGCTATT TTTCTGTATT TCTAGAATCA

Cb TGTAGTATTT TTTTTGTTAA CATGAATTGG TTCTATACCT GTAGAATATC CTTATGGTAA GGTATTATCT GTTTTTTATT TTTTTGTTTT AATAGAGGTA

Cs TGTTATTTTT TTTTTATTAA CTTGAATTGG TTCAATACCA GTAGAGTTTC CTTATGGGAA GGTATTATCT ATAATTTATT TTTTTGTATT AATTGAGGTT

Hm CACTATTTTT ATATTATTAA CATGAACTGG AAGATTGCCA GTAGAATCTC CTTTTGGACA ATGTTTATCA GTAATATATT TTTTAAATAT ATTTCAACAT

Ib TTCTAATTTT CTTTTATTAA CGTGATTAGG AGCCATACCA GTAGAATTTC CATTTAGAAA AATTTTTTCT TCCATGTATT TTATTTTTAT AATTCAATTC

Pc GAGAGTTTTT GTTATGCTCT CATGACTTGG CTCTCTTCCT GCTGAGTATC CTTTTAGTCA AGTTGTAAGA GTAATTTATT TCATTCAAGT AATTCAGTCA

Ph GAGAGTTTTT GTTATGCTCT CATGACTTGG CTCTCTTCCT GCTGAGTATC CTTTTAGTCA AGTTGTAAGA GTAATTTATT TCATTCAAGT AATTCAGTCA

Pp AGTTAGAGTG CTTATTCTCT CTTGACTAGG AGCTATACCT GCTGAAGTGC CATTTAGACA AGTTGCAAGG GTAATTTACT TTACTCTAAT CTTACAAGGG

Dm AGTAACAGTA ATTTTATTAA CTTGAATTGG AGCTCGACCA GTTGAAGAAC CTTATGGACA AATTTTAACT GTTGTATATT TCTTATATTA TTTATCATTA

Ld GTTATTTTCT TATTATTAAT TGTAGCTTTA TTTACGTTGT TCGAACGTAA AGTTTTAGGT TTTGCACAAA ATCGTAAAGG ACCAGATAAA GTAGGTAATA

Lb ATCTTACATA TCCTTTTAAG AGTAGCTCTG TTTACACTAT TTGAACGTAA AATTCTAGGG CTCATTCAAC TTCGTAAAGG GCCTTGTAAA GTAGGGCCTT

Ls ATTAATTTTA TCCTTGTAGG AGTAGCATTT TTAACTTTAT TGGAACGAAA AGTATTAGGA TATATTCAAA TTCGTAAAGG CCCTAATAAA TTAGGTTTTA

Bm ATTCAACTTA TTCTTTTATC TGTTGCTTTT TTTTCACTTT TCGAACGGAA AGTGTTGGCT ATCATTCAAA ATCGAGTCGG TCCGGACAAA GTTGGGATCG

Cb GTAGAAGTTT TATTATTAAG AGTAGCTTTT TTTTCTTTGT ATGAGCGTAA GTTGATAGGG TTAGTGCAGG GTCGAAAGGG TCCAAATAAA GTGGGGGTGG

Cs TTTGAAATTT TGATGATAAG GGTGGCTTTT TTCTCTTTAT ATGAACGGAA GTTGATAGGG TTAATTCAAA ATCGAAAAGG TCCAAATAAG GTTGGGTTAG

Hm TTTATCCTTG TTCTTCTTAC TGTTGCATTT TTTACTTTAT TAGAACGAAA AATTTTAGGC TATATTCATT TCCGAAAAGG TCCAAATAAA GTTTTATTAA

Ib TTGTTAATTA TTTTGTTATC TGTTGCTTTT TTTTCTTTGT TTGAACGAAA AATATTAAGA GTAATTCAAT TTCGAAAAGG TCCAAATAAA GTAGGGTTAA

Pc GTATTTATTG TTTTAATTTG TGTTGCTTAT TTCTCCTTGT TTGAACGTAA ACTTCTTAGG TTAGAGCAAA TCCGGCTTGG ACCAAATAAA GTAGGACCAA

Ph GTATTTATTG TTTTAATTTG TGTTGCTTAT TTCTCCTTGT TTGAACGTAA ACTTCTTAGA TTAGAGCAAA TCCGGCTTGG ACCAAATAAA GTAGGACCAA

Pp TTTATAACAG TTATGGTTTG TGTAGCTTAT TTCTCCTTAT TCGAGCGAAA GATACTAGGT CTAACTCAGC TCCGGCTAGG ACCAAATAAA GTAGGCCCTA

Dm ATTGGTAGTT TATTAGTAAG TGTAGCTTTT TTAACTTTAT TAGAACGAAA AGTTTTAGGA TATATTCAAA TTCGTAAAGG TCCTAATAAA GTTGGTTTAA

Ld AAGGTGTTCT TCAACCGTTT GCTGACGCAG CTAAACTATT TAGTTACCTT TATTCCGCCT TTTTTTTTTT TTTCATTCCT TTAATTCTAT GTATAAGAAA

Lb TAGGCCTTTT ACAACCTTTC TCAGATGCTT TAAAACTTTT TTCATATTAC TTCACTCCTC TGTACTTTCT TATTCTTTCA TTAGTCTTTT TTTTGAATAA

Ls TTGGATTATT ACAACCTTTT AGTGATGCAA TTAAGTTGTT CACTTATTAT TTTTCTCCTG TATTTTTATT TTTTTTGTCA TTAATTTCTT GAATGATTAT

Bm TAGGAATTCT TCAACCTTTT AGTGATGCAA TGAAATTGAT TTCATATTTT TTTTCTCCAG CTATTATATT TGTAATTTCT TTTATTGTTT GAATTACTTT

Cb GGGGGGTTCT TCAACCATTT GCTGATGCTA TAAAATTAAT TAGGTATGCT GTTTCTCCTA TAATTTCATT TTTTATTTCG TTGGTTTTTT GAATTATATA

Cs GAGGGGTAAT ACAACCTTTT GCTGATGCAA TAAAGTTGAT TGGTTATATT GTTTCTCCTA TAGTCTCATT TTTTATTTCT TTGGTGTTTT GAATTTTATA

Hm AAGGAGTTTT GCAACCTATT GTTGATGCTA TAAAATTAAT TACATATTAT ATTTCTCCTA TATTTAGATT TATTATAAGA ATAATTATTT GAATAATTCT

Ib TAGGTTTTTT TCAACCTTTT TCTGATGCAA TTAAATTATT ATTTTACTAT TTTGCACCAA TAGTATTTTT TATTTTGTCA ATTTTAATTT GAATTAGTAT

Pc TTGGTATTCT TCAACCTTTA AGAGATGCTC CCAAATTATT ATCCTTATTC ATTATGCCGT TTATTACATT TATGTTAAGC GTTTCCTGAT GGTACCCGCT

Ph TTGGTATTCT TCAACCTTTA AGAGATGCTC CCAAATTATT ATCCTTATTC ATTATGCCGT TTATTACATT TATGTTAAGC GTTTCCTGAT GGTACCCGCT

Pp CAGGTGTTCT TCAACCATTA AGTGATGCTC CTAAGCTTCT AACTTACGCA TTTGTTACTT TTCTCTCTTT TTTACTTTCT TTAACTTATT GATTTAGTAT

Dm TAGGAATTCC TCAACCTTTT TGTGATGCGA TTAAATTATT TACATATTAT ATTTCTCCAA TTTTTTCTTT ATTTTTATCA TTATTTGTTT GAATATGTAT

Ld AAACTTTAAT TGAGGTTTAT GATATAAATC TTTTTTATTT ATTTTAATAT TATTTTCTTT CAATGTATAT GGAACCATTC TTTCAGGTTG ATCTTCTAAT

Lb ACCATTCTTA TCAACTTCTT TTACTTTATC AATTTTAGTT TTACTCTTTC TATATACCAC CAGGGTTTAC ACTACTTTAG TAACCGGATG ATCTTCTAAC

Ls ACCTTATTTT TGGGTTTTGT TAAGATTAGG TTTATTATTT TTTATGGTAT GTTTAAGTTT GGGGGTTTAT GGTATCATAA TTAGAGGGTG AAGATCTTTT

Bm CCCTTCGAAT TGAGAAATTT TTGATAAGAG GATATTATTT GTTATTGCCT GTATAGGCTC AAGAGTTTAC GGTCTTGTAA TAACTGGTTG ATTTTCTAGG

Cb TCCTGTGATT TGGAATTTTT TAAATTTAAG GATTGTTTTT CTTTTAGTTT TGTTTAGAGT TTCGGTATAT GGGTTTATTC TTTCTGGGTG ATTTAGTTCT

Cs CCCTGTGGTT TGGAATTTTG TTAATTTTGG AATTTTTTTT CTTTTAGTAA TGTTTAGTGT TTCTGTATAT GGATTTATTA TAACTGGATG ATTTAGGTCT

Hm TCCTTTACAA TTTATTATTT GAGTTAATAG ATTTTTAATT TTATTTATAT TACTAGGAAT AGGAGTATAT AGAATATTTT TGTCAGGTTG ATCTTCAAAT

Ib TCCCAGAAAG TGGAATTTAT TCTCTTCTAG TTTCATTTTC GTTATATTTC TCTACGGAAT TCCCATTTAT AGAATAATTT TTATAAGATG AATTTCTAAT

Pc CTATTTTCCA AAAACTTTAT CAAATAACTC ACTTTTAATC CTTATTTTTA TCTCAAGAGT AAGAGTTTAT GCATTAATTT TTACAGGGTC ACTGCCAAAA

Ph CTATTTTCCA AAAACTTTAT CAAATAACTC ACTTTTAATC CTTATTTTTA TCTCAAGAGT AAGAGTTTAT GCATTAATTT TTACAGGGTC ACTGCCAAAA

Pp TCCGATTAAG TTTACGCCAA GAGATAACAT GGGTATCTGG ATTATTATCG GCTTGAGCCT GGTTGTTTAT GGTCCTGTTT CTTGTGGTTG ATTCTCGCAC

Dm GCCTTTTTTT GTAAAATTAT TTAATTTGGG TGGTTTATTT TTTTTATGTT GTACTAGATT GGGGGTTTAT ACTGTTATAG TAGCTGGTTG GTCGTCTAAT

Ld TCTAAATTTG GTAGGATTCG AAGAGTAGCT CAGACTATCT CATATGAAAT AGTTGCTCTA TCTAGATATA CTATATATAC TAAAATATAT TTATTTTTTC

Lb TCAAAATATG GGTCTATGCG TAGAATTGCC CAGTCCCTCT CATATGAAAT TACCGCCTTC ATCATGTCTT CAACTTTAAT AAATTTTCTT CAACTATTTT

Ls AGATTATATG GAAGCCTTCG TTCTGTAGCT CAAACAATTT CTTATGAAGT AAGATTAATA TTAATTGGTA ATTATAGATA TTTTTATTAT TTATATATAT

Bm TCTAAGTATG GAAGGGTTCG AGCTATTGGA ATGTCCATTT CCTATGAAAT TATCATATTT CTTCTTGGAA CTATAAGGAT TTCTTTTTGG TTGTTCTTTC

Cb TCTAAATATG GGTGTGCTCG GGCTTTAGCT CAGTCTATTT CTTATGAGGT TGGATGTTTG TTTTTTTCTT GTATTTCTTT AGAGGTTTTT TTTTTTTTCC

Cs TCAAAATATG GATGTGCCCG AGCTTTGGCT CAGTCAATTT CTTATGAAAT TGGGTGTCTA TTTTTTTCTA GTATTTCCTT GAAGGTTTTT TTTTTTTTTG

Hm TCTAAGTATG GAAGACTTCG AGCTGTAAGG CAATCAATTT CTTATGAAAT TTTAATAATA ACAACAAAAG GTATAAGATT AAAATTTTTT TTTTTATTTC

Ib TCTAAATATG GCTCAATTCG TTCTGTAGCA CAATCTATTT CTTATGAGAT TATTATAATA ATAGTATATT CTTCTTCTTA TTTTTATTGA CTTTTGTATC

Pc TCAAAATATG GAAGACTGCG TGCAATTACT CTTTCTATTT CTTTTGAATT AGTTGCTGTA GTTTTTAATT CTTTTAGCGC AACCAATCCT AATATTATCT

Ph TCAAAATATG GAAGACTGCG TGCAATTACT CTTTCTATTT CTTTTGAATT AGTTGCTGTA GTTTTTAATT CTTTTAGCGC AACCAATCCT AATATTATCT

Pp TCTAAGTTTG GTAGAACCCG TGCTATCGCT CAGTGTATTT CGTTTGAACT TGTCTTTAGG TTTTCATCCT GTACTTCCTG TATAGCACCT AACTTACTGA

Dm TCTAATTATG GAGGTTTGCG AGCTGTGGCT CAGACTATTT CTTATGAAGT TAGTATTTTT TTAATTGGAA GTTATAATTT TTTTTATTGA TTTTTAATTA

Ld CCATATGAAT AAGAGTTATT ATTGAAGTTA ACCGCACTCC CTTTGATTTA GCCGAATGTG AATCAGAATT AGTATCAGGA TTTAATGTTG AATATGGGGG

Lb ATTGTCCTCT TTCTATGATT ATTGAAAGTA ACCGAACTCC TTTTGATTTA TCTGAGTGTG AATCAGAGCT AGTCTCTGGG TTCAACGTTG AATTTGGAGG

Ls TTGGTTTTCC TTTATGTTTA GCTGAAGTTA ATCGAACACC TTTTGACTTT GCTGAAGCGG AGTCTGAATT AGTTTCAGGA TTTAATATTG AATATGGAAT

Bm CTTTGTGATT TGTATTTATT GCCGAAAGTG GTCGAGCTCC TTTTGATCTC TCCGAAGGAG AGAGAGAGTT AGTGTCAGGA TACTCTGTTG AGTATGGAGG

Cb CTTTTTCCTT TTTATTTGTT GCGGAAACAA ACCGTCCCCC TTTTGATTTA GCGGAGGGAG AGAGTGAATT AAAAAAGGGG TTTTGTGTAA AGAAGGGGGA

Cs GTTTTTTTTC CTTATTGGTT GCCAAAACTA ATCGTTCTCC ATTTGATTTA CCTAAGGGTG AAAAAAAGTT GGTAAGTGGA TTTTGTGTGG AGTATGGGGG

Hm CTTTTTTTAT TGCATATTTA GCAGAATTAA ATCGATCACC TTTTGACTTA TCTGAAGGAG AGAGAGAATT AGTAGCAGGC TATACAGTAG AATATGGAGG

Ib CTTGTTTTCC TATTTTTTTT GCTGAAAGTA ATCGTTCTCC ATTTGATCTA ACAGAAGGAG AAAGAGAGTT AGTATCGGGG ATTTTTGTTG AATTGGGAGG

Pc CAATAATTGT AGTTGGAATT GCTGAATGCG GACGAACTCC TTTTGATTTA CCAGAAAGAG AGTCTGAATT AGTAAGAGGT TTTAACGTAG AGTACGGAGG

Ph CAATAATTGT AGTTGGAATT GCTGAATGCG GACGAACTCC TTTTGATTTA CCAGAAAGAG AGTCTGAATT AGTAAGAGGT TTTAACGTAG AGTACGGAGG

Pp CAGCCCCGCT ACTAGCTTTC GCTGAAAGAG GTCGTAGCCC ATTTGATTTA CCTGAAGGTG AAAGGGAATT GGTAAGAGGG TTTAATGTGG AGTACGGAGG

Dm TTTTATTTCC TATAGCTTTA GCTGAAACTA ATCGGAATCC TTTTGATTTT GCTGAAGGAG AATCAGAATT AGTTTCAGGA TTTAATGTAG AATATAGAAG

Ld GATTGAGTTC TCTTTAATTT TTTTAGGTGA AAATTTAATA GTTGTTATTT TTATTTTTTA TTTTATTTGA ATTCGAGCTT CATTTCCTCG ATTTCGATTT

Lb GGCTGAATTC TCTTTAATTT TTTTAGGGGA AAATTTAATA TTAGTTGTGA TTATTTTCAT CAAAGTCTCT ATCCGAGGTG CATACCCACG CTACCGATTA

Ls AGGTGGATTT GCATTAATTT TTTTAGCTGA ATATTCTAGA ATTTTTTTTG TTTCTTTTTT TTTTATTTGA GTACGAGGTA GATTACCACG ATTTCGTTAT

Bm TATTTCATAC ACTTTAATTT TTCTATCAGA AAATAGATCT ATTATTGGCT TAATTTTTCT TGTCGTTTGA ATTCGTGGGA CGGTGCCACG AATGCGATTT

Cb AATAAGGAAT CCTTTAATTT TTTTAGGGGA AAATTTATCT GTTTTGTTAT GGGGAGGCTT TTTGTTTTTG TTCGTTCTAC TCTACCTCGA TTTCGGTATG

Cs AATAAGCTAT ACTTTTATTT TTTTAGGGGA GAATATGTCT GTTTTAATAT TATTAAGTTT TTTTGTGATT GTTCGTTCTG CTTTACCTCG GTTTCGATTT

Hm AATTATATAC ACAATAATTT TTTTAAGAGA AAATATTATA ATTATAATAA TAATTTATTT AGTTTGTTTA ATTCGAGGAA TTCTTCCACG AATTCGATAT

Ib AGTTTGATAT ATTTTAATTT TTTTAGGAGA AAACTTGTAT TTGTTAATTA TTACTATATT AATAGTTTGA ATCCGAGGAA CTGTTCCTCG GATTCGCTAT

Pc TAGGCGTTAT GTTTTACTTT ATTTAAGAGA AAGATTGTTA CTTACCACAT GAATTAGAAT TTCTATTGTT ATACGAGCTA GAGCTCCTCG CATTCGGTAC

Ph TAGGCGTTAT GTTTTACTTT ATTTAAGAGA AAGATTGTTA CTTACCACAT GAATTAGAAT TTCTATTGTT ATACGAGCTA GAAACCCTCG CATTCGGTAC

Pp TGTCCGGTAC ATTTTAATTT ATCTGAGAGA AAGTGTGACT CTTATGATTT GAGTGGTTAT GAGTATCCTT CTACGATCTA GATTACCTCG GATGCGGTTT

Dm AGGGGGTTTG GCTTTAATTT TTATAGCTGA ATATGCGAGA ATTTTATTTA TTTCTTTTGT TTTTATTTGA GTTCGAGGAA CTTTACCTCG ATTTCGTTAT

Ld GACAAAATAA TGTATTTATG TTGATTAATC CTTGTCCCTT TAAGAGTATC AATACCTTGA TTAATTTTCA TAATATTAGG TATTATAGTG GGAATTTCAT

Lb GATAGAATGA TGGAATTATG CTGATTGATC TACCTCCCCC TAACCATTGT TCTTCTAAGT TCTCTTTTCC TACTTCTTAA CATTATTTTA CTCTCCTCAT

Ls GATAAGTTGA TAAATTTATG TTGAAAAAGA TTTTTATCTT TATCTCTTTT TATTTTAAGT TATCATTTCT GTTTTTTAGG AACAATCATT TCTATTTCTG

Bm GATCAATTAA TAATAATGTG CTGAGTTAAA ATTCTTCCTA TTATGTTATT TATTTTTGGA TTAGTTATAA TCTATATTTC ATCTTACGGA CTCTTTATAT

Cb ATAAATTGAC GTCTATATTT TGGATTGAAG TATTGCCTTT ACAATTGTTC TTTTCAGTTC TTTTTCTTAT TTTTTTTTAG AGTTGTGGTA ACATTAAGTT

Cs GATAAAGTAA TGATAATATT TTGAATTGAG GTTTTACCTT TGCAAATGTT TTTTTGTGTG TTATTTTTGG TTTTATTAAG GGTTTTAATT ACGTTTAGTT

Hm GACCATTTAA TAATATTTTG TTGAAAAATT ATGTTACCTT TAATAGTAAT TTTTGTCAAT TTAACTTATT TCGTATTAAC TTTAATTTTA ATTTATTCAA

Ib GATAAAATAA TAGATTTGTG TTGAATTAGA ATAATACCAA TCTGTATATC TTTTATTTCT ATTTTTTTTT TAGTTTTGAC TTTGTTTATT ATTTTTAGTT

Pc GATAAATGCA TAATATTCGG GTGAGAATTT TCAATTCCTT TAATCTTAAT ATTTATGAGA ATTTATGTAC TTTTATTTAG TTGCTTATTC TCTGTGAGCT

Ph GATAAATGCA TAATATTCGG GTGAGAATTT TCAATTCCTT TAATCTTAAT ATTTATGAGA ATTTATGTAC TTTTATTTAG TTGCTTATTC TCTGTGAGCT

Pp GACCATTGTA TAGCGCTAGG GTGAGTAACT TTATTATCTT CTGCTATCTC TTATGCTTTA GCTATTATTT TGATTGTTTC TTCATTTCTG TCACTATCTT

Dm GATAAATTAA TATATTTAGC TTGAAAATGT TTTTTATCTT TTTCTTTAAA TTATTTATTA TTTTTTATTG GGATTATTGG GACATTAATT ACAGTTACAT

Ld CAGAAGCTTG ATTAGGCGCA TGGTTCGGGA TAGAGTTTTT TTTACCATTG TATTACCTAA TACAAGCTTT AGGATCAGTT TTTTTTATTT ATAGTTTTTT

Lb CTCTAGACTG AATCATTTTA TGGGTAGGTC TTGAAATCTT TTTAAGAATC TATTTTATTG TTCAATTCTT TGGATCTAGG GTTTTTTTAA TTGGATGACT

Ls CAAACTCAAT TTTTATAAGA TGAATAGGAT TAGAATCTTT TATTCCTATA TATTTTTTAA TTCAAGCTAT AGGATCTAGA TTATTTTTAT TTTCTTCATT

Bm CAAGATCTTT TCTCATATTT TGAGTTTTGA TGGAATTTTT TATTTCTATC TATTTTTCTA TTCAAGCTGT GGGATCGGCT TGACTTGTTC TTGGAATTTA

Cb CTTCCAGGTG GTTAGTATGT TGAATAATAA TGGAATTTTT TATTCCGATT TATTTTTTTG TTCAATCCAT TGGTTCATCC GTTTTTCTTT TTTCTATCAT

Cs CCTCTAGATG ATTGGTTTGT TGAATAATAA TAGAGTTTTT TGTTCCCATA TATTTTGTAG TCCAGTCGAT TGGATCTTCG ATTTTTTTTT TCTCTGTCCT

Hm TAAGAAATTT TTGAATTTTT TGATTAAGTA TAGAATTATT CTCATCTTTA TATATTCTAT TTCAAAGAAT TCCATCTTCT TTATTTTTAA TGCTAAATTT

Ib GTACTAACTG AGTAATTATA TGAGTAGGTA TGGAATTTTT TATTTCCTTG TATTTTCTTA TTCAGAGCAT TTCATCAGCT TTAATATTTT TGTCATTTTT

Pc CAATGTCTTG GTGACCTGCT TGACTTGGTT TGGAGTGGTT TTTCCCGCTT ATATTTTCCC TTAGTTGCTT AAGGTCAATA GGTTTACTTC TTAGTTTAAT

Ph CAATGTCTTG GTGACCTGCT TGACTTGGTT TGGAGTGGTT TTTCCCGCTT ATATTTTCCC TTAGTTGCTT AAGGTCAATA GGTTTACTTC TTAGTTTAAT

Pp CTTTGACATG ATGGTGTGCT TGGTTGAGAA TAGAATGACT TTACCCCATT TTTTTCTTGT TTCAGTCTGT TAGTTCAGTC CTGTTGTTTT TGTGCTTTAT

Dm CTAATTCTTG GTTAGGAGCT TGAATAGGTT TAGAATCTTT TATCCCCCTA TATTTTTTAA CCCAAGTTTT AGCTTCAACT GTTTTATTAT TTTCTTCAAT

Ld ATACATCATT ATAAAAATTG GGGTTTCCCC TTTTTTTTTT TGAATTTATC CATCTTTTAA ATGAATCCCC AACAAACCTT TAATGATGTT TATAACTTTA

Lb ACTTTTTATA ATTAAACTTA TACTCTTGCC ATTTTTAAAT TGAGTCCTAG AAAGCTTAAA ATGAATTTCC TCGAAACTTC AACTTATTTT TCTCTCAATT

Ls AATTATAATA TTAAAATTAG GATCTTCACC TTTTCATAAT TGATTTATTA ATATTATTGA AGGGATATCA TGAATAACAT GCTTTTTAAT CTTAACTTGA

Bm TGTTGTTTTA ATAAAACTAG GGATAATCCC ATTCCATGGT TGGGCTATGG AATTAGCTGA AGGTTTGAGT GGAAAAAATC TTAATCTATT TCTTACTCTT

Cb TACTTGTATA TTTAAATTAG GGGTATTTCC TTTTATAGGA TGAATAGTTC AGATTAGGGA GAATTTGTCA TGAAGGAAGT TTTTTTTGTT ATCTTCAATT

Cs AACTTGTATA ATTAAATTAG GAGTATTTCC GTTCAGAGGG TGGATGATTC ATATTAGAGA GAATTTGTCA TGAAGAAAAT TCTTTTTGTT ATCCTCAATT

Hm AGTATTTACA ATAAAAATCG GAATTTATCC TTTTCATTGA TGATTTTTTA ATTTAGTAGA AAATTTAAAT TGAATAAGAT TTTTTTTCTT TAATTCTATT

Ib AATAACTATA ATTAAATTGG GAATACCTCC ATTCCATAGT TGAATATTTA AGATTTGTGA AAATATGGAG TGAGATATGT TTTTTTTATT TAATACTATT

Pc GTCCTTTTCT ATAAAAATAG GACTTCCTCC ATTTCATAAT TGATATTTAG ATGTGGGAGA TAGTTTGGAT TCTTGAAGTT TTATAGTTCT TATAACAGCT

Ph GTCCTTTTCT ATAAAAATAG GACTTCCTCC ATTTCATAAT TGATATTTAG ATGTGGGAGA TAGTTTGGAT TCTTGAAGTT TTATAGTTCT TATAACAGCT

Pp TTCATTGTTG CTTAAAATAG GGTTTCCTCC CTTTCATAGT TGGTACCTTG AGCTATGTGA TTCTGTCAGT TCGACTGGGT TTATCCTTAT TAACACTGTT

Dm TATTATATTA TTAAAAAGTG GAGCCGCTCC TTTCCATTTT TGATTTCCTA ATATAATAGA AGGTTTAACA TGAATAAATG CTTTAATATT AATAACTTGA

Ld CAAAAAATTC TTCCACTATA CTTATTAATT TTTTTCTTTG GAGTAATTGA TATTGTTTTA TTTTCTTCTA TTTATCAAAT AAGATGAGTG ATTTTGTCTC

Lb CAAAAAATCG GGCCCTTTTT TTTAAAAACA TTTCTAATTA TAGGAATAGA TCTTTTAGTT ATTTCATCAA ACGGACAATC CGTTTTAATT ATTATCTTAC

Ls CAAAAAATTG CACCGTTGTG TTTAATAATA TTTATCATTG GGGGCCGTAA AATTTTAGCT TTTTCTTCCA TTAATCATTT AGGATGAATA ATTTCGAGAA

Bm ACTAAAATTG GGCCAATATT AGCTGTCATG TTGGTTGTTA TGGCTCGACA GTTTTTAGTA TTAAGATCAA TTGTAAATCT TTGTTGAATT ATACTCTCTT

Cb CAGGAAGTAA TTCCATTATT AATATTTTTT TTTGTTTGTT TTTGTCACTG GATAATAATA GTAAGATCGT TATTTAATGT TGGTTGAATA GTTGTAGCTG

Cs CAGGAAGTGA TTCCTTTATT AATATTTTTT TTTTTAGTTT TTTGTCACTG GATTATAATG GTTAGATCTT TGTTTAATGT TGGTTGAATA GTAGTGGCTT

Hm GTTAAACTTG TTCCAACTTT AGTATTAATT TTTTTTTTAT TCAGACGAAT GATTTTATCT TTTTCTAGGA TAATTCATTT TTCTTGAATT ATTTTATGCT

Ib CAAAAGATTA GGCCTATGAT TACAGTAATA TATTTTGTTT TATGTCGTCG ATTTATCTTG TTTAGTTCAA TTTTGAATAC AAATTGGATG TTAATATGTA

Pc TTTAAGGTTC CTTCTCTTGT TTTTTTAATA TTATACGCAG CTGGGCGTCG CATGTTAATT TATTCTTCTA TTTTAAATGT TGTTTGAAGC TTAGTTTCTT

Ph TTTAAGGTTC CTTCTCTTGT TTTTTTAATA TTATACGCAG CTGGGCGTCG CATGTTAATT TATTCTTCTA TTTTAAATGT TGTTTGAAGC TTAGTTTCTT

Pp GTTAAACTTC CTCCTCTCTG GCTACTTATG GTTCTGTGTT CAGGGCGACG AATAATAATT TACTCTTCGT TTGTTAATTA CGTGTGAGGA TTAGCATCAA

Dm CAAAAAATTG CACCTTTAAT ATTATTAATT AGAGTAATTG GAGGACGAAA ATTAATAGCA TTTTCTTCAA TTAATCATTT AGGATGAATA TTAAGATCTT

Ld TAATTATATC TAAATATATA TTCAAATTTT ATTTCATATA TTATATTATA TTTTCTATAG TCGCAATAAT TATATGGTTT ATTTTTTTTC TTATTAGGGG

Lb TTCTCTTTTC TAAGGCCATT GGACTAATTT ATCTTTTTGT TTATATAACC TTTTTTTTAA TCTTTTATGA AACCATTTTT ATATTCATTT TTATTAGGGG

Ls TAATTTTATC TAAATATATT TTAATTTTAT ATTTTTTAAT TTATTCATTC ACATTAATTA TAATTTTTTT TTTATTTTTA TCTTTACTAT CATTAGGAGG

Bm CTGCTATAAG AATGTATTTG AGAGTAATAA TAATATTATT TTACTTTATC GCAGTACTGA CAATTTTCGT ATTTATTGTG GCAATAATTT CATTGTGAGG

Cb CTTTATTAAG AAAAGAAGGA TTGATTTTCT TTTTTTTTGT TTATTTTAGA GTTTTTTTTT GGGTATTAGT GTTTCTTTTT TTTGTTTTTA TAATAATAGG

Cs CTTTATTAAG TAAAGGGAGA TTGGTGTTTT TTATAACAAT TTATTTTAGA GTTTTTTTTT GAATTTTAGT TTTTCTTTTT GTAGTTTTTA TGATAATAGG

Hm CTATTTTAAG AGTAAGATAT TTTCAATGAT ATATTTTAAT TTATTTTTTA ATGTTAATTA ATGTTCTATA TATTCTATTT TTACTTTTTT CTTTTATAGG

Ib TAATAGCGAG AAAATTTGTG TTTTTGGAGT ATTTAGTTTT TTATACATTT TGTTTATGCA AATTATTAAT AACAATACTT TGTATTTTAA CCATTTGTGG

Pc TATTTTACTC GCTATCTTTA TTTTTAACTT TCTATTGTAT TTATTTTAAT TGTGTGTTTT GATTTCTAAT TTTAGGTTTA AGGCTTGCTA ATTTAGCAGG

Ph TATTTTACTC GCTATCTTTA TTTTTAACTT TCTATTGTAT TTATTTTAAT TGTGTGTTTT GATTACTAAT TTTAGGTTTA AGGCTTGCTA ATTTAGCAGG

Pp TAGGATTGAG TGTAAGAGCA TTCGTGACAT TTTTTCTGAA TTACTTTATC TTGACGTTTT GGTTTATGGT TTTTATAGCT TCTTGGCTAA GGCTATCAGG

Dm TAATAATTAG AGAATCAATT TGATTAATTT TATTTTTTTT TTATTCATTT GTATTAACAT TTATATTTAC ATTATTTATA AATTTTTTAT CATTAGGAGG

Ld CTTCCCACCT TCAATTATTT TCTTGATAAA AATTCATATT ATTATAAATA TTATAATTTT ATTTAATTTA TTTATGATTA ATTATCTTAA ATTAGGATGG

Lb GTTTCCGCCT TCATTGATCT TTTTTGCCAA ACTAAACTTC CTCTTAATAT CCGGGATTAT TATTTTCACT ATATTTTTTG TTTATATAAA AGTTTATTTG

Ls ATTACCACCT CTTCTAGGAT TTTTTCCTAA ATGATTAATT ATTGAAAAAA TATTAAATTT TATTTTAATC ACTATTTTTT ATTATTTACA AATATATTCG

Bm TATTCCTCCA TTTATAATAT TTATAGTAAA ATATTCTACA ATAGAAATAT TACCGTGTTT AATTATGCTC CTTCTTATTA CATATTTATC AATATAATGA

Cb AATTCCTCCA TTAGGGAATT TTCTTGGGAA GGTAGAAGTG TTAACTAGAT GAATAAGGGT TTATTTTGTG TTTATATTTT TGTATTTAAA GTGGTGTTTG

Cs AGTTCCTCCT TTGGGGAATT TTCTTGGGAA AGTAGAGATT GTCTCTAGAT GGTTGAGTTT TTTTTTTTTA TTTATGTTTT TATATTTGAA GTGGATTTTT

Hm AATTCCTCCT TTATTAGGTT TTTTAATAAA AATGTTTGTA GTTTATGGTT TACTAAGTTT ATTATTAATA CCAATAATTT TTTATTGAAA AATCTTTCTA

Ib TTTACCTCCT TTTTTAGGTT TCTTTATCAA AGTAGAGCTC ATTAATTATT TGTTGGTTTT TATAAATATA ATAATATATA TATATTTACA CATTTTTTGA

Pc TATACCGCCT TTAAGAATAT TTTTCATAAA GATAAAAATT ATCTCATCTG TTCTTACTAC TGCAAGATTA ATGTTGTGAA TTTATATGAG GCCCTAGTTT

Ph TATACCACCT TTAAGAATAT TTTTCATAAA GATAAAAATT ATCTCATCTG TTCTTACTAC TGCAAGATTA ATGTTGTGAA TTTATATGAG GCCCTAGTTT

Pp TCTTCCACCT TTTGGGTTGT TTTTTGCAAA GGCTCTTGTA GCATATGAAC TAACATTATG TCTAGCGTTT ATGATATGAA TTTACCTAAG GCCTTATGGT

Dm ATTACCTCCA TTTTTAGGAT TTTTACCAAA ATGACTTGTA ATTCAACAAT TTATATTAAC AATTATAATA ACATTATTTT TTTATTTACG AATATTTATG

Ld TGTTTTAGTT TATTTGTTTT TGAAGGGATA CTATCTTTCT TTTATATACC TATTTTTTTT TGAATGAATA TCTCTATCCT TTTATTACTT TTTTTTTATT

Lb TTTACTTATC TTTTTATTTT TGATGGGATA GTTTCCTCCT TTTTTATACT TCTTTTTATT TGAGTGAATA TCTCTTTGTT TGTATTTTTC TCTATCTCTA

Ls TACTTACATT TTTTTAATTT TGATGGGTTG ATTTCCTCGT TTTTTATACA TATTTCTATT TGAGTGAATA CTTATTTCCA TTTATTTATT TATTTTTTTC

Bm TATTTGTGTT CCTCTGTTTT TGATGGGATG AATAAATCAT TTTATATGGA TTTTTCTATT TGAATGAGTG TGTATCTCCT TATGCGCATT TATATATTGA

Cb CAGGTGGATG TTTTTAGTTT AGATGGGATG ATTTGATCAT TTTTTGTGGG TTTTTCTTTT TGAGTGATTA TGTTGATCCA TTTTTTTATA TTTGTTTGTT

Cs TTTGCGGATT TATCTAGTTT AGATGGGATG ATTTATTCAT TTTCTATGGG TTTTTCTTTT TGAGTGATTG TGTTGATCCA TTTTTTTATC TTTGTTTGTA

Hm GAGCTATATT TTACTAGGTT TGATGGGTTC GTTTCATCAT TTTAGATGCT TGTTTCTATT TGAGTGATTA TATATTTCCT AATATATTTT TTATTACCTT

Ib TGTATACGTT TGTCTTGTTT TGATGGGATG ATATGTTCAT TTTTTATGGG TTTTTTTGTT TGAATGAATA TATTGATCCA TATATATGTT TATGTTATAT

Pc TACATTTATA TATTTGCTTT AGATGGGGTA TACCAATCAT TTAGTGTAGG TGTTTCTATT TGAGTGATTG TGCACTTCCG TATCCCTATC TACTTTTCTT

Ph TACATTTATA TATTTGCTTT AGATGGGGTA TACCAATCAT TTAGTGTAGG TGTTTCTATT TGAGTGATTG TGCACTTCCG TATCCCTATC TACTTTTCTT

Pp TGTGTGTGTG CGTTTGCTTT TGATGGGGTA TTTTAGTCAT TTATGATAGG TATTTTTGTT TGAGTGAATG TATTTTTCCA TACTTCTCTT TTTATTTTTA

Dm CTTTTATTTC TATGTATTTT TGATGGGTTG ATTTCTTCGT TTTTTATACA TATTTTTATT TGAGTGAATG CTTATCTCCA TTTATTTATT TTTATTTTAC

Ld TGATGAAATC ATCTTGATGA AATTTTTTTT TTATTAATTT CATACATTTT TTTTGATTTA TTCGTGGATA AATATTCTAG TATATTTTCT TTGATTGTAA

Lb TGATGTATAG GTCCTGTTGA TCTTTTATTT TTGCTATCAG CTTTATCGTT GTGTTTGTTT TTTTTTGATG CCTATTCAGC TTCCTTCTCT TCTGTGGTTC

Ls AGATGAACAG GGCTTGATGT CAGATTAATT TTTTTTTTTT TTGGAATTAT TTTAGTAATT TTATTTGACT GAATATCTAT ATTTTTTTTA AGTTTAGTAA

Bm TGAATTTATG GTCTTTATGA ATTTTTTTTT TGTTTCGTAT ATTTAATTAT TTCAACTATC ATATTTGATC AGTTAAGAGT GAGATTCATA TTCATGGTTC

Cb TGAATGTATG GACTTGATGA AGGATTATTT GGTTTTGTTG GGGGATTATT TTTTTTGTTA GTTGTTGATT CATTTTCTAT AATTTTTTTG TTTACTGTAG

Cs TGATTGTATG GACTTAGTGA AAGATTGCTA AGTTTTTTTG GAGGGGTGTT TTTTATTTTA TTATTTGATT TGTTTTCAAT GATATTTCTT TTTACAGTTG

Hm TGATGATGAG GAGTTGATGT ATATATAAGA GGAATAATAT ATATTTTAAT ACATAATTTT ATTTTTGATA AATTTTCTAT AATCTTTTTA TTTATAGTTT

Ib TGATTGCATG GACTTAATGA ATTGATTATA TCTTTTTTCA TGTGAATATT GTATTCAATA ATTGTAGATA AATACTCTTT GATTTTTATA ATTATAGTTA

Pc TGACTCATAG GAGTTGATGA AATTTTATCA CTTTTAATTC AATTTTGTTT TGTTATTATT GTACTGGATA ATTTAAGCCT GACTTTTTTG CTTATAGTAT

Ph TGACTCATAG GAGTTGATGA AATTTTATCA CTTTTAATTC AATTTTGTTT TGTTATTATT GTACTGGATA ATTTAAGCCT GACTTTTTTG CTTATAGTAT

Pp TGAATGGTAG GTCTTGATGA ACTATTGGGC CTATGCCTCC AGTTCCTTTT TTTGGTATTT ATTTTTGATC GGGTATCTGT CATGTTTTTA ATGATGGTGT

Dm AGATGAACAG GATTTAATGT CAGTTTATCA TGTTTTTTAT TAAGTTTATA TTTTACTTTT CTTTTTGATT GAATAAGTTT ATTATTTATA TCTTTTGTTC

Ld ATTTAATTAC TTTATGCATT TTTTTGTATT CTTTAAGATA TATGAGAAAA GAAAAAGATA AAAATATCTT TTTTTATGTT TTATTTTTTT TTTCTTTAAG

Lb TTTTAATCTC AGCATCAATC GTGTTTTATT CCATATCTTA TATACAACAA GAAAAGGAAA AAATCAAGTT TTTTTTAACC CTATATATGT TCATTCTATC

Ls CTTTTATTTC TTGTTTAATT TCAAATTATA GAAATTCCTA TATATTAGGA GATAATAATT CAAAATTGTT TATATTTTTA ATTATTATGT TTGTATTTTC

Bm TCTCAGTTAG AACTTGCGTT CTAATTTATG CTGTGTGATA TATAGAAGGA GAAAAAAATT TTAATAAATT TATTGTAACT TTATTCATAT TCATTATTTC

Cb GAATGGTTAG GAGATTTGTT TTACTTTATT CTAATTATTA TATGATGGGA AGATTGTTTA AAAAGAAGTT TATTTTAGTA ATGATGATTT TTATTCTTTC

Cs GATTGGTGAG AGGTTTTGTT CTTTTATATT CTAACTATTA TATAAAGGAA AGGTTGTTTA AAAAGAAGTT TATTGTAGTA ATGATGGTGT TTATTTTTTC

Hm TAATTATTTC AAAAAATGTG TTAAAATACT CTTATTTTTA TTTTGTTGGG ACTGTATGAA CTTTACGATT TATTGGAATT TTAATTTTTT TTATTGTATC

Ib CAATAATCAG AACCATTGTA ATAATTTATT CTATCTATTA TATAATAGAA GAAAAAATGA AAAAAAAGTT CTTTTTATCG ATATTTTTTT TTATTCTGTC

Pc TAACAATTAG GTCGCTTGTT ATAGCTTATA GAAACTATTA TATAGCAGGT CATAATCTTG GCGGTGATTT TTACGTTTCC ATGGTCTTAT TTATTGTAAG

Ph TAACAATTAG GTCGCTTGTT ATAGCTTATA GAAACAATTA TATAGCAGGT CATAATCTTG GCGGTGATTT TTACGTTTCC ATGGTCTTAT TTATTGTAAG

Pp TAGTCGTTAG GGCAGTGGTG ATAGTTTTTG GTAGATTCTA TATGACTTCT TCTTTACATG GTGTTGCTTT TACATTTAGG ATATTGATTT TCATCGCCAG

Dm TTATAATTTC TTCTTTAGTG ATTTTTTATA GAAAAGAATA CATAATAAAT GACAATCATA TTAATCGATT CATTATATTA GTATTAATAT TTGTTTTATC

Ld AATGCAAATT TTAATTTTTT CATTTTCTTT CTCTAGAATT TTGTTAGGAT GAGACGGTTT AGGTATAACT TCATTCTATC TAATTATTTT TTATCACAAC

Lb AATGCTAATC TTAATTTTCT CTTTTAATAT TTCTTCCCTT CTAGTCGGTT GAGATGGGTT GGGTGTAACT TCCTTTCTAT TGATTTATTA TTACCACTCA

Ls AATAATATTA ATAATTGTAA GACCTAATAT AATTAGAATT TTATTAGGTT GAGATGGTTT GGGATTAGTA TCTTATATTT TAGTAATTTA TTATCAAAAT

Bm AATAATGTTC TTATGTATGA GAACAGATAT TTATTGAGTT ATAGTAGGTT GGGATGGACT AGGAATCACT TCATTTTTTT TAATTATTTT CTTTCAAAAT

Cb TATATTTGTT TTAAGGTTAA GTGGAGATTT ATTTTGGGTA ATGATTGGAT GAGATGGATT GGGATTTTCT TCTATGTGTT TGATTTTTTT TTTTCAAAAT

Cs AATGTTTTTG TTGAGTATAA GGGGGGATTT GGTTTGGTTG ATGGTTGGTT GGGATGGGCT TGGTTTTTCT TCAATGTGTT TAATTTTTTT TTTTCAAAAT

Hm AATATTATGA TTAATTATAT CTTATGATAT ATTTACTTTT ATTGTAGGAT GAGATATGTT AGGTGTATCT TCTTTTTTAT TAATTTTATA CTATAATTCT

Ib GATAATAATC CTTTCATTTT CTGCTAATAT TTTTTGATTG ATAGTAGGGT GGGATGGTTT AGGTCTCTCT TCATTTATTC TTATTATATA TTTTCAAAAT

Pc AATATTACTT CTATCTCTTA GAGGCTCAAT GTTCTGATCT TTTATTGGGT GGGACGGTTT AGGAATAATA AGTTTAGTTT TAATTTTATT TAATAAAAGA

Ph AATATTACTT CTATCTCTTA GAGGCTCAAT GTTCTGATCT TTTATTGGGT GGGACGGTTT AGGAATAATA AGTTTAGTTT TAATTTTATT TAATAAAAGA

Pp GATGGTTATT TTATCTGTCA CAGGATCTTT GTTTTGGTTA TTTTTAGGGT GAGACGGTTT GGGCTTATCT AGGTTCATTT TAATTATTTT TAACAAGAAT

Dm AATAATATTG TTAATTATTA GACCAAATTT AATTAGAATT TTATTAGGGT GAGATGGTTT AGGACTTGTT TCTTATTGTT TAGTAATTTA TTTTCAAAAT

Ld GTTAAAAGAT TACATGGAAG AATGGTCACC GTATTAACTA ATCGATTAGG TGATTGTTTT GTTCTTTTAG CCTCTCTTAC TAAAAGAGCC CAATTACCTT

Lb TTAAAAAGAA CAAACTCCTC TCTCATTACA TTAACATTAA ATCGGGTTGG AGATTTAATA ATTATAACTG CAGCATTAAG AAAAAGAGCT CAGCTCCCCT

Ls GTAAAATCTT ATAATGCTGG TATATTAACT GTATTATCAA ATCGAATTGG TGATATTATA ATTATTATTG CAGGTATAAC TAAAAGAGCT CAAATTCCTT

Bm TGAAAGAGGG TAAGAAGGGG GATAGTAACT CTCCTATCTA ATCGAATTGG AGACGTGTTT ATTGCTCTTG GAGCAATTAC AAAAAGTGCA CAATATCCCT

Cb TGGAAAAGGT TTAATAGTTC AATGGTAACT TTTATTTCTA ATCGAATTGG GGATTTTTTG ATTTGTTTTG GTGCTTTGAG AAAGAGTGCT CAAGTTCCAT

Cs TGGAAGAGGT TTAATAGTGC GATGGTTACT TTTATTTCAA ATCGAATTGG GGATTTTTTT ATTTGTTTTG GGGCGTTTAG GAAGAGGGCT CAGGTTCCAT

Hm TATAAATCGA AAAAAAGAAG ATTAATTACT TATATTAGAA ATCGGTTTGG TGATGGATTT TTTTTTTGTA CTAGAATTAC AAAAAGAGCT CAATTTCCAT

Ib TGAAATAGAT TCAATAGATC TATAACTACA TTTATATGTA ATCGATTTGG TGACTTGTTT ATAATTGTTT GTGCTATAAC TAAAAGTGCT CAAGTTCCCT

Pc TGGAGTTCTC AAAAATCGGG AGTGATTACT TTCCTAATGA ACCGATTAGG TGATTCTTTT ATATTAATTG GAGGGGCTTC AAAAAGAGCT CAATTTCCAT

Ph TGGAGTTCTC AAAAATCGGG AGTGATTACT TTCCTAATGA ACCGATTAGG TGATTCTTTT ATATTAATTG GAGGGGCTTC AAAAAGAGCT CAATTTCCAT

Pp TGATCTTCCT CTAAATCAGG GTTAATTACT TTTTTAATAA ACCGGTTAGG GGATGTGTTG ATAACTATCG GTTGTTTGTC AAAGAGAGCT CAGTTTCCTC

Dm ATTAAATCTT ATAATGCTGG TATATTAACT GCGTTATCTA ATCGAATTGG GGATGTAGCT TTAATATTAG CTGCTATAAC TAAAAGAGCT CAGATTCCTT

Ld TTTCTTTTTG GCTTCCTAAA GCCATGGCAG CCCCAACTCC TGTATCTTCT CTCGTGCATT CATCCACTTT GGTAACAGCA GGGGTTTATA TTTTATTTCG

Lb TTTCTTCCTG GCTACCTCTA GCTATAGCTG CCCCCACCCC TGTTTCCTCT CTTGTTCACT CTTCAACTCT AGTCACTGCT GGTGTGTATC TCCTCTATCG

Ls TTTCTTCTTG ATTACCGGCT GCTATAGCAG CCCCTACTCC TGTATCTGCA TTGGTTCATT CTTCTACATT AGTTACAGCG GGGGTTTATT TATTAATTCG

Bm ATTCAGCGTG ACTTCCGGAG GCTATAGCAG CTCCCACTCC AGTTTCTGCA TTAGTCCATT CTTCTACGTT AGTAACTGCA GGAATCTACC TCCTTCTTCG

Cb TTTCGGCTTG ACTTCCTTTG GCAATAGCTG CTCCAACTCC TGTTTCTTCT TTGGTTCATT CTTCTACTTT AGTTACGGCA GGGGTTTTTT TATTAATTCG

Cs TCTCTGCTTG GCTTCCTTTA CCGATAGCTG CTCCTACACC TGTTTCTTCG TTGGTTCATT CTTCTACTTT GGTAACGGCT GGAGTCTTTC TATTAATTCG

Hm TTTCTAGATG ACTACCTGAA GCTATAGCAG CCCCAACTCC TGTATCAACT TTAGTACATT CATCTACTCT AGTTACAGCT GGATTTTATT TTTTATTTCG

Ib TTTCTGTTTG ACTCCCTTTA GCCATAGCAG CTCCTACACC TGTATCATCA TTGGTTCATT CTTCTACATT GATTACAGCT GGAGTTTTTT TATGCATTCG

Pc TTTCTAGCTG ATTGCCGGAA GCTATGGCAG CCCCTACTCC CGTGAGTAGG TTAGTTCATT CTTCAACATT AGTAACTGCT GGTATTTATG TTTTAGCTCG

Ph TTTCTAGCTG ATTGCCGGAA GCTATGGCAG CCCCTACTCC CGTGAGTAGG TTAGTTCATT CTTCAACATT AGTAACTGCT GGTATTTATG TTTTAGCTCG

Pp TTTTAAGATG GTTACCCGAG GCTATAGCAG CTCCTACCCC TGTAAGAAGG CTGGTTCACT CTTCAACTCT GGTTACTGCA GGCATTTATA CACTAGTTCG

Dm TTTCTTCTTG GTTACCTGCA GCTATAGCTG CTCCTACACC TGTTTCTGCT TTAGTTCATT CTTCTACATT AGTTACAGCT GGTGTATATT TATTAATTCG

Ld TTTTATTTTG TCTTTCAGAA CTCTTATTAT AGGTGGTTTT ATGGCTATTA AAAGATTTGA TTTGAAAGAA ATTGTTGCTT TCTCGACCCT TAGGCAGGTA

Lb AGCTTTCATT ACGAGCCTCA CGCTAATTAT GAGAAGGGTT TTAGCATTAC AAAGATTCGA CTTAAAGGAA ATTGTCGCCT TTTCAACAAT AAGGCATATC

Ls ATTTTTCATT TCTGGTTTAA CTATATTTAT ATCTGGTTTA GGAGCTAATT TTGAATTTGA TTTAAAAAAA ATTATTGCTT TATCAACTTT AAGTCAATTA

Bm ATTTTCAGTT GCGAGAATGT CAGCTTTTTT GTCAATCTCT AGAAGATGGG GTGAGTTGGA TTTAAAAAAA ATTATTGCTC TATCTACTCT CTCTCACTTG

Cb TTTTTTGGTT AGCTTTATTA CTATTTTTTT GGCTGGGATA AGATCAGTGG GGGAATATGA TTTAAAAAAG GTAATTGCTT TGTCAACTCT TTCTCATATT

Cs TTTTTTTGGG AGTTTTTTGA CATTTGTTCT TGCGGGGATA AGATCATTAA GGGAGTATGA CTTGAAGAAA ATTATTGCTT TGTCAACTTT ATCTCATATG

Hm ATTTTTTATT TCATTGTTTA CGATAACTTT AGCCAGAAGA GCTGCTTTAA TAGAATATGA TTTGAAAAAA GTTATTGCTC TTTCAACTTT AAGACAGATT

Ib ATTTTATATT TCATCTTTTA CTTTTATCAT ATCTGGCCTG TCAGCAATAT ATGAATACGA TTTAAAAAAA ATTATTGCTC TATCAACTCT TTCTCATATA

Pc TTATTACTTA TCTTCTATTT CTATTATTAT TTCGGGTGTT TCAGCTCTCT GAAGGAGAGA TTTAAAGAAA GTTGTAGCTT ATTCAACTCT TTCTCACATT

Ph TTATTACTTA TCTTCTATTT CTATTATTAT TTCGGGTGTT TCAGCTCTCT GAAGGAGAGA TTTAAAGAAA GTTGTAGCTT ATTCAACTCT TTCTCACATT

Pp GTTTGCATTT TCTTTTTTTT CGTTAGTTGT ATCAGGGCTT TCTGCTCTTG TTTCAACGGA CTTAAAGAAG GTAGTAGCAT ATTCTACCCT CTCTCACATT

Dm ATTTTTATTA TCTGGATTAA CAATATTTAT AGCTGGATTA GGAGCTAATT TTGAATTTGA TTTAAAAAAA ATTATTGCTT TATCTACTTT AAGTCAATTA

Ld GGTTTAATAA TATTTTTTAT GTCTTTAAGA TTAAAAGAAG TAGCCATATT TCATTTATTA ACTCACGCTT TATTTAAATC AGTTTTATTT ATATGCAGAG

Lb AGCTTAATGA TAATAGGAAT CTCAAATGGT CTTTATAAAT TTTCATTTTT TCACCTTTGT ACCCATGCTT TATTTAAGGC ACTTTTATTT ATGTGCTCTG

Ls GGATTAATAA TAGGAAGTTT ATCTATAGGA TTAACTAATT TTTGTTTTTT TCATTTATTA AGACATGCTT TATTTAAAGC ATTATTATTT ATATGTGCTG

Bm AGAATGATAA TTTTATTCAT CTCTTGTAAA GATTATCTCT GTGCAATTAT CCACATAATT AGACATGCAT TTTTTAAATC TTCTTTATTC ATATTTGCAG

Cb GGATTAATAA TAATGTTTGT GGGGATGGAG AGATTTATTT CTGCAAAGAT TCATTTAGTA ATTCATGCCT TTTTTAAGTC TCTTTTGTTT ATAATTTCTG

Cs GGTTTAATGA TAAGGTTTGT AGGAATGGAA AGATTTGTTT CTGCTAAAAT TCATTTAATT AATCATGCTT TTTTTAAGTC TCTTTTGATT ATAACTTCTG

Hm AGTTTTATAT TTTTTAGTTT AAGTTTGAAG TTAACAACTT TAGCTTTTTT TCATATAGTT ATACATGCAT TTTTTAAAGC AGCAACTTTT ATAATTGCAG

Ib GCATTGATTT TTTTTTTTTT AAGTATAAAT AGATTTGAGT CTTCTATGAT TCATTTGATT ACTCATGCTA TCTTTAAATC TTCTTTGTTT ATAAGAGCAG

Pc AGCTTAATGC TTTTTTACTT ATCTGAAGGA AGGGTGGAAG GAGCTTTAAT CCACATGTTG ACGCACTCTG TGTTTAAAAG GCTGTTATTT ATAACTTTAG

Ph AGCTTAATGC TTTTTTACTT ATCTGAAGGA AGGGTGGAAG GAGCTTTAAT CCACATGTTG ACGCACTCTG TGTTTAAAAG GCTGTTATTT ATAACTTTAG

Pp AGCCTTATAA TTCACTATCT CTCAGAGGGC TGTATTGACG CTTGTATACT TCATATAGTT ATGCACGCAA TCTTTAAGAG TCTTTTGTTT ATGTCAGTAG

Dm GGTTTAATAA TAAGAATTTT GTCTATAGGA TTTTTAAAAT TAGCTATATT TCATTTATTA ACTCATGCTT TATTTAAAGC ATTATTGTTT ATATGTGCTG

Ld GGTATTTGAT TCAGGATATC AGACCTGTTT TAAAATTATC TATTATTATT AGAGGTTTAC CATTTCTTAG CGGGTTTTAT TCTAAAGACT TATATACTTT

Lb GGTACTTAAT CCAAGATATC AATTATAAAG TAAAATATAT TTTCTTTATC ATGGGGCTTC CATTTATGGC GGGGTTTTAC TCTAAAGACG GGTACACCAT

Ls GATATTTAAT TCAAGATATT CTTCCTTTTG TAAGATTATG TTTTAATGTT TGTGGAATTC CATTTTTATC TGGATTTTAT TCTAAAGATT TATATTCTTT

Bm GAATTCTTAT TCAAGATATT ACTCATTTTC TTTCGATATC ATTAGTATTT GTGGGTGGAC CGTTTTTAGC AGGATTTTAT TCTAAGGAAA TTTATTCTGC

Cb GATTTATTAT TCAGGATATT AATTATTTTT TTTCAATAAC TTTTCTTCTT ATAGGAGCAC CATTTTTTTC TGGATTTTTT TCGAAAGAAA TTTATTCGAT

Cs GATTTATGAT TCAAGATATT AATTTTTTTT TTTCTCTTTC TTTTATTTTG GGTGGGGCTC CTTTTTTTTC TGGGTTTTTT TCCAAAGAAA TTTATTCTAT

Hm GAGTAATTAT TCAGGATTTA AATAAATTAT TATTACTACT ACTATTGTTT TCAGGATTTT TATTTCTTTC AGGGTTTTAT TCAAAAGATT TATATTGTTT

Ib GAGTTTTAAT TCAGGATATT AATATAAAAC TTATTTTCAT AATTATTATT ATAGGAATTT TATTTTTATC AGGATTTTAT TCTAAAGAGT TATATTCTAC

Pc GAAGATCAAT TCAAGATAGA ACCTTTAGAA TAGCATCTTT AGTGGGAGTT GCCGGACTGC CGTTTCTTTC GGGAGGTTAT TCAAAAGAGG TGTATTCGTT

Ph GAAGATCAAT TCAAGATAGA ACCTTTAGAA TAGCATCTTT AGTGGGAGTT GCCGGACTGC CGTTTCTTTC GGGAGGTTAT TCAAAAGAGG TGTATTCGTT

Pp GTAGGGTGAT TCAGGATACC TCAACAGGGC TAACAACTTT TGCTTTACTA GCAGGGCTAC CTTATTTAAG AGGTGGATTC TCTAAAGAGG TTTATTCTGT

Dm GGGCTATTAT TCAAGATATT ATACCTTTAA CTTCAGCTTG TTTTAACGTA TGTGGAATAC CTTTTTTAGC TGGATTCTAT TCTAAGGATA TATATTCATT

Ld ACGACTGATT AATTTTATCA TAGGATCTAT TTTTATAGGC AGTAGTATTC AATGAACAAT TATAATTTTA TATTTTGTTA TTGTAATAAT TATATTTATC

Lb ACGCTTATTA TACTATGCAT GATTTTCTAT TTTTAGTGGA GCAGCAATTC AATGAAGATT AATTATATTA ATTATAAGAA GGCTAATCAA AACTCTTGTA

Ls TCGATTATTT AGTTTTTTTT TGGGATCTTT ATTTGGAGGA AGAATATTAA TATGATTGGT TTTGTTTTTA ATTTTAATTG GTATTTTTAA AATTTATTTA

Bm CCGCATTATT TTCCGCACTA TTCTTAGAAT TTCTGCTGGA AGGATAATCA TCTGACTGAG AAAGTTATTT CTTTTTTTAT CCATATTTAT ATTTCTTTGT

Cb TCGTATGTTA TTTTATAGAG TTGTTTCTTC TTTCTTAGCA GGAGTGATGG GGTTTGTTTT TTCTTCTTTT CGGAAAGTGG GGTTATTAAT TTATTTAAAG

Cs TCCGGCTTTG TATTATAGGA TTGTTTCTTC TTTTTTTAAG GGGTTGATTT TGTTGAAGTT TTCCCTTTTT CAAAAGATAA GAATGAAGGT TTTTGTTAAA

Hm ACGAATATGT TTAATAATAT TACTTTCAAT TGTTTCAGGT TCTGTTTTAA TATGATATTT ATTTATAATT ATAATTCTTA TACCAATAAT AATTTTAGAA

Ib ACGTTTGTTA TATATTAGAA GAAGAATTTC GATTTTTTTA GGAGGATTTC TATTAAAATT ATTTTGATTG ATTCTCTTCT TTATATTTTA CTTTCAACTT

Pc TCGCATTATT TACTTAAACG TTCTAATTTC AGCTTGAATT TCCTCAAGTC CTGGCTACAT ACTCTTTATA ATTCTATTTG GTTTTATTAC AATACCTGCT

Ph TCGCATTATT TACTTAAACG TTCTAATTTC AGCTTGAATT TCCTCAAGTC CTGGCTACAT ACTCTTTATA ATTCTATTTG GTTTTATTAC AATACCTGCT

Pp GCGAATTATG TGGATCAATA TGATGGTGGT ATTCTGAGCA GTCTGAACTC CTTTTTGAAT GATTAGAGTG GCGTCACTAG GAGGCTGTAC AATTCCTTCA

Dm TCGATTAGTT TATTATTTAA TTATAAGAAT TATTGGAGGT AGAATATTAA ATTGATTGAC ATTATTTGTA TGTATTGTAG GGGGTTTAAA AATTTATTTA

Ld TTACCTTTAA GAATTTTAGT TATTATAATA ATTGTTTTTT TTGTATAGAC AGGTAATGTT GTTAGTATTT ATTTATATGG GGTTATATTC TTTCTAACGT

Lb TGAGGGTTAT CATTAGTTAC GATTATAGCC GTTAATTTGT ATATTTTTGG AGGGATTGAT AGATATTTTT ATTTATATGG GGTTATGTTC TTTTTAATGT

Ls TTTATTTTTA TTTATATTTA TATATTTGTA TTATTTATAT TCACATTTGT GCCTATTGTT TCTAATTTAC TTATCTATGG GGATTTATTT TTATTAATAT

Bm GGCGTAATAA TTCTTATAGT TTCAATTTTC ATTATTTTGT AGACACATTT ACACATTTGT GGTTCTTTTT TTTCACATTC GGCTTTATCT TTTCATTTAT

Cb GTTTGTTGTT TTTTAGGGTT GAGAATTTTT TTATGTTTGT TTTCGTATAG ACTTAGGTCC TTTTATGTGT TTACCTAGGG GGTTTTGTTT GTTCTTGTAT

Cs ATGTGTTGTT GAAATGGGAA TAGTCTTTTT TTATGTTTTT TTTCATTTAG TCTTTGGTCC TTTAATGTGT TTACTTAGGG GGTTTTGTTT GTTCCTGTGT

Hm TTTTTTTTTT TATTAATAAT TTTATTTTTA ATTTGTCTAT AAATATTCGT TCGGTTTTGA GGATATTTGT ATGTTTTTAC GGCTTTATTT ATTCTATTGT

Ib TTAAGAATAG TTTTAATATT TTTGCAATTT TTTTATATAT AGTTTTATTT GGGTTTTTTT TCTTATTTTT TTATACTTGG GGTTTTATTT TTTCTTATCC

Pc ATTGTATTTA TTATCTCACT AAATTGGCTA TTTCCTGCAT ACGTTTACGT GGCTCTTGTC TGTTCTTTTG TTGGATTTGG GGTTATGTTC CTTCATGCTT

Ph ATTGTGTTTA TTATCTCACT AAATTGGCTA TTTCCTGCAT ACGTTTACGT GGCTCTTGTC TGTTCTTTTG TTGGATTTGG GGTTATGTTC CTTCATGCTT

Pp CTAACTATAT TCTTAAGATT AAATTGGTTA TTGAATGCTT ATGGGTATAT AAGTCTTGCC TGCTTTCTCT CTGGGTGTGG GGTTATGTGT ATTCGTATCT

Dm TTATTATTTG TATTTTGAAT TTTAATTTTA TTACTTTTAT CAACATTTGT TGTTCTTGTA TCTAATTTTT TTATTTTTGG GGATCTGTTT TTATTAGTAC

Ld AGGGTTGTGG AGATTATTTT ATTTTGTGCC AGCACTTGCG GTAAGACAGA CTAGGATTAG ATACCCTATT ATTGGCGGCC CAGAGGAATA TAACGTTCAG

Lb AGGTTTTTAT AGGTTGATTT TTTTTGTGCC AGCACTAGCG GTAAAACAGA CTAGGATTAG AAACCCTATT ATTGGCGGCT TAGAGATCCA TATGGGTCAG

Ls AGTTTTATAT CCATATTTAC ATATTGTGCC AGCATCTGCG GTTATACAAA CTAGGATTAG ATACCCTATT ATTGGCGGCA TAGAGGAATA TTTTGGTCAG

Bm ATTTATATAT CTTCTCTTAT TTTTTGTGCC AGCATAAGCG GTAATACAAA CTAGGATTAG ATACCCTACT A-TGGCGGCA CAGAGATGCA TAGCGGTCAG

Cb ATTATCTGTT TCATGTTTAT TTTTTGTGCC AGCACTAGCG GTTAGACAGA CCAGGATTAG ATACCCTGTT ATTGGCGGTT TAGAGAAACA TAACGGGCAA

Cs ATTTTCAGGT TTCTTTTTAT ATTTTGTGCC AGCTCTAGCG GTTATACGGA CCAGGATTAG ATACCCTGTT ACTGGCGGTT TAGAGAGACA TAACGGGCAA

Hm AGTATTATAA TGATCTATGT AAATTGTGCC AGCATTAGCG GT-----AAA CTAGGATTAG ATACCCTATT ATTGACAGTA TAGAGAAATA TAATGGTCAG

Ib ATTTTATTTT ATATATTTAT TTATAGTGCC AGCATTCGCG GTCATACAAA CTAGGATTAG ATACCCTATT ATTGGCGGCT TAGAGAAATA T----GTCAG

Pc ATTTTGATTT ACTCGTTAAC GCGTTGTGCC AGCACTAGCG GTCATACAGA CTAGGATTAG ATACCCTATT ATTGGCGGCT CAGAGTCGTA TAACG-ACAA

Ph ATTTTGATTT ACTCGTTAAC GCGTTGTGCC AGCACTAGCG GTCATACAGA CTAGGATTAG ATACCCTATT ATTGGCGGCT CAGAGTCGTA TAACG-ACAA

Pp GTTTGTCTGT CTATTTTTGT CTCTTGTGCC AGCACTAGCG GTCATACAGA CTAGGATTAG ATACCCTATT ATTGGCGGCT CGGAGTCGTA TAACG-ACAA

Dm TCTTATTTAT TTATTTATAC ATTTGGTGCC AGCAGTCGCG GTTATACAAA CTAGGATTAG ATACCCTATT ATTGGCGGTA TAGAGGAACC TATCGATCAG

Ld GTTGTGTACA GATCGCCCGT CACCCGGGTA AGTCGTAACA AAGTTAATTT ACCGGAAGGT GAAATAAAAC ATTACCTTTG GTATCAGGGG GAATCTGGTT

Lb GTAGTGTACA TACCGCCCGT CACTTGAATA AGTCGTAACA TGGTTAATCT ACTGGAAGGT GGAATATA-- -GTACCTTTG GTATCAGGGT TT-TCTGGT-

Ls GTAGTGTACA TATCGCCCGT CACTCAGATA AGTCGTAACA AAGTAGATTT ACTGGAAAGT AAATCTTGAA TGTACCTTTT GTATCAGGGT TTTTCTGGTT

Bm GTAGTGTACA AATCGCCCGT CACTCGGATA AGTCGTAACA AAGTTAGTCT ACCGGAAGGT GGACTAAGAG TGTACCTTTT GTATCAGGGG TGATCTGGTT

Cb GTAGTGTACA AATTGCCCGT CACTCGGAGA AGTCGTAACA TAGTTGGACT ACTGGAAAGT GGTCCAGGA- -GTACCTTTT GTATCAGGGT GGATCTGGTT

Cs GTAGTGTACA AATTGCCCGT CACTCGGAGA AGTCGTAACA TAGTTGGGCT ACTGGAAAGT GGTCCAAGA- -GTACCTTTT GTATCAGGGT GGATCTGGTT

Hm GT-ATGTACA AATCGCCCGT CAATCAGATA AGTCGAAACA CAGTAAATTT ACTGGAAAGT GAATTTATTA TGTACTTTTT GCATCACGGT TTATCTGGTT

Ib GTAGTGTACA AATTGCCCGT CAATCGGATA AGTCGAAACA AAGTTGATTT ACTGGAAAGT GAATCAAGTC TGTACCTTTT GTATCAGGGC TTATCTGGTT

Pc GTAGTGTACA TATCGCCCGT CATTCGGACA AGTCGTAACA AAGTTGATTT ACTGGAAAGT GAATCAGGAT AGTACCTTTT GCATCAGGGG TTACCTGGTT

Ph GTAGTGTACA TATCGCCCGT CATTCGGACA AGTCGTAACA AAGTTGATTT ACTGGAAAGT GAATCAGGAT AGTACCTTTT GCATCAGGGG TTA-CTGGTT

Pp GTAGTGTACA AATCGCCCGT CATTCGGACA AGTCGTAACA AAGTTGGTTT ACCGGAAGGT GAATCA---- -GTACCTTTA GCATCATGGG CTATCTGGTC

Dm ATAATGTACA CATCGCCCGT CGCTCAGATA AGTCGTAACA TAGTAGATGT ACTGGAAAGT GTATCTAGAA TGTACCTTGT GTATCAGTGT TTATCTAGTT

Ld -----GAACT CGGCAAACGT CTCCCAAAAA CAAAGTAAGG CCTGCCCATT G-TATAATTT TAACGGCAGC GGTACTCTGA CCGTATAAAG GTAGCATAAT

Lb ------AACT CGACAAA-GT TTACCAAAAA CAGGGTCGGC CCTGCTCACT G----ATTTT AAAGAGCCGC TTTA------ --GTGTTAAA GTAGCATAAT

Ls TTAAGGAATT CGGCAAATGT TTATCAAAAA CAAAATTTAA TCTGCTCACT G-TAAATATT AAAGAGCCGC AGTATTTTGA CTGTGCAAAG GTAGCATAAT

Bm TTAAGGAACT CGGCAAAAGT TTATTAAAAA CAAAGTAGGC CCTGCCCAAT G--ATTATGT CAATGGCTGC AGAATTTTAA CTGTACAAAG GTAGCATAAT

Cb TTTAGGAACT CGGCAAAAGT TTAATAAAAA CAAGGTATTG CCTGCTCAAT GGA---AAGT AAATAGCCGC AGTATTTTGA CTGTGCAAAG GTAGCATAAT

Cs TTTAGGAACT CGGCAAAAGT TTAGTAAAAA CAAGGTATGA CCTGCTCAAT GGAATGATGT AAATAGCCGC AGTACTCTGA CTGTGCAAAG GTAGCATAAT

Hm TTTAGGAACT AGGCAAAAGT TTATCAAAAA CAAGATAAGT TCTGTTCACT GCTATATGTT AAAGAACCGC AGGA----AA CTGTGCTAAG GTAGCAAAAT

Ib ATAATGAACT AGACAAAAGT TTATTAAAAA CAAGGTCTGA CCTGCCCCCT GTAAACAAGT TAATGGCTGC GGTATTTTGA CTGTACAAAG GTAGCATAAT

Pc TAAAGGAACT AGGCAAAAGT TTAATAAAAA CAAGGTAAAG TCTGCTCGG- -GTAATTGAT TAACAGCTGC AGTAACTTGA CTGTACAAAG GTAGCGTAAT

Ph TAAAGGAACT AGGCAAAAGT TTAATAAAAA CAAGGTAAAG TCTGCTCGG- -GTAATTGAT TAACAGCTGC AGTAACTTGA CTGTACAAAG GTAGCGTAAT

Pp TAAAGGAATT AGGCAACGGT CTAGTAAAAA CAAGGTATGA CCTGCTCAGT GGTAGATAGT AAATAGCTGC AGTAACCTAA CTGTAAAAAT GTAGCATAAT

Dm TTAAAGAATT CGGCAAATGT TTAACAAAAA CAAAGTCTAA CCTGCCCACT G--AAATTTT AAATGGCCGC AGTATTTTGA CTGTGCAAAG GTAGCTAAAT

Ld CACTTGGCTT TTTATTGAGG TCTGGTATGA ATGGCTTAAC GATTGGTTAA CTTTAGTTAA AATTCTTAAA TTGTATAAGG GGACGAGAAG ACCCTAAAAG

Lb CAATTGTCCT CTTATTAAGG TCTAGAATGA ACGGGAGAAT TATTGATAAA TTATATTTAT AATGCTTAAA TTAATTAAGG GGACGAGAAG ACCCTAAAAA

Ls AAATAGTTTT TTAATTGATA ACTGGAATGA AAGATTGGAT GAAAAATTGA CTGTCGTTAA AAAGCTAAAA TTTTTTTATA AGACGAGAAG ACCCTATAAA

Bm AATTTGTTCC TTAATTGGGA ACTGGAATGA AAGGGTTGAC AAGGCTTAAT CTGTCGTAAA AATGCTCCAA TTAAGAAGGG GGACGAGAAG ACCCTGAAAG

Cb AAATTGGCCT TTAATTGGGG TCTAGGATGA ATGGCTTGAC AAGGATTTAA CTGTCGTAAA AATGCAGACA TGTGAGAGAG GGACGAGAAG ACCCTAAAAG

Cs AAATTGGCCT TTAATTGAGG TCTGGAATGA ACGGTTCGAC AAGGATTTGA CTGTCGTAAA AATGCAAACA TAATAGAGAG GGACGAGAAG ACCCTAAAAG

Hm AAATTGTATA TTAATTGTAT TCCAGAATGA ATGAATTAAC AAGATTTTTA CTTTCGTTAA AATTCTTAAA TTAAGATGTA AGACGAGAAG ACCCTGAAAG

Ib AAATCGTCCT TTAATTAAGG TCTAGAATGA ATGGTTTGAT GAGGTTAAAT TTGTAGTAAA AAAACTGACA TTTTTCGAAG GGACGAGAAG ACCCTTAAAG

Pc CACTTGTCTT TTAATTGAAG ACTAGAATGA ACGGCTTAAC CAAGCTAATT CTGTCGTTAA AACGCTTAAA TTATTTAGAG GGACGAGAAG ACCCTGTAAG

Ph CACTTGTCTT TTAATTGAAG ACTAGAATGA ACGGCTTAAC CAAGCTAATT CTGTCGTTAA AACGCTTAAA TTATTTAGAG GGACGAGAAG ACCCTGTAAG

Pp CAATTGTCTC TTAATTGGAG TCTAGGATGA ACGGTCTAAC GTAACCCTGT CTGTCGTTAA AATGCTTAGA TGTAAAGATT GGACGAAAAG ACCCTATAA-

Dm CATTAGTCTT TTAATTGAAG GCTGGAATGA ATGGTTGGAC GAAATATTAA CTGTTGTCAA AAAGCTAAAA TTTATTTAAA AGACGAGAAG ACCCTATAAG

Ld TTACCTTAGG GATAACAGAG TGATATTAAG ATTGCGACCT CGATGTTGAA TTAGGAATTA GTCTGTTCGA CTATTCAACC CTACATGATT TGAGTTCAGA

Lb TTACCCTAGG GATAACAGCG CCATATTGTG ATTGCGACCT CGATGTTGAA TTAAAATTCA GTCTGTTCGA CTGGTT-ATT TTACATGATT TGAGTTCTGA

Ls TTACCTTAGG GATAACAGCG TAATTTTAAG TTTGCGACCT CGATGTTGAA TTAAAAATTA GTCTGTTCGA CTATTAAATT TTACATGATT TGAGTTCAGA

Bm GTACCTCAGG GATAACAGCA TAATAAAAGG CTTATGACCT CGATGTTGAA TTAAGATATG GTCTGTTCGA CCATTAAGTC TTACATGATT TGAGTTTAGA

Cb TTACCTTAGG GATAACAGGA CAATGCAACG TTTGTTACCT CGATGTTGAA TTAAGAGTTG GTCTGTTCGA CCATTAAAAC TTACATGATT TGAGTTTAGA

Cs TTACCTTAGG GATAACAGGA CAATGAGACG TTTGTTACCT CGATGTTGAA TTAAGAGTTG GTCTGTTCGA CCATTAAAAC TTACATGATT TGAGTTTAGA

Hm TTACCTCAGG GATAACAGCG TTATTTTTAG ATTGCGACCT CGATGTTGAA TTAAGAGTTA GTCTGTTCGA CTATTTAATC TTACATGATT TGAGTTAAGA

Ib TTACCTAAGG GATAACAGCA TAATA-AATG TTTATGACCT CGATGTTGAA TTAAAAGTTA GCCTGTTCGG CTATTAAATT TTACATGATT TGAGTTTAGA

Pc TTACCTCAGG GATAACAGCG CAATGAGAAG TTTGCGACCT CGATGTTGAA TTGAGAGTAG GACTGTTCGT CTTTTATAGC TCACATGATT TGAGTTTAGA

Ph TTACCTCAGG GATAACAGCG CAATGAGAAG TTTGCGACCT CGATGTTGAA TTGAGAGTAG GACTGTTCGT CTTTTATAGC TCACATGATT TGAGTTTAGA

Pp TTACCTTAGG GCTAACAGCG TGATAGGTGG TTTACGACCT CGATGTTGAA TTGGGAGTGA GACTGTTCGT TTCTTATTAC CCACATGATT TGAGTTTAGA

Dm TTACTTTAGG GATAACAGCG TAATTAAAAG ATTGCGACCT CGATGTTGGA TTAAGTTTAA GTCTGTTCGA CTTTTAATTC TTACATGATC TGAGTTCAAA

Ld CCGACGAAAG TCAGGTCAGT TTCTATCTTG GTACGAAAGG

Lb CCGACGTAAG TCAGGTTAGT TTCTATCTCA GTACGAAAGG

Ls CCGGCGTGAG CCAGGTCAGT TTCTATCTTA GTACGAAAGG

Bm CCGATGTAAA TCAGGTCAGA TTCTATCTCA GTACGAAAGG

Cb CCGACGTAAG TCAGGTCAGA TTCTATCTTT GTACGAAAGG

Cs CCGACGCAAG TCAGGTCAGA TTCTATCTTT GTACGAAAGG

Hm CCGACGTAAG TCAGGTCAGT TTCTATCTAA GTACGAAAGG

Ib CCGACGTGAG TCAGGTCAGA TTCTATCTTT GTACGAAAGG

Pc CCGACGTGAG TCAGGTCAGA TTCTATCTTT GTACGAAAGG

Ph CCGACGTGAG TCAGGTCAGA TTCTATCTTT GTACGAAAGG

Pp CCGACGTGAG TCAGGTCAGA TTCTATCCAA GTACGAAAGG

Dm CCGGTGTAAG CCAGGTTGGT TTCTATCTTA GTACGAAAGG

B

Ld ISTNHKEIGN LYFLFGVWSG VVGLSMSILM RFEGSSIFNW LITSHAFLMI FFMVMPMLIG GFANWLVPLM LGAPDMAFPR LNNLSFWLLP PSLLLLLSST

Lb LSTNHKDIGS LYFMFGVWSG LLGLSLSLLM RVESSSVFNS LITSHAFLMI FFFIMPMLIG GFSNWMIPLL ISSPDMAFPR LNNLSFWFLP PSLLLISFSM

Ls FSTNHKDIGT LYFLLGIWAG MVGTSMSILI RFEEDDIYNV IVTAHAFIMI FFMIMPIMIG GFGNWLIPLM LSAPDMAFPR MNNMSFWLLP PSLTLLLMSS

Bm FSTNHKDIGI LYMIFGVWSG LIGFGLSMII RIQFDGIFNV VVTIHAFLMI FFMVMPMMIG GFANWMVPIM LGAPDMAFPR MNNMSFWLLP PSLILLLMAT

Cb FSSNHKDIGM MYLIFGMWSG LLGYGMSVVI RTESDSIFNV FVTAHAFLMI FFMVMPIMIG GFANWLVPVM VGAVDMIFPR MNNMSFWLLP PSLVLLLMSS

Cs FSSNHKDIGV MYLIFGMWSG LLGYGMSVII RTESDSIFNV FVTAHAFLMI FFMVMPIMIG GFANWLVPVM IGAVDMIFPR MNNMSFWLLP PSLALLLISS

Hm YSSNHKNIGI LYMILGSWSG LLGFSLSMMI RLEFNPIYNV VVTSHAFLMI FFFIMPFMIG GFANWLVPIM NGSPDMSFPR MNNMSFWLLP PSLIFMLCSM

Ib YSTNHKDIGM LYLIFGIWSG LLGYSMSLII RMENDGIYNV IVTSHAFLMI FFMIMPIMIG GFANWLVPLM IGSPDMAFPR MNNISFWLLI PSLLFLLMSI

Pc FSTNHKDIGF LYLCSGVWFG LLGLSLSLMI RLESDSLYNV FVTSHAFVMI FFMVMPVMMG GFANWLVPSM LGSPDMAFPR MNNMSYWLLT PSGILLISSS

Ph FSTNHKDIGF LYLCSGVWFG LLGLSLSLMI RLESDSLYNV FVTSHAFVMI FFMVMPVMMG GFANWLVPSM LGSPDMAFPR MNNMSYWLLT PSGILLISSS

Pp FSTNHKDIGL LYLLSGIWFG LVGLSMSLIV RVEVNSTYNV FVTSHAFVMI FFMVMPVMMG GFANWLVPLF LGAPDMAFPR MNNMSYWLIM PSGVLLIASS

Dm FSTNHKDIGT LYFIFGAWAG MVGTSLSILI RAEGDDIYNV IVTAHAFIMI FFMVMPIMIG GFGNWLVPLM LGAPDMAFPR MNNMSFWLLP PALSLLLVSS

Ld FMDRGVGTGW TVYPPLSFSS VDLAIFSLHL AGASSILGAI NFITTFFNLS LYSWSVAITA ILLLLSLPVL AGAITMLLFD RNLNTSFFEP SGGGDPILYQ

Lb IVGPGAGTGW TAYPPLSAIE VDLVIFSLHL AGISSILGAI NFITTSINLP LFSWSVLITA FLLLLSLPVL AGAITMLLFD RNLSTSFFDP SGGGDPILFQ

Ls MTNVGAGTGW TVYPPLSAAV VDLAIFSLHL AGISSILGAV NFISTIINMP LFVWSVFLTA ILLLLSLPVL AGAITMLLTD RNLNTSFFDP AGGGDPILYQ

Bm CMESGVGSGW TLYPPLSSIL VGYLIFSLHL AGVSSIMGAI NFISTILNMP LFCWAVLITA ILLLLSLPVL AGAITMLLLD RSLNTSFFSP ELGGDPILYQ

Cb VIDNGVGTGW TVYPPLSSFV VDYAIFSLHL AGVSSIMGAI NFICTILNMP LFCWSVLITA FLLLLSLPVL AGAITMLLFD RNINTSFFDP SGGGDPVLYQ

Cs VIDSGVGTGW TVYPPLSSFT VDFAIFSLHL AGVSSIMGAI NFICTIMNMP LFCWSVMITA FLLLLSLPVL AGAITMLLFD RNINTSFFDP SGGGDPVLYQ

Hm MLDGGSGTGW TVYPPLSSLT VDMLIFSLHL AGISSIMGAI NFITTIFNMS LFNWSVLITA FLLLLSLPVL AGAITMLLFD RNFNSSFFDP IGGGDPILYQ

Ib FMGEGTGTGW TVYPPLSSQA VDISIFSLHL AGLSSILGAI NFICTIMNMP LFCWSILITA FLLLLSLPVL AGAITMLLLD RNINCSFFDP MGGGDPILYQ

Pc FVQGGVGTGW TVYPPLSSLE VDLAILSLHL AGVSSILGSV NFISTIFNMP LFCWSVLVTA FLLLLSLPVL AGAITMLLMD RNFNCSFFDP LGGGDPVLYQ

Ph FVQGGVGTGW TVYPPLSSLE VDLAILSLHL AGVSSILGSV NFISTIFNMP LFCWSVLVTA FLLLLSLPVL AGAITMLLMD RNFNCSFFDP LGGGDPVLYQ

Pp MIQGGTGTGW TIYPPLSPLE VDFTIFSLHL AGVSSILGSV NFISTILNMP LFCWSVLITA FLLLLSLPVL AGAITMLLLD RNFNCSFFDP LGGGDPVLYQ

Dm MVENGAGTGW TVYPPLSAGI VDLAIFSLHL AGISSILGAV NFITTVINMP LFVWSVVITA LLLLLSLPVL AGAITMLLTD RNLNTSFFDP AGGGDPILYQ

Ld HLFWFFGHPE VYILILPAFG IISHIVSSES YKDVFGVMGM IYAMSAIGVL GFVVWAHHMF TVGLDVDTRA YFTSATMIIA VPTGIKVFSW LATLYGSYIK

Lb HLFWFFGHPE VYILILPGFG LISHIISQES MKDVFGSLGM IYAMLSIGAL GFIVWAHHMF TVGMDVDSRA YFTSATMIIA IPTGVKVFSW LTTVYGSTVT

Ls HLFWFFGHPE VYILILPGFG IISHVISQES KKETFGVLGM IYAMMAIGLL GFVMWAHHMF TVGMDVDTRA YFTSATMIIA IPTGIKIFSW LATLHGSKMF

Bm HLFWFFGHPE VYILIIPGFG LMSHIINECS KPSAFGSLGM IYAMLTIGLL GFLVWAHHMF TVGMDIDSRA YFTSVTMVIA VPTGIKVFSW LGTIFGSKIN

Cb HLFWFFGHPE VYILILPGFG LISHMLSDNS KMEVFGSLGM IYAMVAIGVL GFIVWAHHMF TVGLDVDSRA YFTSATMVIA VPTGVKVFSW MATLFGSRVK

Cs HLFWFFGHPE VYILILPGFG LISHILSDNS KMEVFGSLGM IYAMLAIGVL GFIVWAHHMF TVGLDVDSRA YFTSATMVIA VPTGVKVFSW MATLFSSRVM

Hm HLFWFFGHPE VYILILPGFG LISHIIVQES KCETFGVLGM IYAMLSIGIL GFIVWAHHMF TIGMDVDTRA YFTSATMIIA IPTGIKIFSW LSTFFGSKMK

Ib HLFWFFGHPE VYILILPGFG LISHIICEES KKEVFGSLGM IYAMLSIGIL GFVVWAHHMF TVGMDVDSRA YFTGATMIIA VPTGIKVFSW MSTLFASNIN

Pc HLFWFFGHPE VYILILPGFG LISHMVVDCC KKEVFGSLGM IYAMSAIGAL GFVVWAHHMF TVGLDVDSRA YFTSATMTIA IPTGVKVFSW LGTLFGPKLK

Ph HLFWFFGHPE VYILILPGFG LISHMVVDCC KKEVFGSLGM IYAMSAIGAL GFVVWAHHMF TVGLDVDSRA YFTSATMTIA IPTGVKVFSW LGTLFGPKLK

Pp HLFWFFGHPE VYILILPGFG LISHMVVDLS KKEVFGSLGM IYAMVSIGVL GFVVWAHHMF TVGLDVDSRA YFTSATMTIA IPTGVKVFSW LGTLFGPKLH

Dm HLFWFFGHPE VYILILPGFG MISHIISQES KKETFGSLGM IYAMLAIGLL GFIVWAHHMF TVGMDVDTRA YFTSATMIIA VPTGIKIFSW LATLHGTQLS

Ld FSPSMIWAWG FIYLFTMGGL TGIMLSNSCI DIALHDTYYV VAHFHYVLSM GAVFAIFGGL IFWFPLFTGL LINPLKLKVH FFLTFIGVNL TFFPQHFLGM

Lb PSSSTLWSLG FIYLFTIGGL TGIILSNSSI DVILHDSYYV VAHFHYVLSM GAVFSIFSGL NFWLPLFLGG SVNELKNKVH FFLTFIGVNL TFFPQHFLGL

Ls FSPSSLWSLG FVFLFTIGGL TGVILANSSI DIALHDTYYV VAHSHYVLSM GAVFAIMAGF IQWFPLLTGL TLNNNWLKIQ FMIMFIGVNM TFFPQHFLGL

Bm WSLSSLWSVG FIFLFTLGGL TGVVLANSSI DTFMHDTYYV VAHFRYVLSM GAVFAMFASL FHWFPLFTGL TLNQKLMKIH FFVTFIGVNL TFFPQHFLGL

Cb WSPSELWGIG FIFLFTVGGL TGVVLANSSL DIILHDAYYV VAHFHYVLSM GAVFAVFGGF IHWFPVIFGV KMESVYLKVQ FFCTFVGVNL TFFPQHFLGL

Cs WSPSELWSVG FIFLFTVGGL TGVVLANSSL DIVLHDTYYV VAHFHYVLSM GAVFAVFGGL VHWFPVIFGV KMSSSFLKVQ FFSVFVGVNF TFFPQHFLGL

Hm FNSSELWSMG FVFLFTVGGL TGVVLANSSI DIVLHDTYYV VAHFHYVLSM GAVFAVFSAF THWFPLFFGV KMSNALMILH FWITFLGVNL TFFPQHFLGL

Ib WSVSSLWSLG FVFLFTIGGL TGVMLANSSI DIALHDTYYV VAHFHYVLSM GAMVAFMASL FHWFPLIFGV YLNSKFLKIH FFVTFISVNM IFFPQHFLGL

Pc SSISLLWSLG FIFLFTIGGL TGIVLSNSSV DVSLHDTYYV VAHFHYVLSM GAVFAIFGAW NHWFSLGTGL KLRKSFMNVH FWLSFVGVNL TFFPQHFLGL

Ph SSISLLWSLG FIFLFTIGGL TGIVLSNSSV DVSLHDTYYV VAHFHYVLSM GAVFAIFGAW NHWFSLGTGL KLRKSFMNVH FWLSFVGVNL TFFPQHFLGL

Pp LSVSLQWSLG FIFLFTVGGL TGIILSNSSV DVLLHDTYYV VAHFHYVLSM GAVFAIFGAW NHWFSSLSGC YLNPKVMSTH FWVSFIGVNL TFFPQHFLGL

Dm YSPAILWALG FVFLFTVGGL TGVVLANSSV DIILHDTYYV VAHFHYVLSM GAVFAIMAGF IHWYPLFTGL TLNNKWLKSH FIIMFIGVNL TFFPQHFLGL

Ld MSMPRRYSDY PDFFLFLNLL SSLGSWISLT AVIFFIFIIH ESFVKLNMVT EWLEGQPIKF QDSWSPYMSQ LANFHEYAMT YLIFIFSFIT VIMYMSSSIN

Lb SGLPRRYSDY PDHYTYLNLI SSIGSWISMI SIIWLITLIF DGVMKKNSVI EWIEGQPFNL LESSGPVMEQ MSEFHDHAMM ILFLIVSFLT IVFLTNKNLN

Ls MGMPRRYSDY PDIYTSWNMI SSLGSTISLI GIMFFIFIMW ESFISNRKPI EWLQKYPYNL QESASPLMEQ LIFFHDHSLL IITMITVMVS YIMFFNSNRF

Bm MGMPRRYADY PDLYTPWNSI SSIGSCISVV GLSMLIYAIY ESLISQRKAI EWLWGCPSKF MDPISFSGEI VQSVHDHVMI IITLIVMSIS YVFFCRSERL

Cb MGMPRRYSDY PDMFYSWNFI SSMGSQITLV GVSLFFFCLI EGFFSKRSVL EWMIGYPVEL QDSWGPLMSH ISGFHDHVMV VVLMILTVVV YINFFFPSRF

Cs MGMPRRYSDY PDMFYSWNVI SSFGSQISMI GVSLFVFCLI ESFLAKRKVL EWMIGFPVSL QDSFSPLMNH ISIFHDHVMV VVLMILTVVI YVNFFFPVRF

Hm SGMPRRYICY PDFYYSWNFY SSIGSMITSV SLLMFVFMIF YSFFENKKLL EWMLGTPIEL SDGCSLIMEN MVAFHDFTLM ILLFITTVVL MMLMITNNRF

Ib AGMPRRYMDY PDMFSSWNVI SSLGSTLSII SLFMMMFLIF ESLISKRLVV EWVNGFPTTF QDSNSPLMMH INHLHDHIMV VIIMIISIVM YVLVMNPNRF

Pc AGMPRRYSDY PDVYLSWNKI SSMGSLITTL GVVIFLLALM ESFSNPQKIV PRLMGMPSVF QDSNSPLMVF VCDTYDLVSI VCVGVISLVM YVAFFMKNYY

Ph AGMPRRYSDY PDVYLSWNKI SSMGSLITTL GVVIFLLALM ESFSNPQKIV PRLMGMPSVF QDSNSPLMVF VCDTYDLVSI VCVGVISLVM YVAFFMKNYY

Pp SGMPRRYSDY PDAFYGWNKI SSLGSMLTFV GVLLFLYASF DSVASANKVL VGLVGKPSDF QDGSSPVMAF VSNTYDLVCV VCVGVIALVA YVSLLNKNYY

Dm AGMPRRYSDY PDAYTTWNIV STIGSTISLL GILFFFFIIW ESLVSQRQVI EWYQNTPTNL QDSASPLMEQ LIFFHDHALL ILVMITVLVG YLMFFNNNRF

Ld TVDNEKLELL WTITILILLA VPSLNVLYFL EESFPQVSLK IIGHQWFWSY EFSDNKNFDS YMYRLLEVDK CVFLPKLVQI RGLVTSEDVL HSWAVPSLGL

Lb ILTSEVLEMF WSSLILLILA IPSIQVLFMM EEVIPLMTIK IMGNQWFWTY EYSDNVKFDS VIFRLLDVNK ALILPITTHV RLLLSSNDVI HSWTLPSYGL

Ls LLENQTIEMI WTIIVLIFIA LPSLRLLYLL DETKPSITLK TIGHQWYWSY EYSDNIEFDS FMFRLLEVNN RTILPFKTQI RILVTAADVL HSWAMPSLGV

Bm FQSSEILETV WTVFILVFAA IPSLHSLYIL EEEKPIISVK ILGNQWYWTY EFNTHINYNS YMLRNLEVDN NLVLPVGVET RAIITSSDVI HSWAIPPLGV

Cb MKSSEGLETL WTILVLASLA APSLMTLYLS DELSPVVTLK VIGHQWYWSY EYEDSSSFDS YMFRLLEVDK SVKVPLNSES RVFVTSSDVI HSWTVPCLGV

Cs FKSSESLETM WTILVLASLA APSLMTLYLS DELSPIITLK VIGHQWYWSY EYEDNYLFDS YMFRLLEVDK SVKVPVGGES RVFVTSSDVI HSWTVPCMGV

Hm LIYNEVLEFI WTVIILLIIA LPSLKILYLV DELLPEVTVK VIGNQWYWSY QYSDNIEFDS YMFKYLDVDN RTVLPVDTNI RMIITSSDVI HSWTIPSLGV

Ib FFGSEVLELI WTLAVLAILA IPSLHILYLM DELKPMISIK SIGHQWYWSY EYGDSIEFDS YMMRLLEVDN RTVIPVGMEI RMLITSTDVI HSWTIPTLGV

Pc FMGLESLEIV WVILSLAGLI LPSLHCLYLM DEVLPAMSLK VVGHQWFWSY EYGDNIEFDS YMFRLLEADL SVFIPYLTEV RAIVTSADVI HSWAIPMMGV

Ph FMGLESLEIV WVILSLAGLI LPSLHCLYLM DEVLPAMSLK VVGHQWFWSY EYGDNIEFDS YMFRLLEADL SVFIPYLTEV RAIVTSADVI HSWAIPMMGV

Pp FVGLESLETI WVILALASLV LPSLHCLYLM DEIYPSVTLK VIGHQWYWSY EYGDSIEFNS YMFRLLESDC SVYIPSSTEI RAIITSSDVI HSWAIPSLSV

Dm LLHGQLIEMI WTILILLFIA LPSLRLLYLL DEINPSVTLK SIGHQWYWSY EYSDNIEFDS YMFRLLDVDN RVVLPMNSQI RILVTAADVI HSWTVPALGV

Ld KMDACPGRIN FSLRSGKVYG QCSEICGVNH SFMPVVVNFV ELNKFHLWHD CHMVTQSPWP FFLSVAITNL ILNLFMSMFI IFLWSRDIVR ESTGQGCHTL

Lb KIDANPGRLN YSYRSGYFYG QCSEICGVDH SFMPIKVGFT SLDWFKNFSD FHLVDISPWP LMMSLTTANT ILSLYIKMFV FFLWSRDIMR ESTFQGMHPL

Ls KIDANPGRLN NINRPGLFYG QCSEICGAVH SFMPIVIESV HKNSFINWHP YHLVDVSPWP LTGAIGTMIL TSGVVKWFLT MFQWWRDVVR ESTFQGKHSI

Bm KMDAVPGRIN SISMSGLFYG QCSEICGSLH SFMPICVEAI PLKNWLKWHP FHMVQLSPWP LICSFSLFSM IILMYDFFLV LFEWWRDVCR ESTFQGWHSS

Cb KVDAIPGRLN YPSRVGLAYG QCSEICGSMH SFMPICLEVV PQEEFFRWHP FHVVDLSPWP LVMSLSVFSL ELNLYHFLLV SALWWRDVIR ESTFQGHHSE

Cs KVDAIPGRLN YPSRVGLVYG QCSEICGSMH SFMPICLEVI SQSDFFSFHP FHMVDLSPWP LMKSLSVFSL VVNLYHYMVI SVLWWRDVIR EGTFQGCHTK

Hm KLDANPGRLN LGNRLGLFFG QCSEICGILH SFMPICVEMV KPEWFLKWFL FHIVDESPWP LFLSFSVFLN MLSALVYLLI FYMWMRDMIS ESTMQGMHTL

Ib KMDGVPGRLN SSNICGLMYG QCSEICGSFH SFMPICLEVL SESRFMSWHP FHIVSISPWP ILCSFSIMSF VINSLYYMLV IFCWWRDVIR ESTFQGFHMK

Pc KVDAIPGRLN YSFKIGTSYG QCSEICGAYH SFMPIKVTTL PKEDFMKWHP FHLVDVSPWP IFLSFSLLFS ASMTLCWILI VSFWWRDVTR EATFQGKHTM

Ph KVDAIPGRLN YSFKIGTSYG QCSEICGAYH SFMPIKVTTL PKEDFMKWHP FHLVDVSPWP IFLSFSLLFS ASMTLCWILI VSFWWRDVTR EATFQGKHTM

Pp KMDAVPGRLN YSHKLGSLYG QCSEMCGAYH SFMPISVKTV PKAVFIAWHP FHLVDFSPWP LYLSLSSLCF AVAVLCILLV LSLWWRDVIR ESTFQGKHTI

Dm KVDGTPGRLN FINRPGLFYG QCSEICGANH SFMPIVIESV PVNYFIKWHP FHLVDYSPWP LTGAIGAMTT VSGMVKWFLT VYQWWRDVSR EGTYQGLHTY

Ld KLIKFGMALF ILSEVMFFFS FFWAFFDYAL NPSLEIWPPK GVVPLNPMHV PLLNSLILVS SGLTITSAHM FLNKSKAMMW TSLTVILGIY FTILQILEYF

Lb KSLKYGMILF ITSEVMFFLS FFWTFLHSAL SPTNEIWPSW GVEPINPFGI PLLNTLVLVS SGVSITYSHH SMNFNFTILW VVITVLLGGY FTILQLMEYF

Ls SGMRWGMILF ITSEVFFFIS FFWAFFHSSL SPSIEIWPPK GIQPFNPFQI PFLNTVILIS SGITITWAHH SLNNSQTIQS LVITIILGLY FTILQGIEYF

Bm NGLKIGFIMF ICSEVLFFFS FFFGYFFLSL NPDVVFWPPK GLMVVDFLSA PTLNSILLLS SGVSITWAHH SINLSEAKMG LVYTVFLGIM FSMIQLIEYF

Cb EGLVLGVLLF ICSEVMFFFS FFFGFLFSAL CPDIEIWPPL GIEPLNFMMV PLMNTLILLS SGVSITWSHH SIDWKNSLFG MVITVFLGFV FSFLQYEEYF

Cs EGLVSGVLLF ICSEIMFFFS FFFGFLFSSL CPDVEIWPPV GNEPLSFMMV PFLNTLILLS SGVSITWSHH ALNLFSSLYG MMVTVFFWLV FSFFQFDEYF

Hm KGIKMGMVLF ITSEVMFFFS FFWSLGYYMV SHEYILWPLL GIMSLNPSTV PLLGTMILLS SGVSVTWCHN ELNLSSMKNS LLITVILGMV FAALQMWEYF

Ib KGLYMGVSMF IISEVMFFFS FFFGYFFSSL VPDVEIWPPV GVQSLSFMDV PLLNTMILLS SGISITWSHH SLNFTNCLLG MIFTVILGLI FTFFQFMEYF

Pc EGLRLGMLMF IASEVMFFFS FFYALFFLSL SPDVSLYPPV GVSPVGVLGV PLLNSILLLS SGVSITWAHY ELNISSSLIG LLITLILGLV FLMFQAVEYF

Ph EGLRLGMLMF IASEVMFFFS FFYALFFLSL SPDVSLYPPV GVSPVGVLGV PLLNSILLLS SGVSITWAHY ELNISSSLIG LLITLILGLV FLTFQAVEYF

Pp EGIRLGMILF IMSEVMFFFS FFYGLFFLAL NPDVTLFPPV GLASMGLLGV PLLNSFLLLS SGVSVTWSHY EIDVPKSLLA LAMTLALGTV FLAFQYLEYF

Dm AGLRWGMILF ILSEVLFFVS FFWAFFHSSL SPAIELWPPM GIISFNPFQI PLLNTAILLA SGVTVTWAHH SLNHSQTTQG LFFTVLLGIY FTILQAYEYF

Ld SFTDSAYGSI FFLTTGFHGF HVIIGTILIL VSLVRMYMNH FSSKRHLNFE MACWYWHFVD FVWLFLYISI YWFSIFDLLF IINSEAFMFI FFLNLYGLLS

Lb SIMDSVYGSI FFISTGFHGI HVLVGTLMIL YSLIRLFSFQ FSSAHHLMFE FSCWYWHFVD LIWLFLFLSI YWFSIFDLIL LIIKEMFILI LAFNISGIFP

Ls SIADAIYGSS FFMATGFHGI HVMIGTTFIL MMLIRQMNNH FSNYHHFGFE AAAWYWHFVD IVWLFLYISI YWFSSFDLLF IMHNEFFSFV LINNFLGLFP

Bm TIADSPFGSM FFLATGFHGI HVLVGTIFII ISFVRLLNNQ FSKNHHVGFE MSCWYWHFVD VVWLFLFVRV YWLSVFDSLI GFSMGWFITI FCINEMGMVP

Cb TMADSVYGSF FFLMTGFHGI HVIVGVLFIM VSLFRTLVGH FSKSHHFGFE AAAWYWHFVD VVWLFLFVTV YWSFLYSCAG FFFDLLVFFF FFSNLLGLVP

Cs TMADSIYGSF FFLMTGFHGI HVMVGVLFIS VSLFRLMMGH YSSFHHFGFE ASAWYWHFVD VVWLFLYVMV YWSFLYKFFF FFFWLLVFFF FFSNLLGLIP

Hm TMSDGVYGSL FYMMTGFHGF HVIVGTIFLF IIFLRLKNYH FSSHHHLGFQ AAAWYWHFVD VVWIFLYIML YWLSIFDMSL CLMKSMFFLI LVFNILGIFC

Ib SMADSVYGSL FYISTGFHGI HVIVGTLFII VSFIRMMKYH FSIHHHLGFE FSIWYWHFVD VVWLFLFLSV YYFSIFDSLS WMISSSFTMI LMCNQLSMVP

Pc TMADSSFGSV FFLMTGFHGA HVCVGVVFIT ISTIRLYLNH YNNNHHLGLE LAAWYWHFVD VVWLFLYLTL YWMSSFDILF PFLLMKFMMI LTLNTISLMP

Ph TMADSSFGSV FFLMTGFHGA HVCVGVVFIT ISTIRLYLNH YNNNHHLGLE LAAWYWHFVD VVWLFLYLTL YWMSSFDILF PFLLMKFMMI LTLNTISLMP

Pp SISDSAYGSI FYLMTGFHGF HVMVGVVFIL VMTVRLAKGH LNSSHHTGFE LSAWYWHFVD VVWLLLFVSL YWMSMFDATL VCLHELFYCV LSMNVISLIP

Dm TIADSIYGST FFMATGFHGI HVLIGTTFLL VCLLRHLNNH FSKNHHFGFE AAAWYWHFVD VVWLFLYITI YWFSVFDFLG LLHKEFFSLI LFNNFMGLFP

Ld YVMAVFSHMS ISFSLSMLFW TSIMLMSHLV PLGCPNGLMF FMVIISILIR PITLGVRLSA NIMAGHVILG LTVQFLILEF AVALVQPYVF FTLLCLYMKW

Lb FTFTLTSHMS MTFSLAFPMW LSLMIFAHLV PLGCPTVLMP FMVLISLIIR PLTLAVRLAA NMIAGHMILS LLAESLLLEL AVAMIQPYVF FILLTLYSQW

Ls YIFTSTSQLV LTLTLSLPLW LSFMIFAHLV PNGTPGILMP FMVCISNIIR PGTLAVRLTA NMIAGHLILT LIIQILMLEM AVAFIQSYVI AILITLYSSW

Bm YLFTPTSHFS LNLCLALPLW LTGIIFSHLV PEGSPMGLAP LLVIISLLIR PISLSIRLMS NIMAGHMILS LMVVFMSFEL CVGIVQAYIF SCLLVMYWSW

Cb YVFTLSSHLC VNLSVSLVLW LGGVVLSHMV PLGSPVFLLP LLVLISTLIR PLTLAIRLMA NVMAGHLIMS LFIELLFFEM CVAVVQAYVF SSLMVMYLAW

Cs YMFTLSSHLC VNMSVSLVMW MGGVILSHMV PLGSPVFLLP LLVVISMVIR PLTLSIRLMA NVMAGHLIIS LFFEVLFFEM CVSIVQAYVF SSLMVMYLAW

Hm FTFSVTSHLV INLSLGFSIW VGTLLLAHLT PMGCPMVLVP FMVVISMMIR PITLSLRLMA NMLAGHMILS LLLLLYLFEI GVAIIQAYVF SILLSLYWEW

Ib FVFGPTSHLS FNSAVALSSW LAGIIVSHFV PLGSPMFLTP FLFIVSCLIR PVALSVRLMS NMMAGHIIIV LIESFFLFEL CISIVQAYVF SSLLALYYKW

Pc LVLPCTSHLS VNLGLCLPLW MSGVVLAHLL PYGSPIMLSP FLVVISVSIR PVSLSVRLLA NITGGHLIMN LAAYVLAAEL FVSFIQSYVL SKLVSIYWEL

Ph LVLPCTSHLS VNLGLCLPLW MSGVVLAHLL PYGSPTMLSP FLVVISVSIR PVSLSVRLLA NITGGHLIMN LAAYVLAAEL FVSFIQSYVL SKLVSIYWEL

Pp FTLPFTSHIS VNLGMCLTLW LSGLLLAHYL PLGSPMVLGP FLVLISVLIR PISLSVRLMA NILGGHIIMS LLTYSVCVEM FVATVQAYVL SKLLSIYWEW

Dm YIFTSTSHLT LTLSLALPLW LCFMLFAHLV PQGTPAILMP FMVCISNIIR PGTLAVRLTA NMIAGHLLLT LMAQILVLES AVAMIQSYVF AVLSTLYSSW

Ld NFGSLLGISL TVQIFTGFFL SFHYSAEFDK ILNMCNEISY GWMLRFLHST GASMFFILCY LHIGKALVYK SYWASGLMIL LLIMGTAFLG YVLPWGQMSL

Lb NLGSLLGLCL SIQILTGVFL TMFFKADFTS VVSIMNNINN GWIIRFIHST GASMFFIICY AHVGKALFFS SFWVSGLVLI LLLMMEAFLG YVLPWGQMSF

Ls NFGSLLGLCL IIQISSGLFL AMHYSAHFSS MIHICRDVNN GWILRTIHAN GASFFFICLY LHVGRGIYYS SYWFIGILIL LLTMATAFVG YVLPWGQMSF

Bm NFGSLLGINL LLQIVSGIFL AMHYEDTFES VVSMMNDMNS GWLIRFIHAN GASLFFVLLY FHIGRGLYYG SYWIVGVMIM FILMGTAFVG YVLPWGQMSF

Cb NFGSLLGVCL MIQLVSGIFL SFHYSPTFSS VVMIVDDVPF GWMFRSIHAN GASFFFFWVY LHIGRGLYLS KYWMKGVLIF FFLMGTAFMG YVPPWGQIFL

Cs NFGSLLGLCL FIQLVSGIFL SFHYSPSFLS VVMIEDDVPF GWMFRSVHAN GASFFFFCIY LHIGRGLYFG SYWISGVLIL FLLMGTAFIG YVLPWGQMSL

Hm NFGSLLGLCL FIQIGSGLFL SLHYNSNFSS VIYMMNDVNH GWILRVIHAN GVTMMFIFMY IHIARGLYYK SYWLVGILIL LLTMGTAFLG YVLPWGQMSF

Ib NFGSLLGMCL MVQIFSGLFL SMHYNTSFNS VLSTCNDVNL GWLIRYIHAN GASMFFMLVY CHIGRGLYFG SFWFSGVIIL LLLMGTSFLG YVLPWGQMSF

Pc KFWVLLGLFL SIQILSGLFL ASHYEASFWS VILIDFDVNS GWLIRSFHAN GASFFFILVY VHIWRGLWFG CFWFSGISIL LLMMAAAFMG YVLPWGQMSF

Ph KFWVLLGLFL SIQILSGLFL ASHYEASFWS VILIDFDVNS GWLIRSFHAN GASFFFILVY VHIWRGLWFG CFWFSGISIL LLMMAAAFMG YVLPWGQMSF

Pp NFGSLLGCFL VVQVFTGFLL STHYEASFES VLLIEFDALE GWLVRSLHAN GASWFFILAY FHIWRSLWFG CFWVSGILIL LLMMAISFLG YVLPWGQMSF

Dm NFGSLLGLCL IIQILTGLFL AMHYTADFYS VNHICRDVNY GWLLRTLHAN GASFFFICIY LHVGRGIYYG SYWLIGVIIL FLVMGTAFMG YVLPWGQMSF

Ld WGATVITNLI SVIPYFGKDM VEWLWGGFNV GTFTLNRFYS LHFILPFLVA LMVVIHIVFL HSSGSSNPMG IDKISFFNYF VIKDMVTVVI MMAVLFFFSL

Lb WGATVITNLI SVIPYFGPLA VQWLWGGFNV GDPTLTRFLS FHFIIPFIMI AMSGVHLILL HETGSSNPLG MDKVSFSKFF IIKDLVTLAL VLLGLILLST

Ls WGATVITNLL SAIPYLGQML VQWIWGGFAV DNATLIRFFT FHFILPFIIL AMSIIHLLFL HQTGSNNPLG IDKIPFHPFF SIKDLFGYMI MLLILISLNF

Bm WGATVITNLV SAVPFIGTDM VIWLWGGFSV DNPTLVRFFS IHFVLPFVIL AMVILHLLFL HSTGSSNPLG SDKVYFHPLF SIKDILGLII VTFFFLSTVF

Cb GGATVITSLL SAIPYMGGFL VKWVWGGFFV KGPTLHRFFS LHYLLPLILS VFVFFQVFFF QSKSGVNPMG SKKGFFVPYF FFVDLGGIFF FFFFFFMFVF

Cs WGATVITSLL SAIPYLGSFL VEWVWGGFSV SGPTLHRFFS LHYLLPIVLF FFAMVHIFFL HEKGSSNPMG SDKVYFVPYF LSVDLVGVFF FFFFFFLFIF

Hm WGAMVITNLI STIPYLGVTL VEWVWGGFSV SEPTLTRFFS FHFILPFVIL GASALHIIFL HKYLSSNPLG TDMISFHPFF TVKDILGVVL FLFSLLFLSL

Ib WGATVITNLV STIPYVGDQL VYWLWGGFSV SEPTLNRFFS IHFILPFVLM MVVLVHIFSL HKSGSSNPLG CLKISFHPYF WNKDVLGFVV VLIIFTVTLI

Pc WGATVITNLL SAIPIVGSDL VIWVWGGFSV SHPTLERLFT LHFLLPFVLL GFVMAHIILL HQHGSSNPLG SDKVYFYPYF YLKDILGGFV CLFLFVLICI

Ph WGATVITNLL SAIPIVGSDL VIWVWGGFSV SHPTLERLFT LHFLLPFVLL GFVMAHIILL HQHGSSNPLG SDKVYFYPYF YLKDILGGFV CLFLFVLICI

Pp WGATVITNLF SALPFVGSEL VTWIWGGFSV GSPTLERFFS SHFMLSMVLL CFVIFHITFL HENGSSNPLG SDKVYFYPYF MLKDLLGGLV AMTIYFSLGL

Dm WVATVITNLL YAIPYLGMDL VQWLWGGFAV DNATLTRFFT FHFILPFIVL AMTMIHLLFL HQTGSNNPIG IDKIPFHPYF TFKDIVGFIV MIFILISLVL

Ld YKPYLLMDHE NFITANPMLT PPHIQPEWYF LFAYAILRSI PNKMGGVVML LLSLFIILLI IIFWFHVGTF IILTWLGSMP VDSPFSKFYT VIYFCFYLQV

Lb MSPFMFMDPE NFLKANPMVT PIHIQPEWYF LFAYAILRSV PNKLGGVLML ALSIIIILIK WLLYFHFGSF SILTWLGMQP VEDPFGKVYS VLYFIFYFNL

Ls SMPYILGDPD NFTPANPLST PVHIQPEWYF LFAYAILRSI PNKLGGVLAL LFSILILYIK ILFWSFTTIF ILLTWAGAKP VEDPFSQILT VLYFSFFINF

Bm LKPESLMDPD NFTPANPMST PQHIQPEWYF LFAYTILRSI SSKFGGVMAL VFSILILMFK FFFWIQVSNF ILLTWLGSMP VEQPYGQMVS ISYFSVFLES

Cb VFHDVLMDPD NFIPANPMST PPHIQPEWYF LFAYTILRSV PSKLGGVVAL VFSILFLVFK FLVVVLFVVF FLLTWIGSMP VEYPYGKVLS VFYFFVLMEV

Cs KFHDLLMDPD NFVPANPMST PPHIQPEWYF LFAYSILRSI PSKFGGVMAL VFSILFLVFK FFVVVLFVIF FLLTWIGSMP VEFPYGKVLS MIYFFVLIEV

Hm TEPYKFMDPD NFILANSMVT PVHIQPEWYF LFAYSILRAV PNKLGGVIGL LMSILVLALK SFCWVQFTIF MLLTWTGSLP VESPFGQCLS VMYFLNMFQH

Ib FLPDVFMDPD NFSVANPMST PAHIQPEWYF LFAYAILRSI PTKLGGVVAL VFSIVILFIK MIVLMQVSNF LLLTWLGAMP VEFPFSKIFS SMYFIFMIQF

Pc YSPDFFMDPD NFVESNPMIT PPHIQPEWYF LFAYAILRSV PNKLGGVVAL LLSILSLSLM ILTYSFTSVF VMLSWLGSLP AEYPFSQVVS VIYFIQVIQS

Ph YSPDFFMDPD NFVESNPMIT PPHIQPEWYF LFAYAILRSV PNKLGGVVAL LLSILSLSFM ILTYSFTSVF VMLSWLGSLP AEYPFSQVVS VIYFIQVIQS

Pp YSPDLFMDPD NFMEANPLVT PPHIQPEWYF LFAYAILRAV PSKLGGVVAL VMSIVSLVVK VLVYSLVVSV LILSWLGAMP AEVPFSQVAS VIYFTLILQG

Dm ISPNLLGDPD NFIPATPLVT PAHIQPEWYF LFAYAILRSI PNKLGGVIAL VLSIAILMIQ VMFWSMLVTV ILLTWIGARP VEEPYGQILT VVYFLYYLSL

Ld VIFLLLIVAL FTLFERKVLG FAQNRKGPDK VGNKGVLQPF ADAAKLFSYL YSAFFFFFIP LILCMSKNFN WGLWYKSFLF ILMLFSFNVY GTILSGWSSN

Lb ILHILLSVAL FTLFERKILG LIQLRKGPCK VGPLGLLQPF SDALKLFSYY FTPLYFLILS LVFFLNKPFL STSFTLSILV LLFLYTTSVY TTLVTGWSSN

Ls INFILVGVAF LTLLERKVLG YIQIRKGPNK LGFIGLLQPF SDAIKLFTYY FSPVFLFFLS LISWMIMPYF WVLLSLGLLF FMVCLSLGVY GIMISGWSSF

Bm IQLILLSVAF FSLFERKVLA IIQNRVGPDK VGIVGILQPF SDAMKLISYF FSPAIMFVIS FIVWITFPSN WEIFDKSMLF VIACMGSSVY GLVMTGWFSS

Cb VEVLLLSVAF FSLYERKLMG LVQGRKGPNK VGVGGVLQPF ADAMKLISYA VSPMISFFIS LVFWIMYPVI WNFLNLSIVF LLVLFSVSVY GFILSGWFSS

Cs FEILMMSVAF FSLYERKLMG LIQNRKGPNK VGLGGVMQPF ADAMKLIGYI VSPMVSFFIS LVFWILYPVV WNFVNFGIFF LLVMFSVSVY GFIMTGWFSS

Hm FILVLLTVAF FTLLERKILG YIHFRKGPNK VLLKGVLQPI VDAMKLITYY ISPMFSFIMS MIIWMILPLQ FIIWVNSFLI LFMLLGMGVY SMFLSGWSSN

Ib LLIILLSVAF FSLFERKMLS VIQFRKGPNK VGLMGFFQPF SDAIKLLFYY FAPMVFFILS ILIWISIPSK WNLFSSSFIF VMFLYGIPIY SMIFMSWISN

Pc VFIVLICVAY FSLFERKLLS LEQIRLGPNK VGPIGILQPL SDAPKLLSLF IMPFITFMLS VSWWYPLYFP KTLSNNSLLI LIFISSVSVY ALIFTGSLPK

Ph VFIVLICVAY FSLFERKLLS LEQIRLGPNK VGPIGILQPL SDAPKLLSLF IMPFITFMLS VSWWYPLYFP KTLSNNSLLI LIFISSVSVY ALIFTGSLPK

Pp FMTVMVCVAY FSLFERKMLG LTQLRLGPNK VGPTGVLQPL SDAPKLLTYA FVTFLSFLLS LTYWFSIPIK FTPSDNMGIW IIIGLSLVVY GPVSCGWFSH

Dm IGSLLVSVAF LTLLERKVLG YIQIRKGPNK VGLMGIPQPF CDAIKLFTYY ISPIFSLFLS LFVWMCMPFF VKLFNLGGLF FLCCTSLGVY TVMVAGWSSN

Ld SKFGSIRSVA QTISYEMVAL SSYTMYTKMY LFFPMWMSVI IEVNRTPFDL AECESELVSG FNVEYGGIEF SLIFLGENLM VVIFIFYFIW IRASFPRFRF

Lb SKYGSMRSIA QSLSYEITAF IMSSTLMNFL QLFYCPLSMI IESNRTPFDL SECESELVSG FNVEFGGAEF SLIFLGENLM LVVIIFIKVS IRGAYPRYRL

Ls SLYGSLRSVA QTISYEVSLM LIGNYSYFYY LYMFGFPLCL AEVNRTPFDF AEAESELVSG FNIEYGMGGF ALIFLAEYSS IFFVSFFFIW VRGSLPRFRY

Bm SKYGSVRAIG MSISYEIIMF LLGTMSISFW LFFPLWFVFI AESGRAPFDL SEGESELVSG YSVEYGGISY TLIFLSENSS IIGLIFLVVW IRGTVPRMRF

Cb SKYGCARALA QSISYEVGCL FFSCISLEVF FFFPFSFLFV AETNRPPFDL AEGESELKKG FCVKKGEMSN PLIFLGENLS VLLWGGFLFL FVLLYLDFGM

Cs SKYGCARALA QSISYEIGCL FFSSISLKVF FFFGFFSLLV AKTNRSPFDL PKGEKKLVSG FCVEYGGMSY TFIFLGENMS VLMLLSFFVI VRSALPRFRF

Hm SKYGSLRAVS QSISYEILMM TTKGMSLKFF FLFPFFIAYL AELNRSPFDL SEGESELVAG YTVEYGGIMY TMIFLSENIM IMMMIYLVCL IRGILPRIRY

Ib SKYGSIRSVA QSISYEIIMM MVYSSSYFYW LLYPCFPIFF AESNRSPFDL TEGESELVSG IFVELGGVWY ILIFLGENLY LLIITMLMVW IRGTVPRIRY

Pc SKYGSLRAIT LSISFELVAV VFNSFSATNP NIISMIVVGI AECGRTPFDL PESESELVSG FNVEYGGSRY VLLYLSESLL LTTWISISIV MRASAPRIRY

Ph SKYGSLRAIT LSISFELVAV VFNSFSATNP NIISMIVVGI AECGRTPFDL PESESELVSG FNVEYGGSRY VLLYLSESLL LTTWISISIV MRASNPRIRY

Pp SKFGSTRAIA QCISFELVFS FSSCTSCMAP NLLTAPLLAF AESGRSPFDL PEGESELVSG FNVEYGGVRY ILIYLSESVT LMIWVVMSIL LRSSLPRMRF

Dm SNYGGLRAVA QTISYEVSIF LIGSYNFFYW FLIILFPMAL AETNRNPFDF AEGESELVSG FNVEYSSGGL ALIFMAEYAS ILFISFVFIW VRGTLPRFRY

Ld DKMMYLCWLI LVPLSVSMPW LIFMMLGIMV GISSEAWLGA WFGMEFFLPL YYLMQALGSV FFIYSFLYII MKIGVSPFFF WIYPSFKWIP NKPLMMFMTL

Lb DSMMELCWLI YLPLTIVLLS SLFLLLNIIL LSSSLDWIIL WVGLEIFLSI YFIVQFFGSS VFLIGWLLFM IKLMLLPFLN WVLESLKWIS SKLQLIFLSI

Ls DKLMNLCWKS FLSLSLFILS YHFCFLGTII SISANSIFMS WMGLESFIPM YFLIQAMGSS LFLFSSLIMM LKLGSSPFHN WFINIIEGMS WMTCFLILTW

Bm DQLMMMCWVK ILPIMLFIFG LVMIYISSYG LFMSSSFLMF WVLMEFFISI YFSIQAVGSA WLVLGIYVVL MKLGMIPFHG WAMELAEGLS GKNLNLFLTL

Cb MNWRLYFGLK YCLYNCSFQF FFLFFFSVVV TLSSSSWLVC WMMMEFFIPI YFFVQSIGSS VFLFSIITCM FKLGVFPFMG WMVQISENLS WSKFFLLSSI

Cs DKVMMMFWIE VLPLQMFFCV LFLVLLSVLI TFSSSSWLVC WMMMEFFVPM YFVVQSIGSS IFFFSVLTCM IKLGVFPFSG WMIHISENLS WSKFFLLSSI

Hm DHLMMFCWKI MLPLMVIFVN LTYFVLTLIL IYSMSNFWIF WLSMELFSSL YILFQSIPSS LFLMLNLVFT MKIGIYPFHW WFFNLVENLN WMSFFFFNSI

Ib DKMMDLCWIS MMPICMSFIS IFFLVLTLFI IFSCTNWVIM WVGMEFFISL YFLIQSISSA LMFLSFLMTM IKLGMPPFHS WMFKICENME WDMFFLFNTI

Pc DKCMMFGWEF SIPLILMFMS IYVLLFSCLF SVSSMSWWPA WLGLEWFFPL MFSLSCLSSM GLLLSLMSFS MKMGLPPFHN WYLDVGDSLD SWSFMVLMTA

Ph DKCMMFGWEF SIPLILMFMS IYVLLFSCLF SVSSMSWWPA WLGLEWFFPL MFSLSCLSSM GLLLSLMSFS MKMGLPPFHN WYLDVGDSLD SWSFMVLMTA

Pp DHCMALGWVT LLSSAISYAL AIILIVSSFL SLSSLTWWCA WLSMEWLYPI FFLFQSVSSV LLFLCFISLL LKMGFPPFHS WYLELCDSVS STGFILINTV

Dm DKLMYLAWKC FLSFSLNYLL FFIGIIGTLI TVTSNSWLGA WMGLESFIPL YFLTQVLAST VLLFSSIIML LKSGAAPFHF WFPNMMEGLT WMNALMLMTW

Ld QKILPLYLLI FFFGVIDIVL FSSIYQMSWV ILSLIMSKYM FKFYFMYYIM FSMVAMIMWF IFFLISGFPP SIIFLMKIHI IMNIMILFNL FMINYLKFSD

Lb QKIGPFFLKT FLIMGMDLLV ISSNGQSVLI IILLLFSKAI GLIYLFVYMT FFLIFYETIF MFIFISGFPP SLIFFAKLNF LLMSGIIIFT MFFVYMKVFI

Ls QKIAPLCLMM FIIGGRKILA FSSINHLGWM ISSMILSKYI LILYFLIYSF TLIMIFFLFL SLLSLGGLPP LLGFFPKWLI IEKMLNFILI TIFYYLQIIM

Bm TKIGPMLAVM LVVMARQFLV LSSIVNLCWI MLSSAMSMYL SVMMMLFYFI AVLTIFVFIV AMISLWGIPP FMMFMVKYST MEMLPCLIML LLITYLSMIN

Cb QEVIPLLMFF FVCFCHWMMM VSSLFNVGWM VVAALLSKEG LIFFFFVYFS VFFWVLVFLF FVFMMMGIPP LGNFLGKVEV LTSWMSVYFV FMFLYLKWVV

Cs QEVIPLLMFF FLVFCHWIMM VSSLFNVGWM VVASLLSKGS LVFFMTIYFS VFFWILVFLF VVFMMMGVPP LGNFLGKVEI VSSWLSFFFL FMFLYLKWEF

Hm VKLVPTLVLI FFLFSRMILS FSSMIHFSWI ILCSILSVSY FQWYILIYFL MLINVLYILF LLFSFMGIPP LLGFLMKMFV VYGLLSLLLM PMIFYWKILL

Ib QKISPMITVM YFVLCRRFIL FSSILNTNWM LMCMMASKFV FLEYLVFYTF CLCKLLMTML CILTICGLPP FLGFFIKVEL INYLLVFMNM MMYMYLHMLL

Pc FKVPSLVFLM LYAAGRRMLI YSSILNVVWS LVSLFYSLSL FLTFYCIYFN CVFWFLILGL SLANLAGMPP LSMFFMKMKI ISSVLTTASL MLWIYMSPLS

Ph FKVPSLVFLM LYAAGRRMLI YSSILNVVWS LVSLFYSLSL FLTFYCIYFN CVFWLLILGL SLANLAGMPP LSMFFMKMKI ISSVLTTASL MLWIYMSPLS

Pp VKLPPLWLLM VLCSGRRMMI YSSFVNYVWG LASMGLSVSA FVTFFLNYFI LTFWFMVFMA SWLSLSGLPP FGLFFAKALV AYELTLCLAF MMWIYLSPFM

Dm QKIAPLMLLI SVIGGRKLMA FSSINHLGWM LSSLMISESI WLILFFFYSF VLTFMFTLFM NFLSLGGLPP FLGFLPKWLV IQQFMLTIMM TLFFYLRIIF

Ld WVVFYVLMFV FFESGITYSL SFFMITLIFL LFDIEIILLM PFFIYLLFLL ILEWNMQSLE WKNIFFLLIS YIFFDLFVDK YSSMFSLIVN LITLCIFLYS

Lb FVLTLILFLI FFECGISFSL PFFLITLLFL MFDVEIILLF VLVILFLLIL LMEWYYGSLV WMKLLFLLSA LSLCLFFFDA YSASFSSVVL LISASIVFYS

Ls SVTFTIFLLN FFECGFDFSL RFFLITIIFL IFDVEITFIL PMFILILILL LHEWNQGALE WSKSLIFFFF GIILVILFDW MSMFFLSLVT FISCLISNYS

Bm WMIFVVSLLV FFECGMEMNM QFFMIGILFL IFDIEVVVML PLIAAILIIM WMEIFMGSLY WKIFFFCFVY LIISTIMFDQ LSVSFMFMVL SVSTCVLIYA

Cb FASVGIVFLS FYECGIEFCM HFFLVGVLFL LFDVELIVCI PMFFFIIFVL VLEMVMGTFD WKEGLFGFVG GLFFLLVVDS FSMIFLFTVG MVSSFVLLYS

Cs FFLAGIFILS FYECGIEFYL HFFLIGVLFL LFDVELVVCI PMFFFILFVL VMELVMGTFS WKESLLSFFG GVFFILLFDL FSMMFLFTVG LVSGFVLLYS

Hm LSSLIILYLS VFECGFRFSM QFFSIALVFL IFDVELIIIL PYMMILLYLT LLEWMEGSLD WYYYMSGMMY ILMHNFIFDK FSMIFLFMVL IISKNVLKYS

Ib WIVITVLVLC FFECGMEYCL HFFLIGVLFL VFDMEIIICI PMMMIVLIVY YLELAIGTLN WKELIMSFFM WMLYSMIVDK YSLIFMIMVT MISTIVMIYS

Pc FFTILIIILA LYECGVMTHI HFYVVSVVFL IFDVELVATL PVIPLILTLL LLELHYGSLD WKCILSLLIQ FCFVIIVLDN LSLTFLLMVL TISSLVMAYS

Ph FFTILIIILA LYECGVMTHI HFYVVSVVFL IFDVELVATL PVIPLILTLL LLELHYGSLD WKCILSLLIQ FCFVIIVLDN LSLTFLLMVL TISSLVMAYS

Pp GLVVVVAVFA FFECGVMFYV QFYCMSVIFL VFDVEIVILL PMTFLLLFML LIEIGYGSLE WKLLLGLCLQ FLFLVFIFDR VSVMFLMMVL VVSAVVMVFG

Dm IALLILLIVM FFECGFDFSL RFFLITIIFL IFDVEIALIL PMFILILLIL YHEWNQGMLN WSNSLSCFLL SLYFTFLFDW MSLLFMSFVL MISSLVIFYS

Ld LSYMSKEKDK NIFFYVLFFF SLSMQILIFS FSFSSILLGW DGLGMTSFYL IIFYHNVKSL HGSMVTVLTN RLGDCFVLLA SLTKSAQLPF SFWLPKAMAA

Lb MSYMQQEKEK IKFFLTLYMF ILSMLILIFS FNISSLLVGW DGLGVTSFLL IYYYHSLKST NSSLITLTLN RVGDLMIMTA ALSKSAQLPF SSWLPLAMAA

Ls NSYMLGDNNS KLFMFLIIMF VFSMMLMIVS PNMISILLGW DGLGLVSYIL VIYYQNVKSY NAGMLTVLSN RIGDIMIIIA GMTKSAQIPF SSWLPAAMAA

Bm VWYMEGEKNF NKFIVTLFMF IISMMFLCMS TDIYWVMVGW DGLGITSFFL IIFFQNWKSV SSGMVTLLSN RIGDVFIALG AITKSAQYPY SAWLPEAMAA

Cb NYYMMGSLFK KKFILVMMIF ILSMFVLSLS GDLFWVMIGW DGLGFSSMCL IFFFQNWKSF NSSMVTFISN RIGDFLICFG ALSKSAQVPF SAWLPLAMAA

Cs NYYMKESLFK KKFIVVMMVF IFSMFLLSMS GDLVWLMVGW DGLGFSSMCL IFFFQNWKSF NSAMVTFISN RIGDFFICFG AFSKSAQVPF SAWLPLPMAA

Hm YFYFVGTVWT LRFIGILIFF IVSMLWLIMS YDMFTFIVGW DMLGVSSFLL ILYYNSYKSK KSSLITYISN RFGDGFFFCT SITKSAQFPF SSWLPEAMAA

Ib IYYMMEEKMK KKFFLSMFFF ILSMMILSFS ANIFWLMVGW DGLGLSSFIL IMYFQNWNSF NSSMTTFMCN RFGDLFMIVC AMTKSAQVPF SVWLPLAMAA

Pc NYYMAGHNLG GDFYVSMVLF IVSMLLLSLS GSMFWSFIGW DGLGMMSLVL ILFNKSWSSQ KSGVITFLMN RLGDSFMLIG GASKSAQFPF SSWLPEAMAA

Ph NNYMAGHNLG GDFYVSMVLF IVSMLLLSLS GSMFWSFIGW DGLGMMSLVL ILFNKSWSSQ KSGVITFLMN RLGDSFMLIG GASKSAQFPF SSWLPEAMAA

Pp SFYMTSSLHG VAFTFSMLIF IASMVILSVT GSLFWLFLGW DGLGLSSFIL IIFNKNWSSS KSGLITFLMN RLGDVLMTIG CLSKSAQFPL LSWLPEAMAA

Dm KEYMMNDNHI NRFIMLVLMF VLSMMLLIIS PNLISILLGW DGLGLVSYCL VIYFQNIKSY NAGMLTALSN RIGDVALMLA AMTKSAQIPF SSWLPAAMAA

Ld PTPVSSLVHS STLVTAGVYI LFRFILSFST LIMGGFMAIK SFDLKEIVAF STLSQVGLMM FFMSLSLKEV AMFHLLTHAL FKSVLFMCSG YLIQDISPVL

Lb PTPVSSLVHS STLVTAGVYL LYRAFITSLT LIMSSVLALQ SFDLKEIVAF STMSHISLMM MGISNGLYKF SFFHLCTHAL FKALLFMCSG YLIQDINYKV

Ls PTPVSALVHS STLVTAGVYL LIRFFISGLT MFMSGLGANF EFDLKKIIAL STLSQLGLMM GSLSMGLTNF CFFHLLSHAL FKALLFMCAG YLIQDILPFV

Bm PTPVSALVHS STLVTAGIYL LLRFSVASMS AFLSISSSWG ELDLKKIIAL STLSHLSMMI LFISCKDYLC AIIHMISHAF FKSSLFMFAG ILIQDITHFL

Cb PTPVSSLVHS STLVTAGVFL LIRFLVSFIT IFLAGMSSVG EYDLKKVIAL STLSHIGLMM MFVGMESFIS AKIHLVIHAF FKSLLFMISG FIIQDINYFF

Cs PTPVSSLVHS STLVTAGVFL LIRFFGSFLT FVLAGMSSLS EYDLKKIIAL STLSHMGLMM SFVGMESFVS AKIHLINHAF FKSLLIMTSG FMIQDINFFF

Hm PTPVSTLVHS STLVTAGFYF LFRFFISLFT MTLASSAALM EYDLKKVIAL STLSQISFMF FSLSLKLTTL AFFHMVMHAF FKAATFMIAG VIIQDLNKLL

Ib PTPVSSLVHS STLITAGVFL CIRFYISSFT FIMSGLSAMY EYDLKKIIAL STLSHMALIF FFLSMNSFES SMIHLITHAI FKSSLFMSAG VLIQDINMKL

Pc PTPVSSLVHS STLVTAGIYV LARYYLSSIS IIISGVSALW SSDLKKVVAY STLSHISLML FYLSEGSVEG ALIHMLTHSV FKSLLFMTLG SSIQDSTFSM

Ph PTPVSSLVHS STLVTAGIYV LARYYLSSIS IIISGVSALW SSDLKKVVAY STLSHISLML FYLSEGSVEG ALIHMLTHSV FKSLLFMTLG SSIQDSTFSM

Pp PTPVSSLVHS STLVTAGIYT LVRFAFSFFS LVVSGLSALV STDLKKVVAY STLSHISLMI HYLSEGCIDA CMLHMVMHAI FKSLLFMSVG SVIQDTSTGL

Dm PTPVSALVHS STLVTAGVYL LIRFLLSGLT MFMAGLGANF EFDLKKIIAL STLSQLGLMM SILSMGFLKL AMFHLLTHAL FKALLFMCAG AIIQDIMPLT

Ld KLSIIISGLP FLSGFYSKDL YTLRLINFIM GSIFMGSSIQ WTIMILYFVI VMIMFILPLS ILVIMMMWFF FLVISTSVKW LVYVILIFII GGLMISFSYT

Lb KYIFFIMGLP FMAGFYSKDG YTMRLLYYAW FSIFSGAAIQ WSLIMLIMSS LIKTLVWGLS LVTIMAVYMF VMIFLGSGIW MSMIFLIFMI GGLMVSFFYM

Ls SLCFNVCGIP FLSGFYSKDL YSFRLFSFFL GSLFGGSMLM WLVLFLILIG IFKIYLFIFI YIYMFVLILI MSTILVALMW FSYILTLILI GGMLILFIYM

Bm SMSLVFVGGP FLAGFYSKEI YSARIIFRTI LSISAGSMII WLSKLFLFLS MFMFLCGVMI LMVSIFIYLL VSTTIFTTIF VGFLFFLSTI SGLFILFSIF

Cb SMTFLLMGAP FFSGFFSKEI YSIRMLFYSV VSSFLAGVMG FVFSSFRKVG LLIYLKVCCF LGLSIFLMVL VFSVISTLSV PLFMVVLTLS GGLFVLVSFV

Cs SLSFILGGAP FFSGFFSKEI YSIPALYYSI VSSFFKGLIL LKFSLFQKMS MKVFVKMCCW NGNSLFFMVL LFSIFSSLCV PLYMVVLTLS GGLFVLVSLV

Hm LLLLLFSGFL FLSGFYSKDL YCLRMCLMML LSIVSGSVLM WYLFMIMILM PMMILEFFFL LMILFLILVL INIISVSGLF EGMILVIVFL TGLFILMSYL

Ib IFMIIIMGIL FLSGFYSKEL YSTRLLYISS SISIFLGGFL LKLFWLILFF MFYFQLLSMV LMFLQFFLMI ISFLMFGVLF LSLMFFLMTL GGLLILFSFI

Pc ASLVGVAGLP FLSGGYSKEV YSFRIIYLNV LISAWISSSP GYMLFMILFG FITMPAIVFI ISLNWLFSLA MTVFTVGLLW SWLLLWLGIL GGLIVSLSMA

Ph ASLVGVAGLP FLSGGYSKEV YSFRIIYLNV LISAWISSSP GYMLFMILFG FITMPAIVFI ISLNWLFSLA MTVFTVGLLW SWLLLWLGIL GGLIVSLSMA

Pp TTFALLAGLP YLSGGFSKEV YSVRIMWINM MVVFWAVWTP FWMISVASLG GCTIPSLTMF LSLNWLLDMA FMGVIIKVLW PWLLLLLGVV GGLIVVISVI

Dm SACFNVCGMP FLAGFYSKDM YSFRLVYYLI MSIIGGSMLN WLTLFVCIVG GLKIYLLLFV FWILILLTLL IQTIFVCLLW YSYILFLIFL GGMLVLFIYV

Ld VSGLVGSIYL FMLL

Lb VSVLFISVCM LFLL

Ls ISFLIIPMML TIIS

Bm MMFMIILSSL IFLL

Cb IMYSCLSMVF ILLL

Cs VMFSSVFLFL IMLL

Hm VSYFMKWILM VNIL

Ib PMFYLFIMIL MLIM

Pc FIFWILTSVY TAVV

Ph FIFWILTSVY TAVV

Pp LVFVLVLILL VLLI

Dm TSLILIFMLI TILL
