# Supplementary material for: The Complete Mitochondrial Genome of the Booklouse, Liposcelis decolor: Insights into Gene Arrangement and Genome Organization within the Genus Liposcelis
Source: PLoS One. 2014 Mar 17;9(3):e91902. doi: 10.1371/journal.pone.0091902 (PMC3956861; doi:10.1371/journal.pone.0091902)
Supplement: Table S1 — PCR primers used for the amplification of the mitochondrial genome of Liposcelis decolor . (DOC) [file pone.0091902.s004.doc]

Table S1. PCR primers for amplification of mitochondrial genome of *Liposcelis decolor*

| Gene | Primer | Primer sequence (5’-3’) | Primer position | Product size (bp) |
| --- | --- | --- | --- | --- |
| *cox1* | UEA5 | AGTTTTAGCAGGAGCAATTACTAT |  | UEA5-UEA8: 791 |
| *cox1* | UEA8 | AAAAATGTTGAGGGAAAAATGTTA |  |
| *cox3* | C3-J-5014 | TTATTTATTGCATCAGAAGT |  | C3-J-5014-C3-N-5460: 445 |
| *cox3* | C3-N-5460 | TCAACAAAGTGTCAGTATCA |  |
| *cob* | CBF1 | TATGTACTACCATGAGGACAAATATC |  | CBF1-CBR1: 432 |
| *cob* | CBR1 | ATTACACCTCCTAATTTATTAGGAAT |  |
| *rrnL* | 16Sar | CGCCTGTTTAACAAAAACAT |  | 16Sar-16Sbr: 464 |
| *rrnL* | 16Sbr | CCGGTCTGAACTCAGATCACGT |  |
| *rrnS* | 12SRNA-F | AAACTAGGATTAGATACCCTATTAT |  | 12SRNA-F-12SRNA-R: 328 |
| *rrnS* | 12SRNA-R | AAGAGCGACGGGCGATGTGT |  |
| *nad5* | N5-J7077 | TTAAATCCTTWGARTAAAAYCC |  | N5-J7077-N5-N7793: 688 |
| *nad5* | N5-N7793 | TTAGGTTGRGATGGNYTAGG |  |
| *cox3* | d1 | TTCAGGATTGACAATTACTAGAGCG | 4099-4123 | d1-d2:9052 |
| *rrnS* | d2 | AAACATCTCAGAGGAATATGGGGT | 9428-9451 |
| *rrnS* | d3 | TACCTGAACAGTTGGTCGAAAAAC | 9544-9567 | d3-d4: 5478 |
| *cox3* | d4 | AGTTAGAGAGGTCCATATCATAGC | 4042-4065 |
| *cob* | d5 | TATAGTGGAATGATTATGGGGTGGT | 13538-13562 | d5-d2: 4086 |
| *nad5* | d6 | CAGCCCCAACTCCTGTATCTTCTC | 3127-3150 | d6-d7: 4268 |
| *cob* | d7 | GAGGAGGGGTCAATATAGGGTTAG | 13240-13263 |
| *nad5* | d8 | GCACGAGAGAAGATACAGGAGTTG | 3121-3144 | d8-d9: 5419 |
| *rrnL* | d9 | CAGACCTCAATAAAAAGCCAAGTG | 8561-8584 |
| *nad4L* | sd1 | ACTAAAGTCAAGCCAACCAC | 7060-7079 | sd1-d9: 1481 |
| *atp6* | sd2 | GGACACCCTAAAGGTACTAAG | 6493-6513 | sd2-sd3: 1022 |
| *nad3* | sd3 | CCGCAAAAATCACAAAAGGT | 7536-7555 |
