# Supplementary material for: The Complete Mitochondrial Genome of the Booklouse, Liposcelis decolor: Insights into Gene Arrangement and Genome Organization within the Genus Liposcelis
Source: PLoS One. 2014 Mar 17;9(3):e91902. doi: 10.1371/journal.pone.0091902 (PMC3956861; doi:10.1371/journal.pone.0091902)
Supplement: Table S2 — GenBank accession numbers of all the insects mentioned in this study. (DOC) [file pone.0091902.s005.doc]

Table S2. GenBank accession numbers of all of the insects mentioned in this study

| Classification | Species | GenBank acc. No. | |
| --- | --- | --- | --- |
| Psocoptera: Liposcelidae | *Liposcelis decolor* | | In this study |
|  | *Liposcelis bostrychophlia* | | JN645275, JN645276 |
| Psocoptera: Lepidopsocidae | Lepidopsocid sp. | | AF335994 |
| Phthiraptera: Ischnocera: Philopteridae | *Bothriometopus macrocnemis* | | EU183542 |
|  | *Campanulotes bidentatus* | | AY968672 |
|  | *Coloceras* sp. | | JN122000 (14868 bp);  JN122001 (7650 bp) |
|  | *Ibidoecus bisignatus* | | JN122005 |
|  | *Anaticola crassicornis* | | JN121999 |
|  | *Philopterus* sp. | | JN122006 |
|  | *Quadraceps* sp. | | JN121998 |
| Phthiraptera: Ischnocera: Trichodectidae | *Damalinia meyeri* | | JN122002~ JN122004 |
| Amblycera | *Heterodoxus macropus* | | AF270939 |
| Phthiraptera: Anoplura: Pediculidae | *Pediculus humans* | | FJ499473 ~ FJ499490 |
|  | *Pediculus capitis* | | JX080388~ JX080407 |
| Phthiraptera: Anoplura: Pthiridae | *Pthirus pubis* | | JQ976018,  EU219987~ EU219995,  HM241895~HM241898 |
| Monogononta: Ploimida: Brachionidae | *Brachionus plicatilis* | | AP009407~ AP009408 |
