# Supplementary material for: The Complete Mitochondrial Genome of the Booklouse, Liposcelis decolor: Insights into Gene Arrangement and Genome Organization within the Genus Liposcelis
Source: PLoS One. 2014 Mar 17;9(3):e91902. doi: 10.1371/journal.pone.0091902 (PMC3956861; doi:10.1371/journal.pone.0091902)
Supplement: Table S3 — Summary of the mitochondrial genome of Liposcelis decolor . aGenes located in the different strand from that of cox1 are underlined. binc = intergenic nucleotides, indicating gap nucleotides (positive value) and overlapping nucleotides (negative value) of two adjacent genes. cAT-skew = (A−T)/(A+T), GC-skew = (G−C)/(G+C). dCR = control region (putative). (DOC) [file pone.0091902.s006.doc]

Table S3. Summary of the mitochondrial genome of *Liposcelis decolor*.

| gene ^a^ | region | size | inc ^b^ | AT% | AT-skew ^c^ | GC-skew ^c^ | start codon | stop codon |
| --- | --- | --- | --- | --- | --- | --- | --- | --- |
| *cox1* | 1-1542 | 1542 | 8 | 68.22 | -0.260 | 0.065 | TTG | TAA |
| *trnN* | 1551-1616 | 66 | 8 | 74.24 | 0.102 | 0.294 |  |  |
| *trnL2* | 1648-1715 | 68 | 31 | 66.18 | 0.156 | -0.044 |  |  |
| *trnT* | 1729-1797 | 69 | 13 | 69.57 | -0.167 | 0.143 |  |  |
| *trnE* | 1809-1872 | 64 | 11 | 82.81 | 0.019 | 0.273 |  |  |
| *trnG* | 1924-1988 | 65 | 51 | 73.85 | 0.083 | 0.059 |  |  |
| *trnL1* | 2058-2126 | 69 | 69 | 73.91 | 0.059 | 0.222 |  |  |
| *nad5* | 2159-3739 | 1581 | 32 | 77.42 | -0.155 | -0.031 | ATA | TAA |
| *cox3* | 3754-4545 | 792 | 14 | 72.47 | -0.202 | -0.018 | ATA | TAA |
| *nad1* | 4556-5440 | 885 | 10 | 74.46 | -0.211 | 0.035 | ATA | TAA |
| *cox2* | 5476-6165 | 690 | 35 | 73.91 | -0.161 | -0.022 | ATT | TAA |
| *trnI* | 6165-6228 | 64 | -1 | 73.44 | -0.064 | 0.294 |  |  |
| *atp6* | 6225-6872 | 648 | -4 | 76.54 | -0.218 | -0.013 | ATG | TAA |
| *atp8* | 6835-6981 | 147 | -38 | 78.91 | -0.310 | 0.032 | ATA | TAG |
| *nad4L* | 6990-7292 | 303 | 8 | 73.60 | -0.202 | 0.125 | ATA | TAA |
| *nad3* | 7321-7653 | 333 | 28 | 75.38 | -0.195 | 0.024 | ATG | TAA |
| *trnK* | 7681-7745 | 65 | 27 | 86.15 | 0.000 | 0.111 |  |  |
| *trnS1* | 7751-7812 | 62 | 5 | 74.19 | -0.130 | -0.125 |  |  |
| *trnS2* | 7842-7904 | 63 | 29 | 80.95 | 0.020 | 0.167 |  |  |
| *trnD* | 7907-7967 | 61 | 2 | 67.21 | -0.073 | 0.100 |  |  |
| *rrnL* | 7968-9048 | 1081 | 0 | 76.32 | 0.001 | 0.219 |  |  |
| *rrnS* | 9049-9788 | 740 | 0 | 69.46 | 0.148 | 0.062 |  |  |
| *nad4* | 9799-11025 | 1227 | 10 | 78.05 | -0.177 | 0.073 | ATA | TAA |
| *nad2* | 11106-11978 | 873 | 80 | 78.81 | -0.142 | 0.016 | ATA | TAA |
| *trnF* | 11992-12058 | 67 | 13 | 82.09 | -0.018 | 0.333 |  |  |
| *trnQ* | 12064-12128 | 65 | 5 | 87.69 | 0.053 | 0.025 |  |  |
| *trnP* | 12129-12197 | 69 | 0 | 73.91 | -0.020 | 0.333 |  |  |
| *nad6* | 12227-12646 | 420 | 29 | 75.95 | -0.166 | 0.129 | ATG | TAA |
| *trnV* | 12656-12722 | 67 | 9 | 77.61 | -0.039 | 0.333 |  |  |
| *trnM* | 12723-12790 | 68 | 0 | 60.29 | -0.122 | -0.185 |  |  |
| *trnH* | 12799-12865 | 67 | 8 | 85.07 | 0.123 | 0.400 |  |  |
| *trnA* | 12868-12931 | 64 | 2 | 82.81 | -0.057 | 0.273 |  |  |
| *cob* | 12932-13993 | 1062 | 0 | 72.22 | -0.265 | 0.058 | ATA | TAA |
| *CR^d^* | 13994-14111 | 118 | 0 | 82.20 | -0.052 | -0.143 |  |  |
| *trnY* | 14112-14181 | 70 | 0 | 65.71 | 0.044 | 0.167 |  |  |
| *trnW* | 14200-14263 | 64 | 18 | 76.56 | 0.102 | 0.067 |  |  |
| *trnR* | 14271-14336 | 66 | 7 | 80.30 | 0.057 | 0.077 |  |  |
| *trnC* | 14335-14397 | 63 | -2 | 77.78 | 0.102 | 0.286 |  |  |

^a^ genes located in the different strand from that of *cox1* are underlined. ^b^ inc = intergenic nucleotides, indicates gap nucleotides (positive value) or overlapped nucleotides (negative value) between two adjacent genes. ^c^ AT-skew = (A-T)/(A+T), GC-skew = (G-C)/(G+C). ^d^CR = control region.
