# Supplementary material for: The Complete Mitochondrial Genome of the Booklouse, Liposcelis decolor: Insights into Gene Arrangement and Genome Organization within the Genus Liposcelis
Source: PLoS One. 2014 Mar 17;9(3):e91902. doi: 10.1371/journal.pone.0091902 (PMC3956861; doi:10.1371/journal.pone.0091902)
Supplement: Table S4 — Start and stop codons of mitochondrial protein-coding genes of the Psocodea. Species are abbreviated as follows: Ld, Liposcelis decolor; Lb, Liposcelis bostrychophila; Ls, Lepidopsocidae sp. RS-2001; Bm, Bothriometopus macrocnemis; Cb, Campanulotes bidentatus; Cs, Coloceras sp. SLC-2011; Hm, Heterodoxus macropus; Ib, Ibidoecus bisignatus; Pc, Pediculus capitis; Ph, Pediculus humanus; Pp, Pthirus pubis; Ac, Anaticola crassicornis; Dm, Damalinia meyeri; Ps, Philopterus sp. SLC-2011. (DOC) [file pone.0091902.s007.doc]

Table S4. The start and stop codons of sequenced mitochondrial genes in Psocodea

|  | *Ld* | *Lb* | *Ls* | *Bm* | *Cb* | *Cs* | *Hm* | *Ib* | *Pc* | *Ph* | *Pp* | *Ac* | *Dm* | *Ps* |
| --- | --- | --- | --- | --- | --- | --- | --- | --- | --- | --- | --- | --- | --- | --- |
| atp6 | ATG/TAA | ATA/TAA | ATA/TAA | ATG/TAA | ATA/TAG | ATT/TAA | ATA/TAA | ATT/TAA | ATG/TAA | ATG/TAA | ATG/TAA |  |  |  |
| atp8 | ATA/TAG | GTG/TAG | ATT/TAA | ATT/TAG | ATG/TAA | ATG/TAG | TTG/TAA | ATT/TAA | ATG/TAA | ATG/TAA | ATG/TAG |  |  |  |
| cob | ATA/TAA | ATT/TAA | ATG/TAA | ATT/TAA | ATA/TAA | ATA/TAG | ATC/TAA | ATT/TAA | ATA/TAA | ATA/TAA | ATT/T |  |  |  |
| cox1 | TTG/TAA | ATC/TAA | ATA/TAA | ATT/TAA | ATT/TAA | ATT/TAG | ATG/TAA | ATG/TAA | ATA/TAA | ATA/TAA | ATT/TAA | ATG/TAA | ATG/TAG | GTT/TAG |
| cox2 | ATT/TAA | ATA/TAA | ATG/TAA | ATG/TAA | ATA/TAA | ATT/TAG | ATA/TAA | ATA/TAA | ATG/TAA | ATG/TAA | ATC/TAA |  |  |  |
| cox3 | ATA/TAA | ATA/TAA | ATG/TAA | TTA/TAA | ATA/TAA | ATA/TAG | ATG/TAG | ATT/TAA | ATG/TAG | ATG/TAG | ATG/TAA |  |  |  |
| nad1 | ATA/TAA | ATC/T | ATA/TAG | ATA/TA | ATT/TAA | ATT/T | ATA/TAA | ATT/TAA | ATG/TAA | ATG/TAA | GTG/TAG | ATG/TAA |  |  |
| nad2 | ATA/TAA | ATT/TAA | ATG/TAA | CTG/TAA | ATG/TAA | ATT/T | ATA/TAA | ATT/TAA | ATA/TAG | ATA/TAG | ATC/T |  |  |  |
| nad3 | ATG/TAA | ATG/TAG | ATA/TAA | ATG/TAA | ATG/TAA | ATG/TAG | ATT/TAA | ATT/TAA | ATT/TAA | ATT/TAA | TTG/TAA | ATA/TAG |  |  |
| nad4 | ATA/TAA | ATC/TAA | ATG/TAA | ATT/TAA | ATA/TAA | ATT/TAA | ATT/TAA | ATA/TAA | ATT/TAA | ATT/TAA |  | ATA/TAG |  |  |
| nad5 | ATG/TAA | ATT/TAG | ATA/TAA | ATT/TAA | ATT/TAA | ATT/TA | ATA/TAA | ATT/T | ATA/TAA | ATA/TAA | ATT/TAA |  |  |  |
| nad6 | ATG/TAA | ATT/TAA | ATT/TAA | ATA/TAG | ATA/TAA | ATT/TAA | ATC/TAA | ATT/TAG | ATA/TAA | ATA/TAA | TTG/TAA | ATT/TAA |  | TTA/T |
| nad4L | ATA/TAA | ATT/TAA | ATG/TAA | ATA/TAA | ATT/TAA | ATT/TAA | ATA/TAA | ATT/T | ATA/TAA | ATT/TAA | ATT/TAG |  |  |  |

Species are abbreviated as following: *Ld*: *Liposcelis decolor*; *Lb*: *Liposcelis bostrychophila*; *Ls*: Lepidopsocidae sp. RS-2001; *Bm*: *Bothriometopus macrocnemis*; *Cb*: *Campanulotes bidentatus*; *Cs*: *Coloceras* sp. SLC-2011; *Hm*: *Heterodoxus macropus*; *Ib*: *Ibidoecus bisignatus*; *Pc*: *Pediculus capitis*; *Ph*: *Pediculus humanus*; *Pp*: *Pthirus pubis*; *Ac*: *Anaticola crassicornis*; *Dm*: *Damalinia meyeri*; *Ps*: *Philopterus* sp. SLC-2011.
