# Supplementary material for: The Complete Mitochondrial Genome of the Booklouse, Liposcelis decolor: Insights into Gene Arrangement and Genome Organization within the Genus Liposcelis
Source: PLoS One. 2014 Mar 17;9(3):e91902. doi: 10.1371/journal.pone.0091902 (PMC3956861; doi:10.1371/journal.pone.0091902)
Supplement: Table S5 — Nucleotide compositions of the mitochondrial genomes of Liposcelis decolor and L. bostrychophila . (DOC) [file pone.0091902.s008.doc]

Table S5. Nucleotide compositions of the mitochondrial genomes of *Liposcelis decolor* and *L. bostrychophlia*

| Region | Length (bp) | |  | A% | |  | T% | |  | G% | |  | C% | |  | AT% | |
| --- | --- | --- | --- | --- | --- | --- | --- | --- | --- | --- | --- | --- | --- | --- | --- | --- | --- |
|  | *Ld* | *Lb* |  | *Ld* | *Lb* |  | *Ld* | *Lb* |  | *Ld* | *Lb* |  | *Ld* | *Lb* |  | *Ld* | *Lb* |
| Full length | 14405 | 16463 |  | 40.49 | 30.21 |  | 34.74 | 38.42 |  | 12.63 | 13.16 |  | 12.14 | 18.21 |  | 75.23 | 68.63 |
| PCGs | 10503 | 10366 |  | 30.03 | 27.40 |  | 44.74 | 40.54 |  | 13.06 | 14.09 |  | 12.17 | 17.97 |  | 74.77 | 67.93 |
| 1st codon | 3501 | 3456 |  | 34.10 | 31.54 |  | 37.22 | 33.88 |  | 17.77 | 17.95 |  | 10.91 | 16.93 |  | 71.32 | 65.42 |
| 2nd codon | 3501 | 3455 |  | 21.88 | 18.35 |  | 48.41 | 49.35 |  | 12.88 | 13.49 |  | 16.82 | 18.81 |  | 70.29 | 67.70 |
| 3rd codon | 3501 | 3455 |  | 34.10 | 32.30 |  | 48.59 | 38.38 |  | 8.54 | 11.14 |  | 8.77 | 18.18 |  | 82.69 | 70.68 |
| tRNA genes | 1449 | 1387 |  | 38.44 | 36.27 |  | 37.41 | 36.70 |  | 13.80 | 15.07 |  | 10.35 | 11.97 |  | 75.85 | 72.96 |
| rRNA genes | 1821 | 1759 |  | 38.88 | 35.19 |  | 34.65 | 35.19 |  | 15.16 | 17.68 |  | 11.31 | 11.94 |  | 73.53 | 70.38 |
| Non-coding | 680 | 2158 |  | 41.76 | 29.15 |  | 43.68 | 38.88 |  | 7.06 | 12.23 |  | 7.50 | 19.74 |  | 85.44 | 68.03 |
